# Localized inhibition of protein phosphatase 1 by NUAK1 promotes spliceosome activity and reveals a MYC-sensitive feedback control of transcription

**DOI:** 10.1016/j.molcel.2021.05.013

**Published:** 2021-06-03

**Authors:** Giacomo Cossa, Isabelle Roeschert, Florian Prinz, Apoorva Baluapuri, Raphael Silveira Vidal, Christina Schülein-Völk, Yun-Chien Chang, Carsten Patrick Ade, Guido Mastrobuoni, Cyrille Girard, Amit Kumar, Lars Wortmann, Susanne Walz, Reinhard Lührmann, Stefan Kempa, Bernhard Kuster, Elmar Wolf, Dominik Mumberg, Martin Eilers

(Molecular Cell *77*, 1322–1339.e1–e11; March 19, 2020)

After discussion among the co-authors, who are all in agreement, Amit Kumar’s contribution to this paper warranted inclusion as a co-author. The authors regret this omission. The paper has since been corrected online, including the author list, acknowledgments, and author contributions, which also appear here.

