# Supplementary material for: Localized Inhibition of Protein Phosphatase 1 by NUAK1 Promotes Spliceosome Activity and Reveals a MYC-Sensitive Feedback Control of Transcription
Source: Mol Cell. 2020 Mar 19;77(6):1322–1339.e11. doi: 10.1016/j.molcel.2020.01.008 (PMC7086158; doi:10.1016/j.molcel.2020.01.008)
Supplement: Document S1. Figures S1–S7 and Tables S1–S4 [file mmc1.pdf]

Molecular Cell, Volume *77*

Supplemental Information

Localized Inhibition of Protein Phosphatase 1 by NUAK1 Promotes Spliceosome Activity and Reveals a MYC-Sensitive Feedback Control of Transcription

Giacomo Cossa, Isabelle Roeschert, Florian Prinz, Apoorva Baluapuri, Raphael Silveira Vidal, Christina Schülein-Völk, Yun-Chien Chang, Carsten Patrick Ade, Guido Mastrobuoni, Cyrille Girard, Lars Wortmann, Susanne Walz, Reinhard Lührmann, Stefan Kempa, Bernhard Kuster, Elmar Wolf, Dominik Mumberg, and Martin Eilers

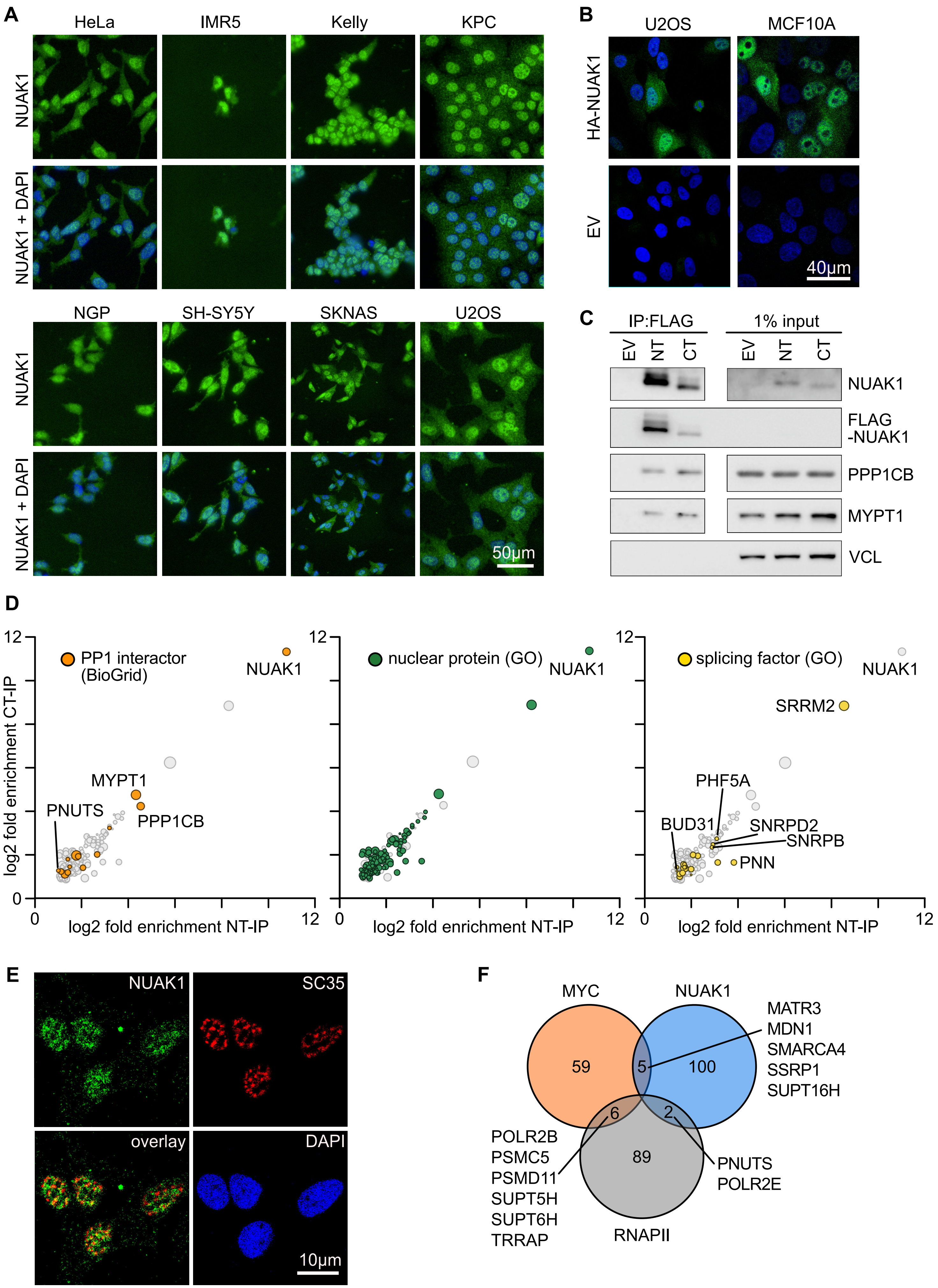

A
HeLa
IMR5
Kelly
KPC
NUAK1
NUAK1 + DAPI
NGP
SH-SY5Y
SKNAS
U2OS
50µm
B
U2OS
MCF10A
HA-NUAK1
EV
40µm
C
IP:FLAG
1% input
EV
NT
CT
NUAK1
FLAG
-NUAK1
PPP1CB
MYPT1
VCL
D
PP1 interactor
(BioGrid)
nuclear protein (GO)
splicing factor (GO)
NUAK1
MYPT1
PNUTS
PPP1CB
SRRM2
PHF5A
BUD31
SNRPD2
SNRPB
PNN
log2 fold enrichment CT-IP
log2 fold enrichment NT-IP
0
12
E
NUAK1
SC35
overlay
DAPI
10µm
F
MYC
NUAK1
RNAPII
59
5
100
6
2
89
MATR3
MDN1
SMARCA4
SSRP1
SUPT16H
POLR2B
PSMC5
PSMD11
SUPT5H
SUPT6H
TRRAP
PNUTS
POLR2E

Figure S1. Characterization of nuclear NUAK1 complexes, Related to Figure 1.

A. Immunofluorescence using an a-NUAK1 antibody of the described cell lines. DAPI is used as nuclear counterstain (n=3).

B. Immunofluorescence using an a-HA antibody of U2OS or MCF10A cells expressing HA-tagged NUAK1 (and their empty vector, EV, counterpart). DAPI is used as nuclear counterstain (n=3).

C. Immunoblots of FLAG immunoprecipitates from U2OS cells expressing amino(NT)-, carboxy(CT)-terminally FLAG-tagged NUAK1 or empty vector (EV). Input corresponds to 1% of samples used for the immunoprecipitation. VCL was used as loading control and as negative control for the IP.

D. Diagram illustrating the presence of PP1 interactors (orange, according to the PPP1CA/B/C BioGrid interactome), nuclear proteins (green, GO term analysis), and splicing factors (yellow, GO term analysis) in the MS analysis of FLAG-NUAK1 IP. Dot size is according to number of peptides identified by MS (average of n=2).

E. Immunofluorescence of endogenous NUAK1 and the nuclear speckle marker SC35 in U2OS cells. DAPI is used as nuclear counterstain.

F. Venn diagram comparing nuclear NUAK1 interactors (n=107) with nuclear interactors of MYC (n=70) and of RNAPII (n=97).

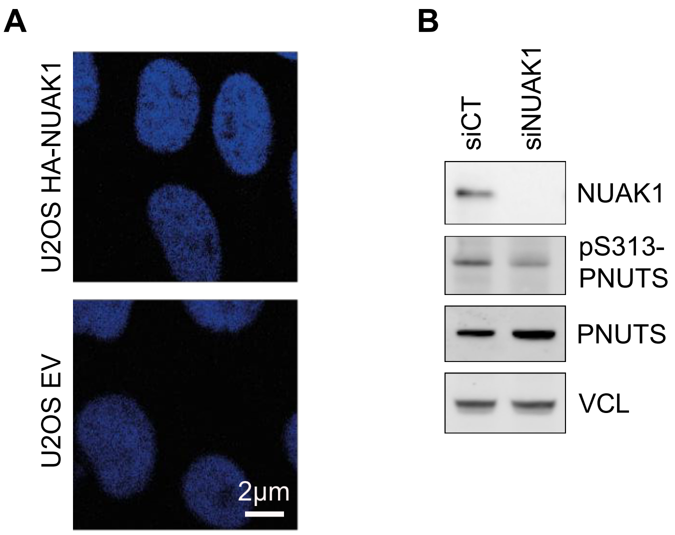

Figure S2. Phosphorylation of PNUTS by NUAK1, Related to Figure 2.

A. Specificity control of the proximity ligation assay (PLA) shown in Figure 2B. The assay was performed using only the HA antibody. DAPI is used as nuclear counterstain (n=3).

B. Immunoblot documenting siRNA-mediated depletion of NUAK1 and phosphorylation of PNUTS. U2OS cells were transfected with a siRNA pool targeting NUAK1 for 48 h. VCL was used as loading control (n=3).

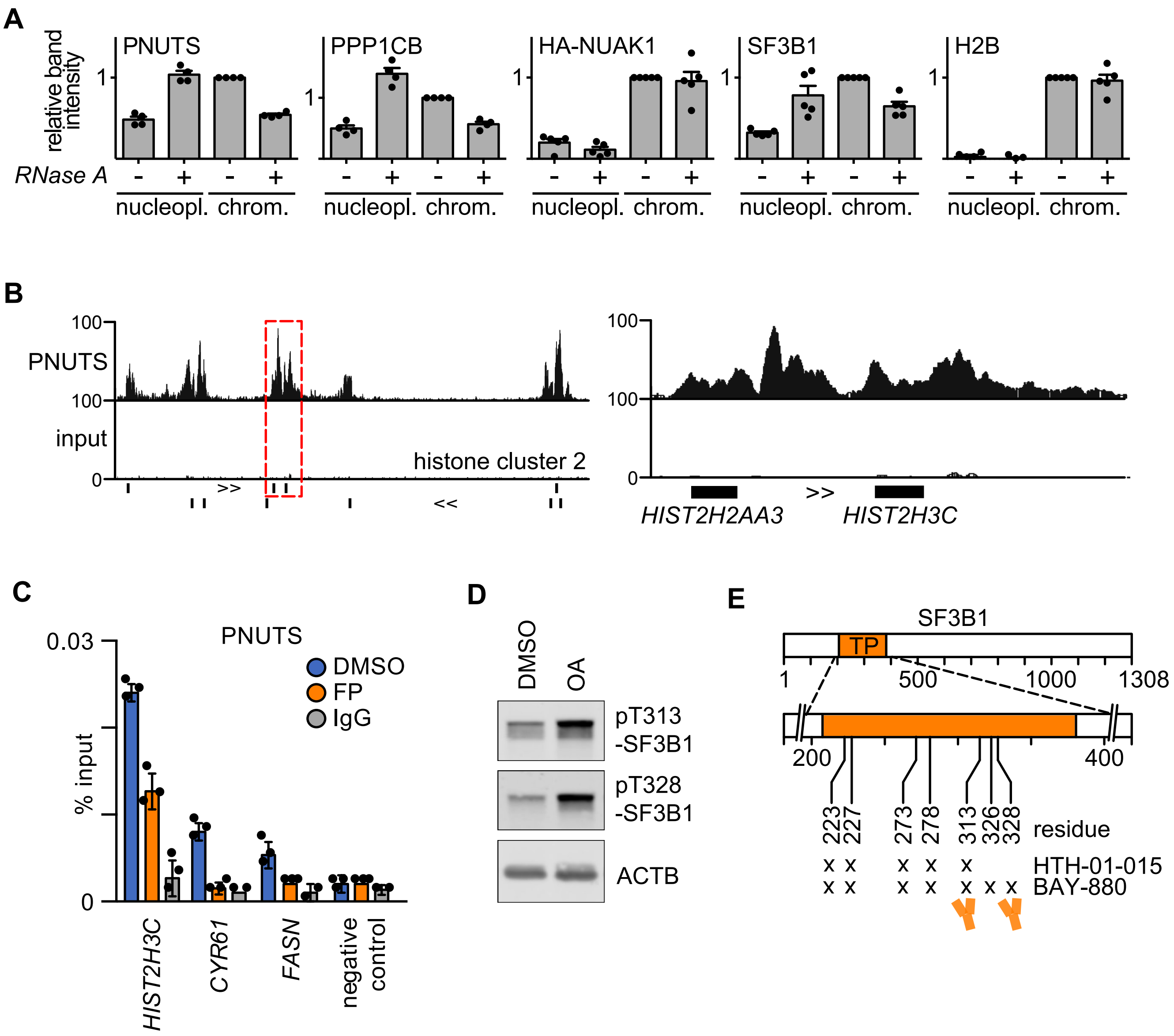

Figure S3. Chromatin association of PNUTS, Related to Figure 3.

A. Quantification of band intensity of selected proteins from the experiment shown in Figure 3A (mean±SEM of biological quadruplicates). “Nucleopl.”, nucleoplasmic fraction; “chromatin”, chromatin-bound fraction. Band intensity was normalized to the non-RNase A-treated, chromatin-bound sample.

B. Left: Genome browser tracks of PNUTS occupancy in a representative histone cluster. Right: Magnification of the section highlighted in the left panel.

C. PNUTS ChIP performed upon 4 h flavopiridol treatment (50nM). IgG ChIP was used as control. Indicated primers refer to gene transcription start site (data show mean±SD of technical triplicates of a representative experiment, n=3).

D. Immunoblot showing phosphorylation of SF3B1 at the indicated sites upon incubation of U2OS cells with 2 µM okadaic acid (OA) for 4 hr. ACTB was used as loading control (n=3).

E. NUAK1-dependent phosphosites in the TP-rich domain of SF3B1. From top downwards: position of the TP-rich domain in the SF3B1 protein; position within the TP-rich domain of the downregulated phosphorylation sites (“x”) identified by phosphoproteomic analysis upon 2 h 10 µM BAY-880 or HTH-01-015 treatment. The two sites recognized by the phospho-specific antibodies are highlighted with an antibody symbol.

A

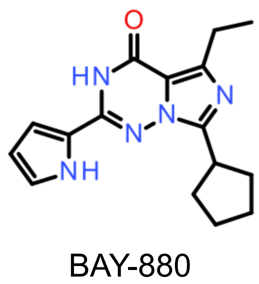

B

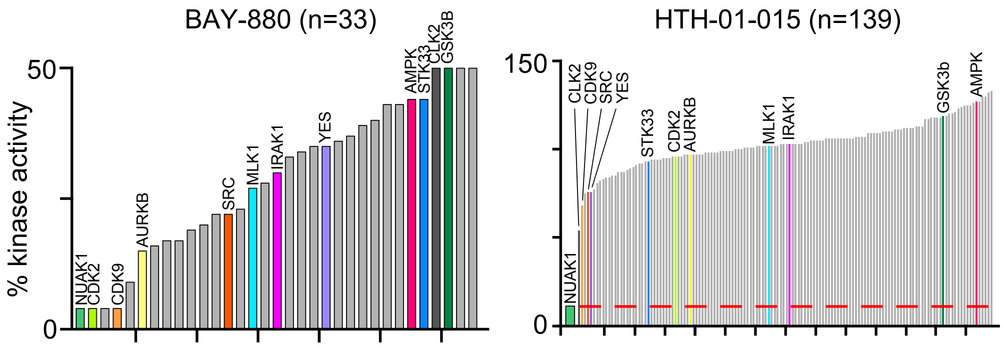

C

	GO Term	siNUAK1 hits	siNUAK1 fold enr.	siNUAK1 FDR	BAY-880 hits	BAY-880 fold enr.	BAY-880 FDR
splicing	RNA splicing	63	4,24	2,96E-17	86	5,95	1,75E-33
	mRNA splicing, via spliceosome	53	4,77	2,67E-16	63	5,84	9,77E-24
metabolism	RNA metabolic process	136	2,16	5,28E-14	209	3,41	7,83E-50
	regulation of RNA met. proc.	248	1,72	8,88E-16	286	2,04	6,85E-31
localization	RNA localization	35	4,23	2,05E-09	46	5,72	5,50E-17
	RNA transport	33	4,45	2,33E-09	43	5,97	1,93E-16
processing	RNA processing	92	2,82	3,38E-15	135	4,26	1,19E-39
	regulation of mRNA proc.	35	6,74	2,51E-14	39	7,73	4,95E-18
RNAPII-related	reg. of transcription by RNAPII	149	1,47	1,37E-04	172	1,74	1,15E-10
	negative reg. transcr. RNAPII	61	1,89	2,41E-04	75	2,39	3,55E-09
3'end processing	mRNA 3'-end processing	16	5,25	2,38E-05	20	6,75	3,00E-08
	RNA 3'-end processing	19	3,77	1,48E-04	23	4,69	5,79E-07
stability	regulation of RNA stability	19	2,73	5,41E-03	30	4,43	1,34E-08
	regulation of mRNA stability	18	2,67	9,25E-03	29	4,43	2,66E-08

D

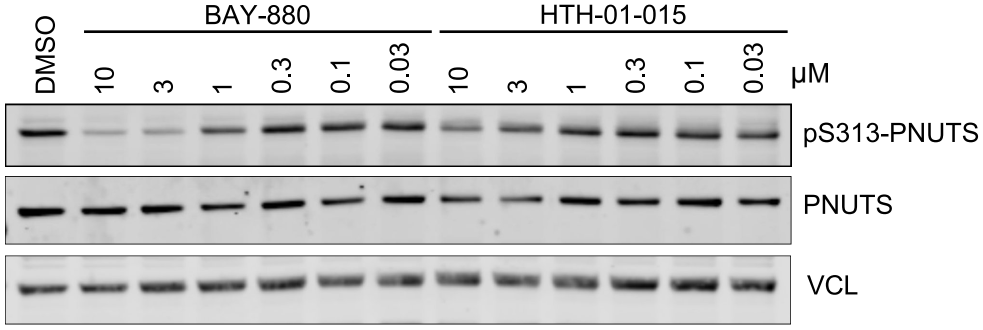

E

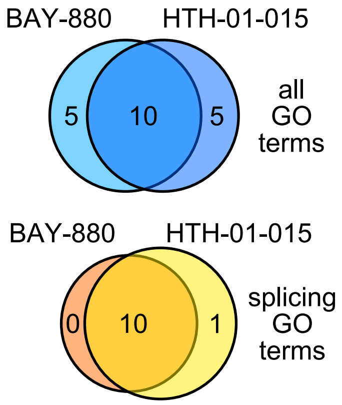

F

GO Term	BAY-880 #	BAY-880 hits	BAY-880 fold enr.	BAY-880 FDR	HTH-01-015 #	HTH-01-015 hits	HTH-01-015 fold enr.	HTH-01-015 FDR
RNA processing	1	64	6.8	4.6E-30	1	47	4.7	1.6E-14
mRNA processing	2	46	9.6	8.0E-27	3	32	5.8	3.8E-12
RNA splicing	3	42	10.3	1.9E-25	2	31	6.8	3.2E-13
mRNA metabolic process	4	50	7.2	2.3E-24	4	37	4.7	1.4E-11
RNA splicing (bulged adenosine)	5	30	10.2	1.7E-17	11	19	5.6	9.4E-07
mRNA splicing, via spliceosome	6	30	10.2	1.8E-17	12	19	5.6	9.6E-07
RNA splicing (transester. rxn)	7	30	10.1	2.0E-17	13	19	5.6	1.0E-06
regulation of mRNA met. proc.	9	24	8.0	1.6E-11	10	20	5.3	8.3E-07
regulation of mRNA processing	11	16	13.0	4.5E-10	6	15	9.5	9.8E-08
regulation of RNA splicing	12	16	12.7	4.8E-10	9	14	8.9	6.5E-07

G

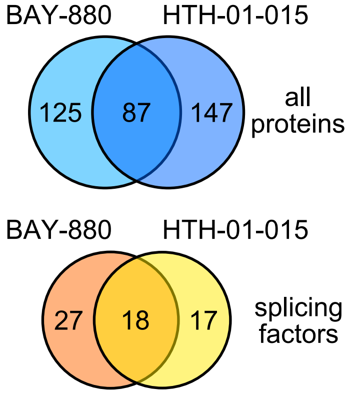

Figure S4. Comparison of NUAK1 inhibitors used in this study, Related to Figure 4.

A. Chemical structure of BAY-880.

B. Left: Histogram showing kinase activity (as percentage of control) upon incubation with 1 µM BAY-880. Only kinases showing less than 50% activity are shown (33 out of 274 screened, listed in Table S1). Right: Activity of 139 kinases previously screened upon treatment with 1 µM HTH-01-015. Data are taken from (Banerjee et al., 2014a). Kinases included in both graphs are highlighted and labeled. Red dashed line, NUAK1 activity level upon HTH-01-015 treatment (11%).

C. RNA-related GO terms of differentially phosphorylated proteins identified in response to siRNA-mediated depletion of NUAK1 (48 h) or 2 h treatment with 10 µM BAY-880. Two GO terms from each of the categories identified in Figure 4D are shown. For each term, the number of identified hits, the fold enrichment and the false discovery rate (FDR) are reported.

D. Immunoblots documenting effect of the indicated concentrations of BAY-880 and HTH-01-015 on PNUTS levels and phosphorylation at S313. U2OS cells were incubated for 24 h. VCL was used as loading control (n=3).

E. The 350 strongest downmodulated phosphosites identified in a label-free phosphoproteomic analysis performed upon 2 h BAY-880 or HTH-01-015 treatment were used as input for a GO term analysis. Top: Venn diagram of top 15 identified GO terms in each condition. Bottom: Same as above, but including only splicing-related GO terms.

F. GO terms of differentially phosphorylated proteins identified in response to both BAY-880 and HTH-01-015. For each term and each treatment, the ranking, the number of identified hits, the fold enrichment and the false discovery rate (FDR) is reported.

G. Top: Venn diagram of proteins with the phosphosites identified as described in Figure 4D. Bottom: Same as above, but including only splicing-related proteins.

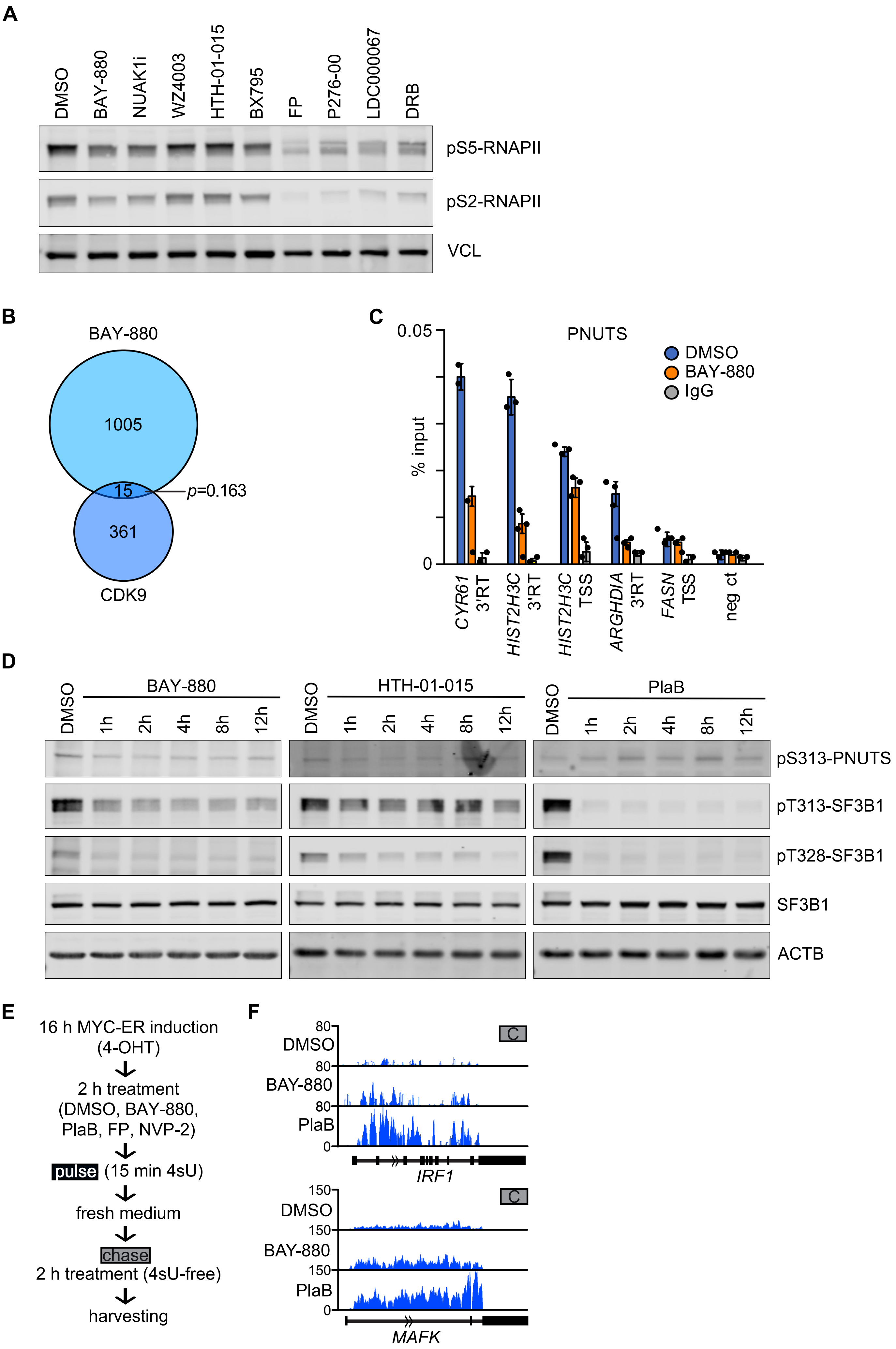
A
DMSO
BAY-880
NUAK1i
WZ4003
HTH-01-015
BX795
FP
P276-00
LDC000067
DRB
pS5-RNAPII
pS2-RNAPII
VCL
B
BAY-880
1005
15
p=0.163
361
CDK9
C
PNUTS
0.05
% input
0
DMSO
BAY-880
IgG
CYR61 3'RT
HIST2H3C 3'RT
HIST2H3C TSS
ARGHDIA 3'RT
FASN TSS
neg ct
D
DMSO
BAY-880
HTH-01-015
PlaB
1h
2h
4h
8h
12h
pS313-PNUTS
pT313-SF3B1
pT328-SF3B1
SF3B1
ACTB
E
16 h MYC-ER induction (4-OHT)
2 h treatment (DMSO, BAY-880, PlaB, FP, NVP-2)
pulse (15 min 4sU)
fresh medium
chase
2 h treatment (4sU-free)
harvesting
F
80
DMSO
BAY-880
PlaB
0
C
IRF1
150
MAFK

Figure S5. Additional characterization of BAY-880, Related to Figures 4 and 5.

A. Immunoblots showing pS2 or pS5 phosphorylation of RNAPII upon 24 h treatment with 10 µM BAY-880, NUAK1i (a compound structurally related to BAY-880), WZ4003, HTH-01-015, BX795, 1 µM flavopiridol (FP), 2 µM P276-00, 20 µM LDC000067, 50 µM DRB. VCL was used as loading control (n=3).

B. Venn diagram showing the overlap between direct target sites of CDK9 (n=374) and significantly down-regulated phosphopeptides identified in response to 2 h treatment with 10 µM BAY-880 (n=1020) in a TMT phosphoproteomic experiment.

C. PNUTS chromatin immunoprecipitation (ChIP). Where indicated, cells were treated for 4 h 10 µM BAY-880. IgG was used as antibody specificity control. TSS, transcription start site; 3'RT, 3' readthrough site; neg ct, negative control (mean±SD of technical triplicates of a representative experiment, n=3).

D. Immunoblots documenting phosphorylation of PNUTS or SF3B1 at the indicated sites after treatment of U2OS cells with 10 µM BAY-880, 10 µM HTH-01-015 or 1 µM pladienolide B (PlaB) for the indicated times. VCL was used as loading control (n=3).

E. Layout of nascent RNA-sequencing experiments.

F. Genome browser tracks of nascent RNA expression of *MAFK*, a gene representing the gene set "Transcription from RNAPII promoter" described in Figure 5D. Tracks were first normalized to overall reads, then exonic reads were electronically removed. Cumulative gene browser picture from 3 independent replicates are shown.

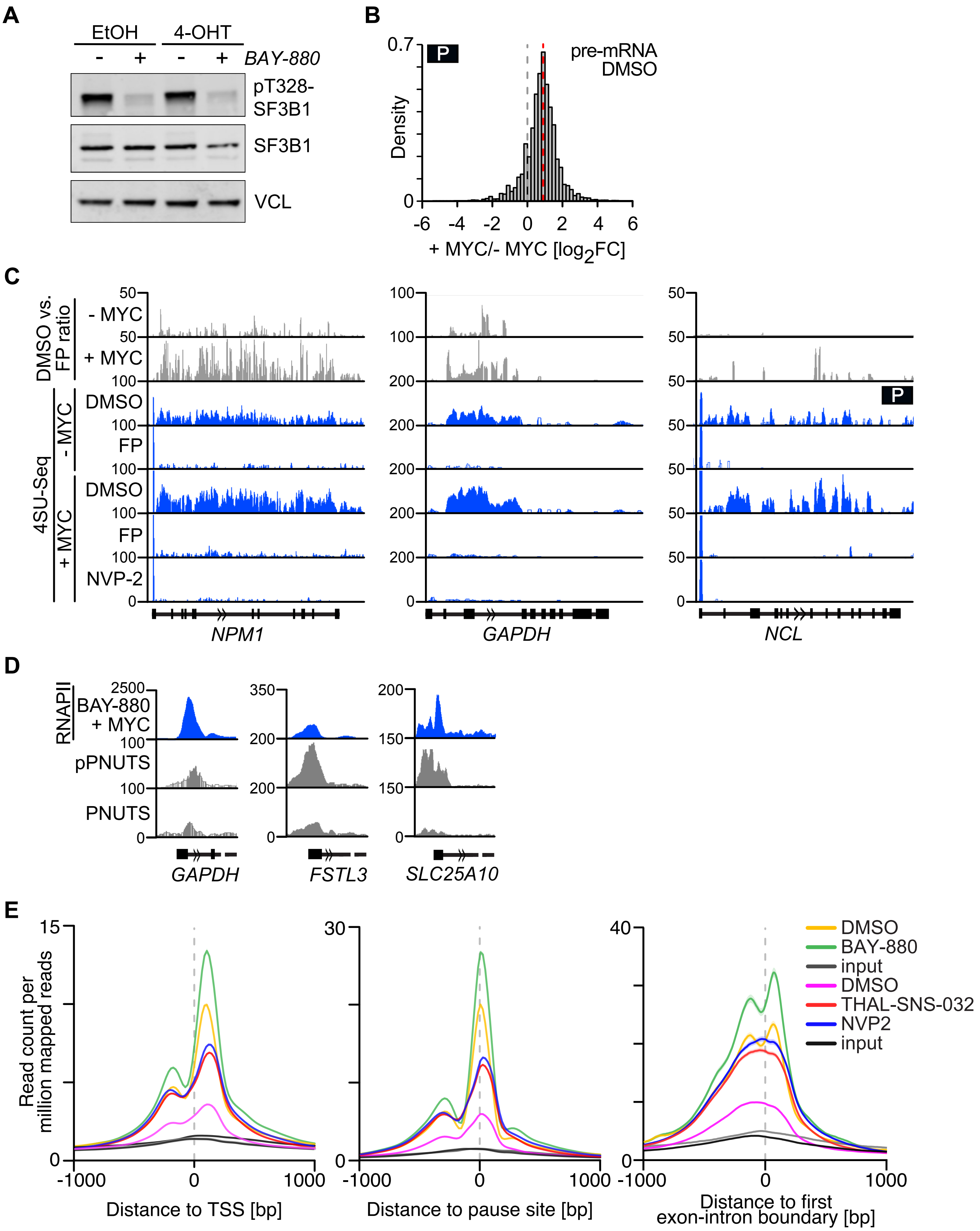
A
EtOH
4-OHT
- + - + BAY-880
pT328-SF3B1
SF3B1
VCL
B
P
pre-mRNA DMSO
Density
+ MYC/- MYC [log2FC]
C
DMSO vs. FP ratio
- MYC
+ MYC
4SU-Seq
DMSO
FP
NVP-2
NPM1
GAPDH
NCL
D
RNAPII BAY-880 + MYC
pPNUTS
PNUTS
GAPDH
FSTL3
SLC25A10
E
Read count per million mapped reads
Distance to TSS [bp]
Distance to pause site [bp]
Distance to first exon-intron boundary [bp]
DMSO
BAY-880
input
DMSO
THAL-SNS-032
NVP2
input

Figure S6. NUAK1 affects nascent RNA synthesis in a MYC-dependent manner, Related to Figures 6 and 7.

A. Immunoblot using the indicated antibodies. U2OS MYC-ER cells were incubated with 100 nM 4-OHT for 20 h and treated with 10 µM BAY-880 for the last 4 h. VCL was used as loading control (n=3).

B. Histogram representing the ratio of pre-mRNA reads (defined in Figure 5A) between 4-OHT treated ("+MYC") and EtOH treated cells ("- MYC") of all genes in the 4sU-labeling experiment. Samples upon 15 min 4sU incorporation ("pulse", "P") are shown.

C. Blue, browser tracks documenting nascent RNA synthesis at the indicated genes as determined by a 15 min pulse ("P") of 4sU incorporation. Where indicated cells, were treated with 1 µM flavopiridol (FP), 1 µM NVP-2 or DMSO for 2 h in control cells ("- MYC") or upon MYC-ER activation ("+ MYC"; 20 h). Grey, ratio of reads in DMSO and FP-treated samples.

D. Blue, RNAPII ChiP-RX genome browser tracks of representative genes upon 4 h 10 µM BAY-880 in MYC activated cells ("+ MYC"). Grey, phospho-S313-PNUTS ("pPNUTS") ChIP-RX or PNUTS ChIP-Seq occupancy.

E. Read density plots from Figure 6B of RNAPII ChIP-RX analysis upon 20 h MYC-ER activation with 100 nM 4-OHT and treatment with 4 h 10 µM BAY-880 or DMSO in U2OS cells compared to RNAPII ChIP-Seq upon CDK9 inhibition by NVP-2 or THAL-SNS-032 (GEO:GSE89384). Plots are centered to transcription start site (TSS, left), RNAPII pause site (middle) or first exon-intron boundary (right). The shadow around tracks indicates SEM.

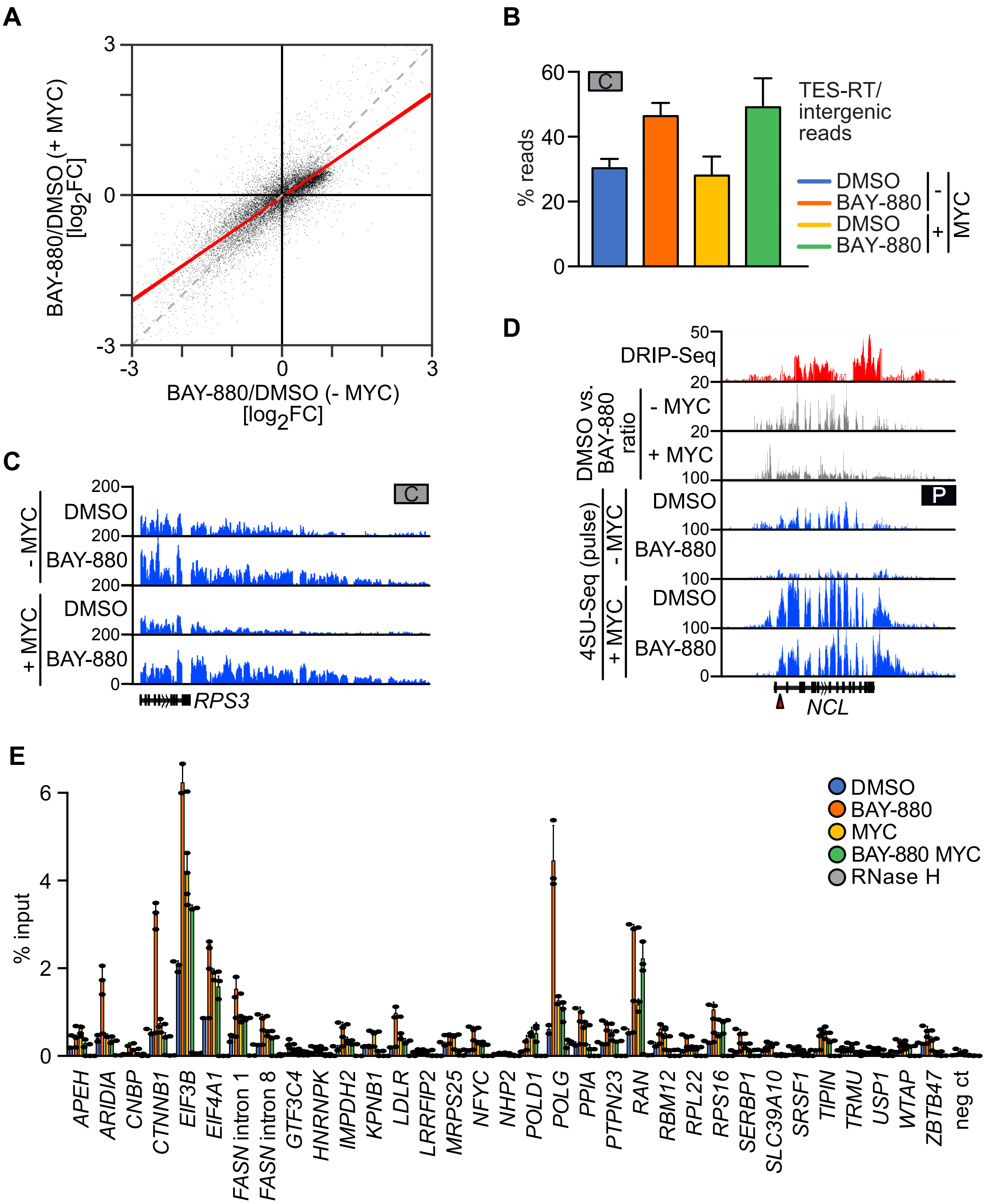

A
BAY-880/DMSO (+ MYC) [log2FC]
BAY-880/DMSO (- MYC) [log2FC]
B
% reads
TES-RT/ intergenic reads
DMSO
BAY-880
DMSO
BAY-880
MYC
C
C
- MYC
+ MYC
DMSO
BAY-880
RPS3
D
DRIP-Seq
DMSO vs. BAY-880 ratio
- MYC
+ MYC
4SU-Seq (pulse)
P
NCL
E
% input
DMSO
BAY-880
MYC
BAY-880 MYC
RNase H
APEH
ARIDIA
CNBP
CTNNB1
EIF3B
EIF4A1
FASN intron 1
FASN intron 8
GTF3C4
HNRNPK
IMPDH2
KPNB1
LDLR
LRRFIP2
MRPS25
NFYC
NHP2
POLD1
POLG
PPIA
PTPN23
RAN
RBM12
RPL22
RPS16
SERBP1
SLC39A10
SRSF1
TIPIN
TRMU
USP1
WTAP
ZBTB47
neg ct

Figure S7, Related to Figure 7. NUAK1 affects R-loop formation and decapping enzyme recruitment in a MYC-dependent manner.

A. Scatter plot comparing change in expression of all expressed genes (n=11,891) in response to BAY-880 treatment in control cells ("- MYC") and MYC-expressing cells ("+ MYC"). Each black dot represents a single gene. Red line indicates linear regression curve for the overall density (note the difference to the diagonal, grey dashed line).

B. Bar plot depicting percentage (mean±SD, n=3) of "TES-RT" and intergenic reads (defined in Figure 5A) in the 4sU-labeling experiment, representing defective termination. Chase samples ("C") are shown.

C. Genome browser tracks of nascent RNA expression of *RPS3*, a representative gene displaying termination readthrough. Chase samples ("C") are shown.

D. Red, DRIP(DNA-RNA-Immunoprecipitation)-Seq data (GEO:GSE115957) of the *NCL* gene in U2OS cells. Blue, genome browser tracks of nascent RNA synthesis (15 min 4sU incorporation, "pulse") of *NCL* upon treatment with 4 h 10 µM BAY-880 or DMSO in control cells ("- MYC") or upon MYC activation ("+ MYC"). Grey, read ratio between DMSO and BAY-880 samples. The red arrow indicates the position of primers used for DRIP-qPCR (Figure 7D, S7H).

E. DRIP-qPCRs (using S9.6 antibody) of U2OS MYC-ER cells treated with DMSO or 10 µM BAY-880 for 4 h and, where indicated, co-treated with 100 nM 4-OHT for 20 h ("MYC"). RNase H treatment and a negative region were employed to test antibody specificity (mean±SD of technical triplicates of a representative experiment, n=3).

kinase	%	kinase	%	kinase	%	kinase	%
NUAK1	4	FLT1	53	ALK2	76	IGF1R	87
CDK2	4	GCK	53	EPHB2	76	MST1	87
CDK3	4	TBK1	54	FGFR2	76	TAO2	87
CDK9	4	CSK	55	JAK1	77	WNK2	87
CDK4	9	MST2	55	MSSK1	77	DYRK1B	88
AURKB	15	CAMK2D	56	AXL	78	FGFR3	88
CLK1	16	MST3	56	EPHA4	79	P70S6K	88
CLK4	17	PTK5	56	LKB1	79	PRAK	88
FLT3	17	LRRK2	57	RSK1	79	WEE1	88
AURKC	19	DRAK1	58	TAK1	79	CK2A2	89
TYK2	20	HCK	58	BTK	80	HASPIN	89
CDK5	22	LCK	58	FAK	80	TLK1	89
SRC	22	NEK9	60	LIMK1	80	CHK2	90
MLK1	27	FES	61	MNK2	80	CLK3	90
CDK1	28	LOK	61	PIM1	80	IKKB	90
IRAK1	30	DDR1	62	RIPK2	80	TIE2	90
EPHA1	33	GSK3A	62	BRSK1	81	TSSK2	90
MER	34	ALK	63	DYRK1A	81	EPHA3	91
CDK6	35	EPHA2	63	TAO3	81	GCN2	91
YES	35	IR	63	PKCA	82	MRCKB	91
LYN	36	IRR	65	CRAF	83	PLK3	91
AMPKA2	37	ARG	66	MAPK2	83	TXK	91
FLT4	39	ITK	66	MELK	83	ATM	91
SIK	40	BLK	67	NIM1	83	DMPK	92
CAMK2G	43	CDK7	67	PKCZ	83	EPHB4	92
FYN	43	BRSK2	69	WNK3	83	PKCE	92
AMPKA1	44	CAMKK2	69	CAMK2B	84	ULK1	92
STK33	44	KDR	70	KIT	84	VRK2	92
CLK2	50	PYK2	70	MSK2	84	PIP4K2A	92
GSK3B	50	TAO1	70	PKBA	84	ALK4	93
Rsk3	50	ABL	72	NLK	85	PKCM	93
TEC	50	CHK1	72	TLK2	85	PI3K	93
ACK1	51	FGR	72	EPHA5	86	LTK	94
BMX	51	IKKE	72	JAK2	86	MKK7B	94
EPHB1	52	RSK2	72	SAPK3	86	PKBG	94
FGFR1	52	PDGFRA	73	ALK1	87	PKCI	94
MET	52	ULK3	74	DDR2	87	SGK3	94
AURKA	53	NEK3	75	ERBB4	87	SNK	94

kinase	%	kinase	%	kinase	%	kinase	%
TSSK1	94	CK1	99	ALK6	104	PKR	108
ATR	94	DAPK1	99	FMS	104	PLK1	108
ARAF	95	MAPK1	99	JNK2A2	104	ROCK1	108
GRK1	95	MUSK	99	NEK11	104	ZAP-70	108
MARK1	95	PKBB	99	PKCBI	104	EEF2K	109
PEK	95	ROCKII	99	PKCG	104	GRK5	109
PIM3	95	ChAK1	100	PRKX	104	MINK	109
PKCD	95	CK1D	100	RET	104	CAMKIV	110
TRKA	95	DYRK2	100	ASK1	105	PIP5K1G	110
PI3KC2	95	FER	100	DAPK2	105	HIPK2	111
BRAF	96	HIPK3	100	MRCKA	105	SAPK4	111
GRK7	96	IKKA	100	RSK4	105	PIM2	112
MAPKAPK2	96	MKK6	100	SGK2	105	SRPK2	112
PKA	96	ULK2	100	TGFBR1	105	SYK	112
PKCBII	96	DNAPK	100	CAMK1D	106	ZIPK	112
PKCT	96	SAPK2A	101	CK2	106	CK1G2	113
PI3KC2A	96	SAPK2B	101	EPHA8	106	NEK7	113
JAK3	97	SRPK1	101	IRAK4	106	PHKG2	113
JNK1A1	97	STK25	101	MST4	106	NEK2	114
PAK5	97	BRK	102	PAK2	106	TRKB	114
PAK6	97	EGFR	102	PAK4	106	DCAMKL3	115
PAR1BA	97	PKG1A	102	CK1G1	107	ERBB2	115
PKCN	97	FGFR4	103	GRK6	107	EPHB3	117
EPHA7	98	MEK1	103	PAK1	107	SGK	118
MAPKAPK3	98	MKK4	103	PKG1B	107	RON	120
PASK	98	MLCK	103	RSE	107	CK1G3	124
PDK1	98	MOK	103	HIPK1	108	DCAMKL2	124
PKD2	98	FKBP12	103	IRE1	108	JNK3	127
PRK2	98	PDGFRB	103	MSK1	108	SNRK	128
TRKC	98	ROS	103	MTOR	108		
CAMKI	99	PIP5K1A	103	NEK6	108		

Table S1. BAY-880 selectivity screening, Related to Figure 4

The kinase selectivity screening was performed employing the KinaseProfiler Assay (Eurofins). %, percentual residual kinase activity upon 1uM BAY-880 treatment.

Gene (region)	Forward primer	Reverse primer
ACTG1 (TSS)	CGCTCACCGGCAGAGAAA	CGGTCGGTCTCAGTCGC
ARGHDIA (3'RT)	CACACCAAGCCTTTTCGCTG	CTGAGGCAGGAAGTAGGTGC
CYR61 (3'RT)	ATGAGCTTGAAGCATGACTTGTG	TTGGCCCTCACGCTATTGG
CYR61 (TSS)	GGCCCGTATAAAAGGCGGG	GCGTCTTTCGCTCGAGGTC
FASN (TSS)	CGTCTCTCTGGCTCCCTCTA	GCCAAGCTGTCAGCCCAT
FASN (intron 1)	CTGGTCTGGCCACTTGCAC	ACCCCGCGTGAATAGCAA
HIST2H3C (TSS)	TCTGGTAGCGCCGGATCTC	CAAGGCCCCGAGGAAGC
HIST2H3C (3'RT)	GGAGCCGGACCGCCAAA	CAATTGGCCTATCCGCACTGG
FSTL3 (TSS)	CGTCTCTGCGTTCGCCAT	CATGGAGCTCACGAAGCCC
LDHA (TSS)	GGATCTCATTGCCACGCG	CTCTACCCGCCCATCCCT
ACTB (TSS)	CGGGGTCTTTGTCTGAGC	CAGTTAGCGCCCAAAGGAC
LDLR (TSS)	AGACTTGTGGGGTAATGGCA	AAAGAAGATGCGGTCCCTCA
Negative control	TTTTCTCACATTGCCCCTGT	TCAATGCTGTACCAGGCAAA

Table S2. Primers for ChIP, Related to STAR Methods

Primers employed for ChIP-qPCR of the respective genes.

TSS, transcription start site; 3'RT, 3‘ readthrough.

Primer name	Aim	Sequence
NUAK1 fwd	FLAG/HA-NUAK1 cloning	GATCGGATCCATGGAAGGGGCGGCAGT
NUAK1 rev	FLAG/HA-NUAK1 cloning	GATCGAATTCCTAGTTGAGCTTGCTGCAGATCTC
NTD-FLAG-NUAK1 fwd	FLAG/HA-NUAK1 cloning	GATCGGATCCATGTGGTCGCATCCGCAGTTCGAGAAGGATTACAAGGATGACGATGACAAGAGTGCAGAAGGGGCGGCAGTGTC
CTD-FLAG-NUAK1 rev	FLAG/HA-NUAK1 cloning	GATCGAATTCCTACTTCTCGAACTGCGGATGCGACCACTTGTCATCGTCATCCTTGTAATCTGCACTGTTGAGCTTGCTGCAGATCTCC
NTD-HA-NUAK1 fwd	FLAG/HA-NUAK1 cloning	GATCGGATCCATGTACCCATACGATGTTCCAGATTACGCTGAAGGGGCGGCAGTGT
CTD-HA-NUAK1 rev	FLAG/HA-NUAK1 cloning	GATCGAATTCCTAAGCGTAATCTGGAACATCGTATGGGTACGTTGAGCTTGCTGCAGATC
PNUTS_fwd	HA-PNUTS cloning	GATCGGATCCATGGGTTCAGGTCCCATAGACCCC
PNUTS_rev	HA-PNUTS cloning	GATCGAATTCCTAGGGCAGTGGGGGCCC
NTD-HA-PNUTS for	HA-PNUTS cloning	GATCGGATCCATGTACCCATACGATGTTCCAGATTACGCTGCATCGGGTTCAGGTCCCATAGACCCC
CTD-HA-PNUTS rev	HA-PNUTS cloning	GATCGAATTCCTAAGCGTAATCTGGAACATCGTATGGGTATGCACTGGGCAGTGGGGGCCC
S313A-PNUTS sense	PNUTS mutagenesis	AGAAGAAGGTACTATCGCCGACTGCTGCCAA
S313A-PNUTS antisense	PNUTS mutagenesis	TTGGCAGCAGTCGGCGCTAGTACCTTCTTCT
S313D-PNUTS sense	PNUTS mutagenesis	AGAAGAAGGTACTAGATCCGACTGCTGCCAA
S313D-PNUTS antisense	PNUTS mutagenesis	TTGGCAGCAGTCGGATCTAGTACCTTCTTCT
S313E-PNUTS sense	PNUTS mutagenesis	AGAAGAAGGTACTAGAGCCGACTGCTGCCAA
S313E-PNUTS antisense	PNUTS mutagenesis	TTGGCAGCAGTCGGCTCTAGTACCTTCTTCT

Table S3. Primers for cloning, Related to STAR Methods

Primers employed for the reported cloning aim.

Gene (region*)	Forward primer	Reverse primer
ACTB (intron 1)	CGGGGTCTTTGTCTGAGC	CAGTTAGCGCCCAAAGGAC
ACTB (intron 3)	TAACACTGGCTCGTGTGACAA	AAGTGCAAAGAACACGGCTAA
APEH	CTCATTGGTCCCATTCCCCT	CTGAGTCCTGGGCCTCTTAC
ARIDIA	TTGGAGACTGGGGCTACTTG	AAGCTGCCTTCGGTCTACTT
CNBP	CGACTATACCCACCCCATCC	TCTTCGTCCTGGAAAGCTGG
CSRNP1	TATAGCTCCCCTTGGGGCAT	GGACATGGCCATTACGGGAA
CTNNB1	TGACTCAGACCGCTTCGAGA	TCCATTTGGCCAGCTTTGGA
EIF3B	TGGGTGTGCTGTGAGTGTAG	ATGGACAATTCTGAGGGGCA
EIF4A1	ACGTGTGAGAGTGCAGGG	TTAGTTCCTAGTCGCTCCGG
FASN (intron 1)	CTGGTCTGGCCACTTGCAC	ACCCCGCGTGAATAGCAA
FASN (intron 8)	CACTTCCTGTCCCCAACCTA	CCAACACCCATGATCACTCA
GTF3C4	GTCCTGGGGTTGTCGAAGA	GTAGTCTTGTCCCTACCCGC
HNRNPK	CTAGTCCCCAGGCCTCAAAA	CTCCGCCTAGTAGCACGTAG
IMPDH2	CAGTTGAAGAGCTGCTGTGC	TGGTTTATATTGGCGCGGC
KPNB1	ACCCCGAACTCCTCTCCTTA	TTGGGATGGGGTAGGAGAGA
LDLR	AGACTTGTGGGGTAATGGCA	AAAGAAGATGCGGTCCCTCA
LRRFIP2	CAGCTAGCGCCTGTTAAACC	TAGACGCCACACTACGGTTC
MRPS25	GCGCGAGAAGCATTCAGAAC	GACACCCCAGGACGAATCTG
NCL	CTACCACCCTCATCTGAATCC	TTGTCTCGCTGGGAAAGG
NFYC	AATACACACACACGCCCCTA	GGAGGCGGAATGAGATCTGA
NHP2	AGGCATCACTTCCAGGTCAT	CCCTTGCCTCACATTCCCT
POLD1	GGAGGCGGAGTTAAGGGAAT	CCTCTACTCACCCGCTTCAA
POLG	CTTCTCAAGGAGCAGGTGGA	TCATAACCTCCCTTCGACCG
PPIA	CCTTGAGAGTCGTTGGGCT	CATGGACGGGCTCACACC
PTPN23	CCAGTCTCCGGTCAGTGATT	CGTATTGTCAAGAGCCGTGG
RAN	CCGTGACTCTGGGATCTTGA	CAAGGTGGCTGAAACGGAAA
RBM12	GAGTCTTACCGGGGAAAGCT	CATTTTGTGAAGCGGCGAAG
RPL22	GCGGAGTTAGAAAGGGAGGT	TTCCTTCCCCAGAAACCCTC
RPS16	CCGAGCGTGGACTAGACAA	GTTAGCCGCAACAGAAGCC
SERBP1	ACTACAATTCCCAGGACGCA	GCACTTTCGCGAGTCAGTTA
SLC39A10	CGAATGATAAAGGGCGCTCC	CCAGTTGCGTAAAGGAGTCG
SRSF1	GGAAACAGCGATTCGATCCC	CTGGTCACTCTGTTCGCAAA
TIPIN	TCACCTCACGCAGAAAACAC	CAGTAGGGCGGAAATTGTGG
TRMU	TGTCCCCGGAAACCTGTC	CACACCTCCGACTACACAGG
USP1	GTGCCTGCGTTGTTTGAAAC	CGCGTTTTCCTCAGTCTCAG
WTAP	GCGGAGTTAGAAAGGGAGGT	TTCCTTCCCCAGAAACCCTC
ZBTB47	TGCAGGTACAGGATGTCTGC	GCCAGAATACGTAGGGCCTC
Negative control	TTTTCTCACATTGCCCCTGT	TCAATGCTGTACCAGGCAAA

Table S4. Primers for DRIP, Related to STAR Methods

Primers employed for DRIP-qPCR of the respective genes. If not elsewhere stated, every primer pair targets the transcription start site of the gene.
