# Supplementary material for: Localized Inhibition of Protein Phosphatase 1 by NUAK1 Promotes Spliceosome Activity and Reveals a MYC-Sensitive Feedback Control of Transcription
Source: Mol Cell. 2020 Mar 19;77(6):1322–1339.e11. doi: 10.1016/j.molcel.2020.01.008 (PMC7086158; doi:10.1016/j.molcel.2020.01.008)
Supplement: Document S2. Article plus Supplemental Information [file mmc2.pdf]

Molecular Cell

Article

Localized Inhibition of Protein Phosphatase 1 by NUAK1 Promotes Spliceosome Activity and Reveals a MYC-Sensitive Feedback Control of Transcription

Graphical Abstract

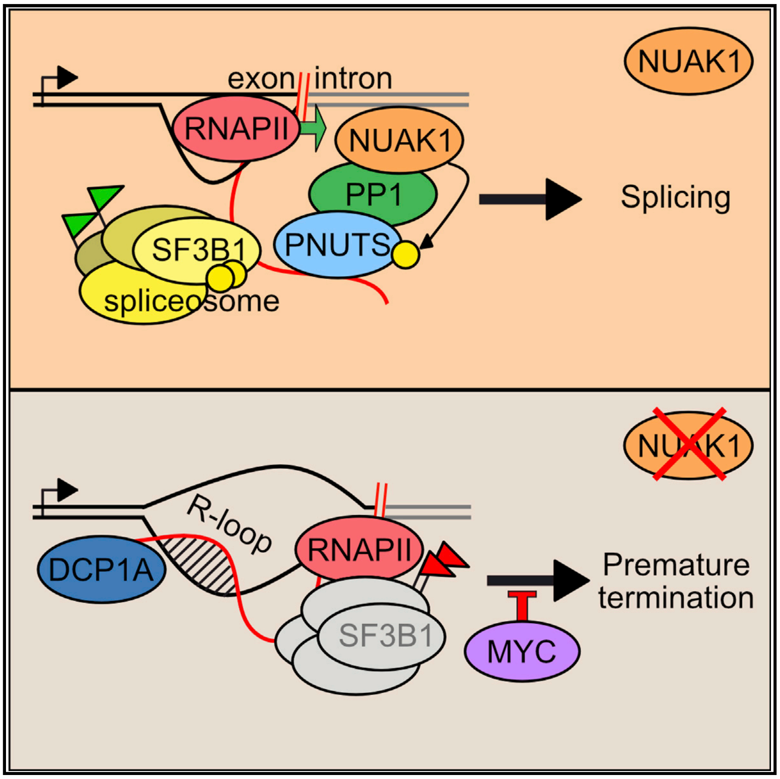

Highlights

- Nuclear NUAK1 associates with PP1 and phosphorylates its targeting subunit PNUTS
- NUAK1, PP1, and PNUTS form a trimer that associates with the splicing machinery
- Inhibition of NUAK1 reduces spliceosome activity and nascent RNA synthesis
- When MYC is deregulated, NUAK1 inhibition traps RNAPII at the intron-exon boundary

Authors

Giacomo Cossa, Isabelle Roeschert, Florian Prinz, ..., Elmar Wolf, Dominik Mumberg, Martin Eilers

Correspondence

martin.eilers@biozentrum.uni-wuerzburg.de

In Brief

Tumors displaying high MYC levels depend on the NUAK1 kinase. Cossa et al. show that NUAK1 binds protein phosphatase 1 (PP1) and its regulatory subunit PNUTS. They show that this complex associates with the splicing machinery and provides a feedback control of transcription that can be overridden by deregulated MYC.

Cossa et al., 2020, Molecular Cell 77, 1322–1339
March 19, 2020 © 2020 The Authors. Published by Elsevier Inc.
https://doi.org/10.1016/j.molcel.2020.01.008

Localized Inhibition of Protein Phosphatase 1 by NUAK1 Promotes Spliceosome Activity and Reveals a MYC-Sensitive Feedback Control of Transcription

Giacomo Cossa,[1] Isabelle Roeschert,[1] Florian Prinz,[2] Apoorva Baluapuri,[3] Raphael Silveira Vidal,[1] Christina Schülein-Völk,[1] Yun-Chien Chang,[4] Carsten Patrick Ade,[1] Guido Mastrobuoni,[5] Cyrille Girard,[6] Lars Wortmann,[2] Susanne Walz,[7] Reinhard Lührmann,[6] Stefan Kempa,[5] Bernhard Kuster,[4] Elmar Wolf,[3] Dominik Mumberg,[2] and Martin Eilers[1,8,*]
[1]Department of Biochemistry and Molecular Biology, Biocenter, University of Würzburg, Am Hubland, 97074 Würzburg, Germany
[2]Lab GBM Research 5, Research & Development, Pharmaceuticals, Bayer AG, Building S155, 13342 Berlin, Germany
[3]Cancer Systems Biology Group, Biocenter, University of Würzburg, Am Hubland, 97074 Würzburg, Germany
[4]Technical University of Munich, Emil-Erlenmeyer Forum 5, 85354 Freising, Germany
[5]Berlin Institute for Medical Systems Biology at The Max-Delbrück-Center for Molecular Medicine, Robert-Rössle-Str. 10, 13125 Berlin, Germany
[6]Department of Cellular Biochemistry, Max-Planck Institute for Biophysical Chemistry, Am Fassberg 11, 37077 Göttingen, Germany
[7]Comprehensive Cancer Center Mainfranken, Core Unit Bioinformatics, Biocenter, University of Würzburg, Am Hubland, 97074 Würzburg, Germany
[8]Lead Contact
*Correspondence: martin.eilers@biozentrum.uni-wuerzburg.de
https://doi.org/10.1016/j.molcel.2020.01.008

SUMMARY

Deregulated expression of MYC induces a dependence on the NUAK1 kinase, but the molecular mechanisms underlying this dependence have not been fully clarified. Here, we show that NUAK1 is a predominantly nuclear protein that associates with a network of nuclear protein phosphatase 1 (PP1) interactors and that PNUTS, a nuclear regulatory subunit of PP1, is phosphorylated by NUAK1. Both NUAK1 and PNUTS associate with the splicing machinery. Inhibition of NUAK1 abolishes chromatin association of PNUTS, reduces spliceosome activity, and suppresses nascent RNA synthesis. Activation of MYC does not bypass the requirement for NUAK1 for spliceosome activity but significantly attenuates transcription inhibition. Consequently, NUAK1 inhibition in MYC-transformed cells induces global accumulation of RNAPII both at the pause site and at the first exon-intron boundary but does not increase mRNA synthesis. We suggest that NUAK1 inhibition in the presence of deregulated MYC traps non-productive RNAPII because of the absence of correctly assembled spliceosomes.

INTRODUCTION

The MYC oncoprotein is a transcription factor that regulates broad programs of gene expression, promoting cell proliferation and cell growth and inducing major changes in growth-associated processes such as cellular metabolism and the interaction of cells with the micro-environment (Dang, 2012; Kress et al., 2015). MYC proteins are almost universally present at active core promoters. Proteomic analyses show that MYC and its paralog MYCN affect the function of RNA polymerase II (RNAPII) via multiple distinct protein complexes (Baluapuri et al., 2019; Büchel et al., 2017; Kalkat et al., 2018). MYC proteins can enhance recruitment of RNAPII to promoters (de Pretis et al., 2017), promoter escape (Büchel et al., 2017), release of RNAPII from the pause site (Rahl et al., 2010; Walz et al., 2014), and RNAPII processivity during elongation (Baluapuri et al., 2019). MYCN can suppress the accumulation of promoter-proximal R-loops and the recruitment of mRNA de-capping complexes, which terminate transcription at the pause site (Brannan et al., 2012; Herold et al., 2019).

Cells and tumors expressing deregulated levels of MYC rely on a number of specific factors for survival, including an enhanced dependence on anti-apoptotic proteins and trophic signals (Pelengaris et al., 2002), on glutamine as a nutrient source (Gao et al., 2009; Xiang et al., 2015), on splicing factors (Hsu et al., 2015), on cyclin-dependent kinases (Chipumuro et al., 2014; Christensen et al., 2014; Huang et al., 2014), and on AMP-dependent kinase (AMPK), which is activated by an increase in cellular AMP levels (Kfoury et al., 2018; Liu et al., 2012). The analysis of these dependencies has produced critical insights into the process of MYC-driven oncogenic transformation and led to new approaches to selectively eradicate MYC-driven tumor cells for therapy (Dang, 2016; Haikala et al., 2019). Cells expressing deregulated levels of MYC also depend on the AMPK-related kinase NUAK1 (also known as ARK5, AMPK-related kinase 5) (Liu et al., 2012; Monteverde et al., 2018). Likewise, colon tumors, which express high MYC levels because of loss-of-function mutations in the APC tumor suppressor gene, depend on NUAK1 for tumor growth and maintenance (Port et al., 2018). Several explanations have been put

 © 2020 The Authors. Published by Elsevier Inc.
This is an open access article under the CC BY license (http://creativecommons.org/licenses/by/4.0/).

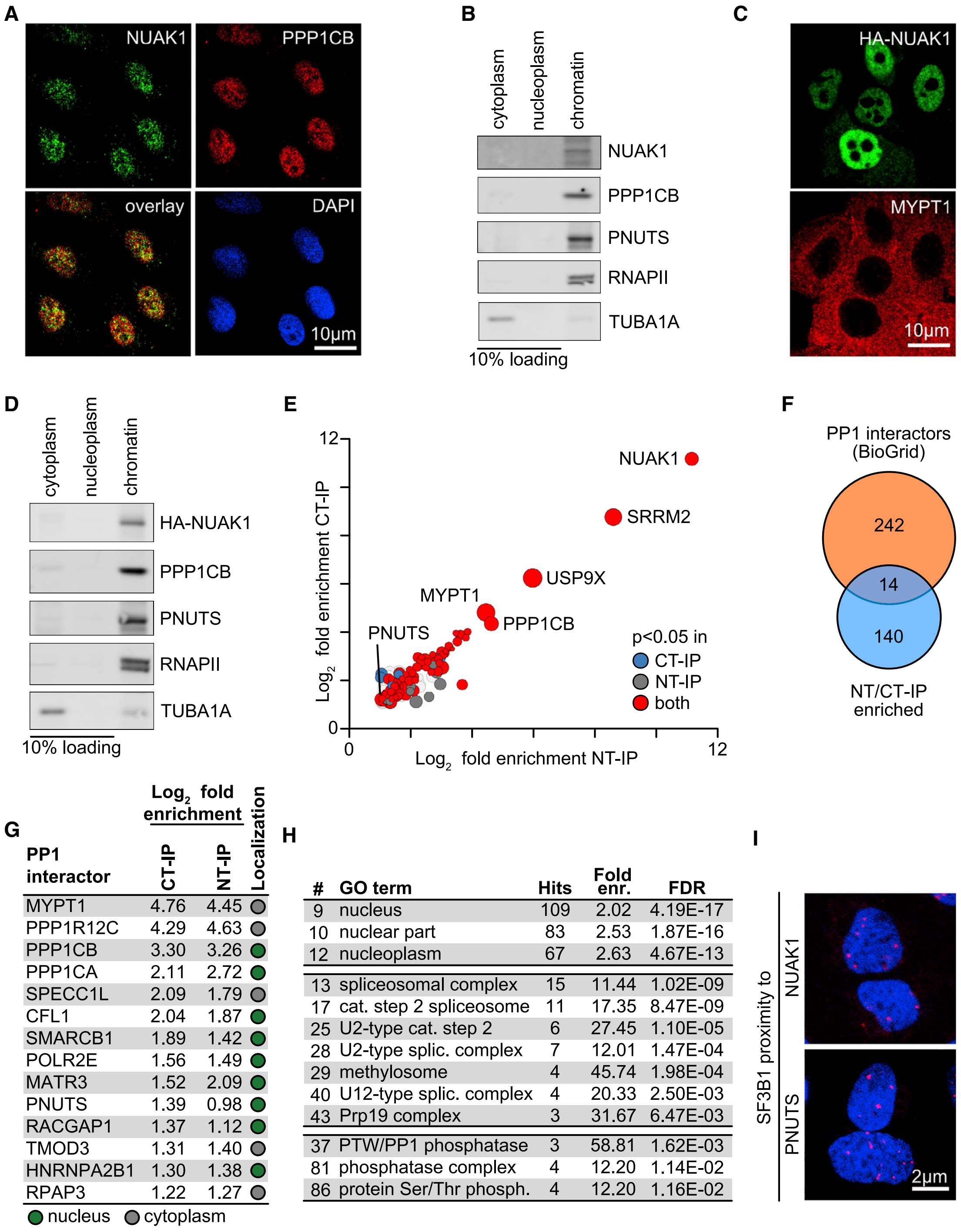

Figure 1. NUAK1 Binds to Chromatin and Interacts with a Nuclear PP1 Network

(A) U2OS cells stained for endogenous NUAK1 and PPP1CB. DAPI is used as nuclear counterstain (n = 3; in all legends, n indicates the number of independent biological replicates).

(B) Immunoblot of fractionation of U2OS cells probed with the indicated antibodies. Ten percent of cytoplasm and nucleoplasm fractions were loaded. RNAPII (chromatin) and TUBA1A (cytoplasm) were used as localization controls (n = 3).

(C) Immunofluorescence of U2OS cells stably expressing HA-tagged NUAK1 stained with α-HA and α-MYPT1 antibodies (n = 3).

(D) Cell fractionation of U2OS cells stably expressing HA-tagged NUAK1. Ten percent of cytoplasm and nucleoplasm fractions were loaded. RNAPII and TUBA1A were used as controls (n = 3).

(legend continued on next page)

forward to explain this dependence; for example, NUAK1 has been linked to cellular energy metabolism (Liu et al., 2012), to p53 function (Hou et al., 2011), and to responses to oxidative stress (Port et al., 2018), which affect the nuclear localization of NUAK1 (Palma et al., 2019). Nevertheless, the biochemical processes that establish the dependence of MYC-overexpressing cells on NUAK1 are not as clear as for the other dependencies described above.

A well-established function of NUAK1 is to control the activity of protein phosphatase 1 (PP1) (Zagórska et al., 2010). PP1 holoenzymes consist of a broadly active catalytic core (encoded by one of three highly homologous genes: *PPP1CA*, *PPP1CB*, or *PPP1CC*) and one of many regulatory subunits (Verbinnen et al., 2017). Regulatory subunits can both target the PP1 holoenzyme to specific compartments in a cell and control its catalytic activity (e.g., some subunits inhibit PP1 activity toward specific substrates) (Verbinnen et al., 2017). In the cytoplasm, a major regulatory subunit of PP1 is MYPT1 (myosin phosphatase target subunit 1, encoded by the gene *PPP1R12A*), which regulates the interaction of actin and myosin (Matsumura and Hartshorne, 2008). NUAK1 directly interacts with PPP1CB and forms a trimeric complex with PPP1CB and MYPT1. In the trimer, NUAK1 phosphorylates MYPT1 and promotes association of MYPT1 with 14-3-3 proteins (Zagórska et al., 2010). This reaction blocks the interaction of MYPT1 and PP1 with myosin light-chain kinase (MLC2), increases phosphorylation of MLC2, and thereby activates myosin II. The MYPT1/NUAK1 interplay also regulates the activity of Polo-like kinase (PLK1) throughout the cell cycle (Banerjee et al., 2014b; Werle et al., 2014) and controls AKT-dependent phosphorylation of GSK3β (Port et al., 2018). Whether NUAK1 has similar roles on other PP1 holoenzymes in a cell is unknown.

Here we show that NUAK1 complexes with PP1 in the nucleus and promotes spliceosome activity. NUAK1 and PP1 are involved in a regulatory circuit that couples transcriptional elongation to spliceosome activity. Deregulated expression of MYC overrides this control, providing a mechanistic model why tumor cells with high MYC levels depend on NUAK1.

RESULTS

NUAK1 Binds to Chromatin and Interacts with a Nuclear PP1 Network

Previous studies have shown that NUAK1 associates with PP1 complexes and have identified the cytoplasmic PP1 regulatory subunit MYPT1 as a major interaction partner of NUAK1 (Zagórska et al., 2010). Surprisingly, however, endogenous NUAK1 localized mainly to the nucleus of U2OS osteosarcoma cells; the staining occurred in a speckled pattern that partly co-localized with the nuclear pool of the protein phosphatase catalytic subunit beta (PPP1CB) (Figure 1A). NUAK1 was also localized mainly in the nucleus in a panel of additional cell lines that we analyzed (Figure S1A), consistent with a recent study (Palma et al., 2019). Fractionation experiments showed that a significant fraction of both NUAK1 and PPP1CB was bound to chromatin (Figure 1B). To exclude that this staining reflected a cross-reactivity of the antibody, we stably expressed HA-tagged NUAK1 in U2OS and MCF10A cells (HA-NUAK1). Like its endogenous counterpart, the bulk of HA-NUAK1 localized to the cell nucleus and bound to chromatin (Figures 1C, 1D, and S1B). Staining of control cells infected with empty vectors confirmed the specificity of staining (Figure S1B). Parallel immunostaining confirmed that MYPT1 is localized mainly in the cytoplasm of U2OS cells (Figure 1C).

To identify nuclear interaction partners of NUAK1, we immunoprecipitated cell lysates of U2OS cells stably expressing either N-terminally or C-terminally FLAG-tagged NUAK1 with antibodies directed against the FLAG tag. Control immunoprecipitations showed that both amino-terminally and C-terminally tagged NUAK1 efficiently co-precipitated MYPT1 as well as PPP1CB (Figure S1C). Mass spectrometry of recovered complexes revealed that immunoprecipitations using an anti-FLAG antibody from either cell line enriched for a virtually identical set of proteins relative to control immunoprecipitations from empty vector-infected cells (Figure 1E). Gene Ontology (GO) term analyses and comparison with a protein interaction database showed that the immunoprecipitates were significantly enriched for protein phosphatase complexes and contained multiple known interactors of PP1, the majority of which localizes to the nucleus (Figures 1F–1H and S1D). In addition, NUAK1 associated with multiple proteins involved in mRNA splicing (Figure S1D). Specifically, components of the U2 and U12 spliceosomal small nuclear ribonucleoprotein particle (snRNP) complexes, which mediate the recognition of the splicing branching point on pre-mRNA, as well as the Prp19 complex and the methylosome, which are involved in spliceosome assembly, were enriched in the immunoprecipitates (Figure 1H) (Chanarat et al., 2011; Chuang et al., 2011; David et al., 2011). Consistently, immunofluorescence showed that the speckled pattern of NUAK1 co-localized to a significant degree with the spliceosomal protein SC35 (encoded by *SRSF2*), a typical nuclear speckle marker (Girard et al., 2012) (Figure S1E), and proximity ligation assay (PLA) showed that NUAK1 interacts with SF3B1 (Figure 1I). We have recently characterized the RNAPII and MYC interactomes, both of which contain multiple proteins involved in transcription and RNA processing (Baluapuri

(E) Mass spectrometry (MS) analysis of FLAG-NUAK1 co-immunoprecipitates (IP) from U2OS cells expressing amino-(NT-IP)- or carboxy-(CT-IP)-terminally-FLAG-tagged NUAK1. Proteins are sorted according to log_2 fold enrichment over IP performed in U2OS cells expressing empty vector (EV). Dot size is according to number of peptides identified by MS (n = 2).
(F) Venn diagram of NUAK1 interactors and previously documented PP1 interaction partners (BioGrid database).
(G) List of the 14 PP1 interactors from (F). Nuclear (green) or cytoplasmic (gray) localization is shown.
(H) List of selected GO terms enriched by analyzing proteins enriched in both NT- and CT-IP (n = 154). Terms referring to nuclear protein, splicing factors, or PP1 interactors are shown. FDR, false discovery rate; fold enr., fold enrichment.
(I) Proximity ligation assay (PLA) in U2OS cells documenting proximity of SF3B1 and NUAK1 or PNUTS. Red dots indicate proximity of the indicated proteins. DAPI is used as nuclear counterstain.
See also Figure S1.

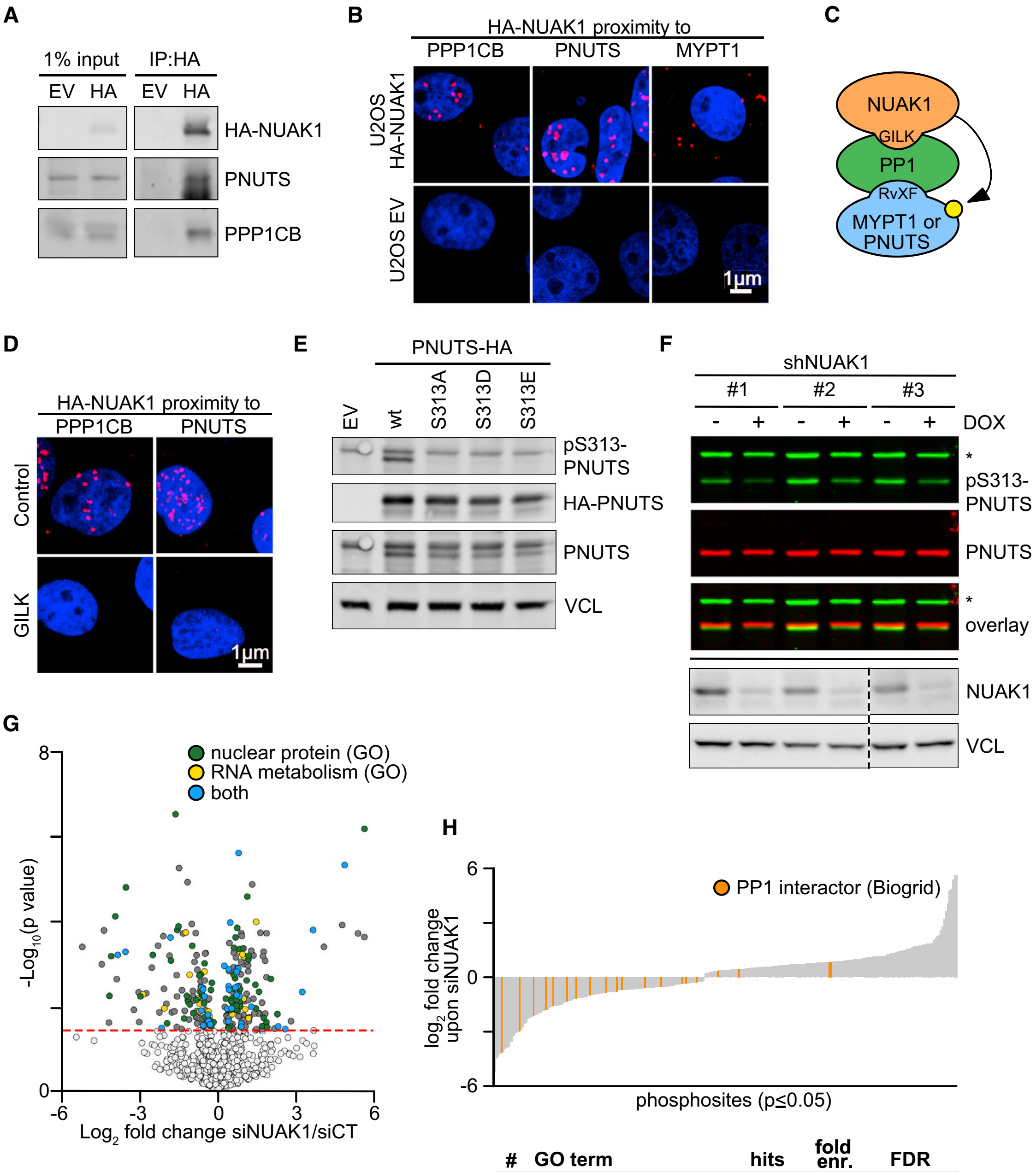

I

#	GO term	hits	fold enr.	FDR
5	nuclear part	101	2.39	8.47E-17
8	nucleus	130	1.87	5.87E-15
11	nuclear lumen	91	2.36	7.48E-14
13	nucleoplasm	81	2.47	1.04E-12

#	GO term	hits	fold enr.	FDR
9	reg. mRNA metab. proc.	17	5.57	4.08E-05
14	RNA splicing	17	4.63	3.30E-04
16	mRNA metab. process	22	3.45	6.77E-04
22	nucleobase-containing metabolic process	51	1.96	1.56E-03
24	mRNA processing	17	3.85	2.23E-03

Figure 2. PNUTS Interacts with and Is Phosphorylated by NUAK1 in the Nucleus

(A) Immunoblot of α-HA immunoprecipitates of U2OS cells expressing HA-tagged NUAK1 or empty vector (EV). Input corresponds to 1% lysate (n = 3).

(B) Proximity ligation assay (PLA) performed in U2OS cells expressing HA-tagged NUAK1 or EV (used as negative control). Red dots indicate proximity of the indicated proteins. DAPI is used as nuclear counterstain (n = 3).

(C) Cartoon depicting the mode of interaction of NUAK1 with MYPT1 and the suggested mode of interaction with PNUTS. Binding motifs of NUAK1 (GILK) and MYPT1/PNUTS (RVxF) to PP1 are also depicted. Yellow circle, phosphorylation.

(D) PLA performed in U2OS cells expressing HA-tagged NUAK1 or EV (used as negative control). Cells were treated for 3 h with 50 μM GILK or control peptide. Red dots indicate proximity of the indicated proteins. DAPI is used as nuclear counterstain (n = 3).

(legend continued on next page)

et al., 2019). The overlap of the NUAK1 interactome with either the RNAPII or MYC interactome was limited to a small number of proteins (Figure S1F). Notably, a nuclear PP1 regulatory subunit, PNUTS, that was previously found to interact with RNAPII (Ciurciu et al., 2013), was also found in the NUAK1 interactome (see below) and interacted with SF3B1 in PLA assays (Figure 1I). We therefore explored further the interaction of NUAK1 with nuclear PP1 complexes.

PNUTS Interacts with and Is Phosphorylated by NUAK1 in the Nucleus

In the cytoplasm, NUAK1 controls the function of PP1 holoenzymes by phosphorylating the MYPT1 regulatory subunit, thereby altering its function and localization (Zagórska et al., 2010). In the nucleus, the catalytic subunit of PP1 interacts with one of three major subunits: Repo-Man (encoded by the *CDCA2* gene), PNUTS (PP1-nuclear targeting subunit, encoded by *PPP1R10*), and NIPP1 (nuclear inhibitor of PP1, encoded by *PPP1R8*) (Verheyen et al., 2015). Of those, PNUTS, but neither Repo-Man nor NIPP1, was present in NUAK1 interactome (Figures 1G and S1D). This was confirmed by co-immunoprecipitation of PNUTS and HA-NUAK1 (Figure 2A). PLAs showed that NUAK1 and PNUTS interacted in the nucleus, whereas NUAK1 and MYPT1 interacted mainly in the cytoplasm (Figures 2B and S2A). Like NUAK1 and PPP1CB, the bulk of PNUTS was bound to chromatin (Figures 1B and 1D), and like NUAK1, PNUTS interacted with SF3B1 in PLA assays (Figure 1I). NUAK1 interacts directly with the catalytic subunit of PP1 holoenzymes via a conserved four amino acid motif (GILK) (Zagórska et al., 2010) (Figure 2C). Consistently, incubation of cells with a corresponding peptide abolished the interaction of NUAK1 with both PPP1CB and PNUTS, as documented by PLAs (Figure 2D). Intriguingly, a phosphoproteomic analysis of NUAK1-depleted cells (see below) showed that serine 313 of PNUTS is a potential target site of NUAK1. To confirm this hypothesis, we raised a phospho-specific antibody against this site. Immunoblots of lysates of transfected cells confirmed that the antibody recognizes wild-type PNUTS, but neither S313A, S313D, nor S313E mutant PNUTS expressed at equal levels (Figure 2E). Depletion of NUAK1 using three different short hairpin RNAs (shRNAs) (Figure 2F) or a small interfering RNA (siRNA) (Figure S2B) decreased phosphorylation of endogenous PNUTS at S313 but had no effect on total PNUTS levels. We concluded that NUAK1 interacts with nuclear PNUTS/PPP1CB complexes and phosphorylates PNUTS on S313.

Regulatory subunits such as PNUTS can either target PP1 catalytic subunits to specific sites or inhibit PP1 activity at specific subcellular localizations (Verbinnen et al., 2017). To determine how NUAK1 affects PP1 activity, we performed phosphoproteomic analyses of NUAK1-depleted U2OS cells. The analysis showed that siRNA-mediated depletion of NUAK1 altered the phosphorylation of a large set of nuclear proteins (Figure 2G). Specifically, depletion of NUAK1 downregulated phosphorylation of many proteins that interact with PP1, suggesting that NUAK1 inhibits their dephosphorylation (Figure 2H). A GO term analysis showed that differentially phosphorylated proteins are broadly involved in RNA processing (Figure 2I). In line with the function of proteins identified in the NUAK1 interactome, a subset of differentially phosphorylated proteins is involved in RNA processing and splicing; this includes, for example, SRRM2, a protein identified as a strong NUAK1 interactor (Figure S1D). Finally, depletion of NUAK1 also altered the phosphorylation of multiple proteins not found in the PP1 interactome, arguing that NUAK1 also has PP1-independent effects and that some changes in the phosphoproteome are indirect. We concluded that NUAK1 associates with nuclear PP1 holoenzymes and the spliceosome and is required for phosphorylation of multiple proteins involved in RNA processing.

PNUTS Binds Chromatin via RNA and Promotes Spliceosome Activity

To better understand how PNUTS, PPP1CB, and NUAK1 interact with chromatin, we performed fractionation experiments upon treatment of nuclear extracts with RNase A, which discriminates resident chromatin proteins from proteins that interact with chromatin indirectly via RNA. As expected, treatment with RNase A released a significant fraction of the splicing factor SF3B1 and the spliceosome-associated NIPP1 protein from chromatin, while actively transcribing (phosphorylated) RNAPII or histone H2B remained bound to chromatin (Figures 3A and S3A). Intriguingly, RNase A released a significant fraction of PNUTS and PPP1CB from chromatin, arguing that both proteins are bound to chromatin at least in part via association with RNA (Figures 3A and S3A). In contrast, RNase A treatment did not affect chromatin association of NUAK1 (Figures 3A and S3A).

The dependence of chromatin association of PNUTS on RNA is consistent with the presence of an RNA-binding domain in PNUTS and with previous observations that long noncoding RNA (lncRNA) molecules can target PNUTS to specific genes and that PNUTS binds nascent RNA (Bao et al., 2018; Kim

(E) Immunoblot using the indicated antibodies of U2OS cells transfected with pcDNA3 vectors encoding HA-tagged rat wild-type or S313A/D/E-mutated PNUTS; EV was used as negative control. In the α-pS313-PNUTS panel, the upper band represents endogenous PNUTS, while the lower is the exogenous rat protein. VCL was used as loading control (n = 3).
(F) U2OS cells were infected with three independent doxycycline (DOX)-inducible shRNAs targeting *NUAK1* and, where indicated, treated with DOX (1 μg/mL) for 24 h. Asterisk denotes unspecific band (n = 3). Bottom: immunoblot of NUAK1 confirming its depletion. VCL was used as loading control (n = 3).
(G) Volcano plot showing differentially regulated phosphosites and the functional annotation of respective proteins in a spike-in SILAC phosphoproteomic analysis upon transfection of a siRNA pool targeting *NUAK1* mRNA (siNUAK1). Significance is indicated by the dashed line ($p < 0.05$) (n = 3).
(H) Waterfall plot showing differentially spike-in SILAC-labeled phosphorylated residues ($p < 0.05$) upon NUAK1 depletion. Orange, phosphosites of PP1-interacting proteins (n = 3).
(I) Differentially phosphorylated residues upon NUAK1 depletion (n = 197, $p < 0.05$) were used as input for a GO term analysis (left: cell component; right: biological function). FDR, false discovery rate; fold enr., fold enrichment.
See also Figure S2.

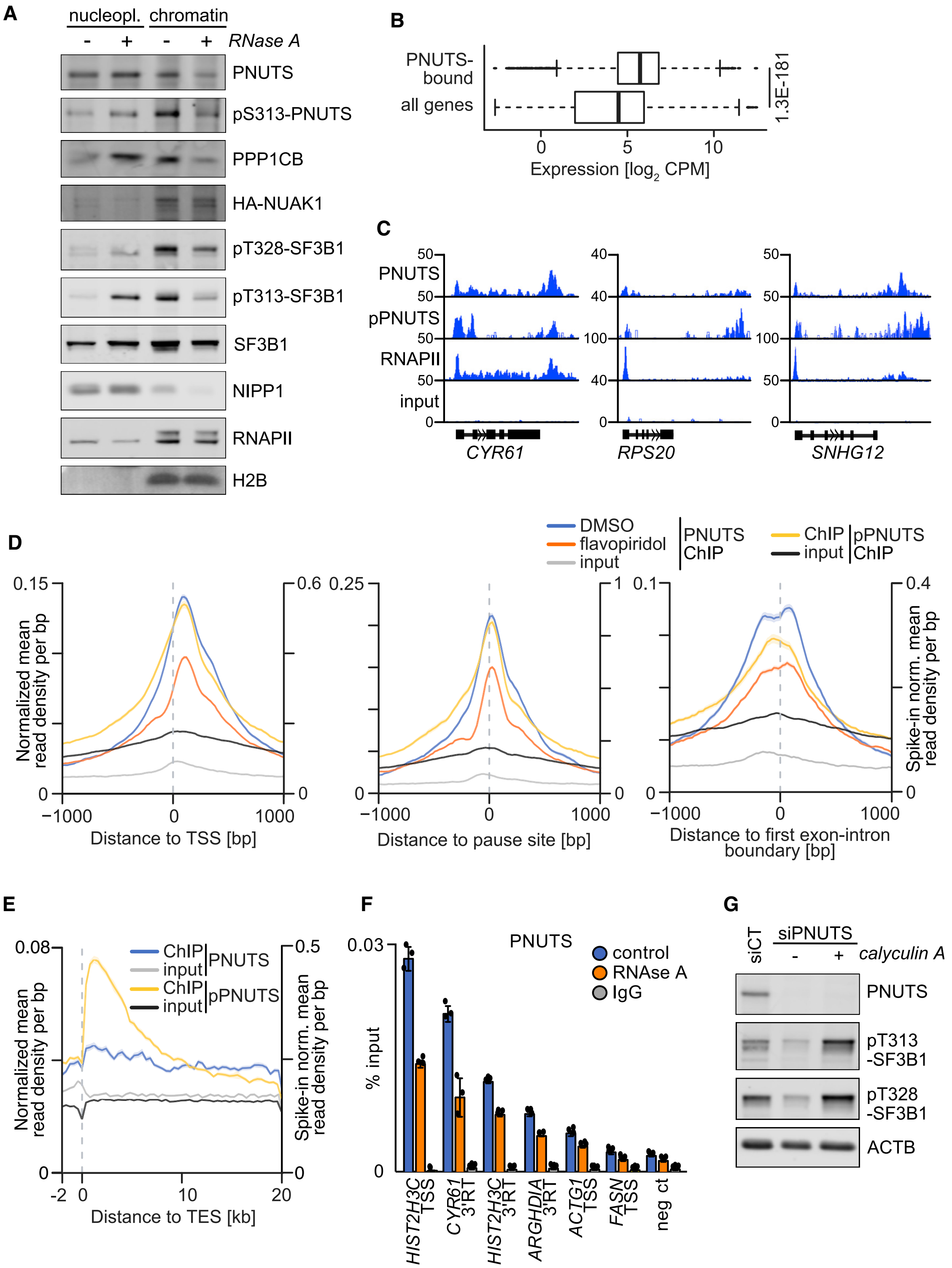

Figure 3. PNUTS Binds Chromatin via RNA and Promotes Spliceosome Activity

(A) Immunoblot documenting chromatin association of the indicated proteins in control cell lysates and in lysates upon RNase A treatment. Cell fractionation was performed on U2OS cells expressing HA-tagged NUAK1. Nucleopl., nucleoplasmic fraction; chromatin, chromatin-bound fraction. SF3B1 and NIPP1 or phosphorylated RNAPII and H2B were used as RNA- and chromatin-bound controls, respectively (n = 3).

(B) Expression of PNUTS-bound genes (n = 2,786) versus all expressed genes (n = 19,382). The p value was calculated with a two-tailed Wilcoxon rank-sum test. CPM, counts per million.

(legend continued on next page)

et al., 2003; Xing et al., 2014). To determine the sites on chromatin to which PNUTS is bound in an unbiased manner, we performed chromatin immunoprecipitation followed by sequencing (ChIP-seq). A correlation of ChIP-seq data with RNA sequencing (RNA-seq) data showed that PNUTS-bound genes display relatively high levels of expression but did not reveal a significant enrichment of specific functional categories of PNUTS-bound genes (Figure 3B). Importantly, ChIP-seq showed that PNUTS bound both downstream of the transcription start site (TSS) of 2,571 genes and close to the transcription end site (TES) at 584 genes, as demonstrated both by inspection of multiple individual genes and global analyses (Figures 3C–3E). PNUTS binding downstream of the TSS peaked around the RNAPII pause site and both immediately 5′ and 3′ of the first exon-intron boundary (Figure 3D). Previous observations have implicated PNUTS in transcription termination (Austenaa et al., 2015; Cortazar et al., 2019). Consistently, a major PNUTS peak was observed around the TES (Figures 3C and 3E). Our analysis also confirmed previous observations that PNUTS avidly binds histone clusters (Figure S3B) (Verheyen et al., 2015). ChIP-seq with exogenous reference genome spike-in (ChIP-RX) using the phospho-specific antibody showed that chromatin association of phosphorylated pS313-PNUTS closely resembled that of total PNUTS (Figures 3C–3E). ChIP experiments upon RNase A confirmed that PNUTS bound to both promoter-proximal and TES in part via interaction with RNA (Figure 3F). Consistent with the observation that PNUTS is part of the interactome of newly transcribed RNA (Bao et al., 2018), treatment of cells with flavopiridol (FP), an inhibitor of the CDK9 kinase that globally blocks nascent RNA synthesis, attenuated PNUTS chromatin binding (Figures 3D and S3C). We concluded that PNUTS binds chromatin in part via association with nascent RNA.

The localization of PNUTS binding, its dependency on RNA, and the co-precipitation of PNUTS with spliceosomal proteins suggested that PNUTS has a role in splicing. Indeed, PP1 activity affects the phosphorylation status of several splicing factors, thereby regulating both spliceosome assembly and its catalytic cycle (Aubol et al., 2017; Shi et al., 2006). To test this hypothesis, we used two phospho-specific antibodies that recognize phosphorylated T313 and T328 in the TP-rich domain of SF3B1 (Figure S3E); these residues are phosphorylated exclusively in catalytically active spliceosomes (Girard et al., 2012). Indeed, the U2 spliceosome component SF3B1 is a well-described PP1 target, which is hyperphosphorylated during the first step of catalysis and dephosphorylated during the second one (Girard et al., 2012; Shi et al., 2006; Tanuma et al., 2008). Depletion of PNUTS strongly reduced phosphorylation at both sites and inhibition of PP1 using the phosphatase inhibitors calyculin A and okadaic acid (which target both PP1 and PP2A phosphatases) reverted the inhibition (Figures 3G and S3D). Collectively, the data indicate that PNUTS locally inhibits PP1 downstream of the TSS of actively transcribed genes to promote spliceosome activity.

NUAK1 Controls Chromatin Association of PNUTS

To understand whether NUAK1 affects PNUTS function, we incubated U2OS cells with the GILK peptide that blocks NUAK1 interaction with PP1 and found that this led to a strong decrease in SF3B1 phosphorylation (Figure 4A), indicating that NUAK1 or a structurally related kinase is required for spliceosome activity. Consistently, both shRNA- and siRNA-mediated depletion of NUAK1 reduced SF3B1 phosphorylation at T313 and T328, although the effects were not as strong as with the GILK peptide (Figures 4B and 4C). We reasoned that this might be due to the relatively slow kinetics of a depletion experiment, which might allow cells to adapt to a decrease in NUAK1 activity. To test this hypothesis, we used two chemically distinct small molecules to acutely inhibit NUAK1. The first is BAY-880, which we identified as a potent inhibitor of NUAK1 that inhibits 96% of its kinase activity at 1 μM concentration (Figures S4A and S4B; Table S1). Testing BAY-880 against a panel of 274 kinases showed that NUAK1 is the best target and identified a small number of additional kinases that may be inhibited by this compound (Figure S4B; Table S1). Consistent with this, a phosphoproteomic analysis showed that the changes in phosphorylation induced by siRNA-mediated depletion of NUAK1 in U2OS cells showed a statistically highly significant overlap with changes induced by BAY-880 (Figure 4D; $p = 1.5 \times 10^{-13}$), and a GO term analysis of changes of differentially regulated phosphosites showed that processes targeted by BAY-880 highly overlapped with the ones targeted by depletion of NUAK1 (Figure 4D). Importantly, both depletion of NUAK1 and BAY-880 jointly targeted multiple proteins involved in RNA metabolism and, specifically, RNA splicing, 3′ end processing, and localization (Figures 4D and S4C). In addition, we used a well-characterized NUAK1 inhibitor, HTH-01-015 (Banerjee et al., 2014a). Notably, potential off-target activities of BAY-880 greatly differed from those of HTH-01-015, arguing that joint targets of both inhibitors reflect on-target effects resulting from NUAK1 inhibition (Figure S4B). We therefore confirmed that both compounds decrease phosphorylation of S313-PNUTS and observed that BAY-880 was more potent than HTH-01-015 in inhibiting NUAK1 activity (Figures 4E and S4D).

(C) Genome Browser tracks showing PNUTS, phospho-S313-PNUTS (pPNUTS), and RNAPII binding to representative genes. Input tracks are included as control.

(D) Average density plots of PNUTS ChIP-seq (left y axis) and pPNUTS ChIP-RX (right y axis). The shadow around tracks indicates SEM. TSS, transcription start site.

(E) Average density plots of PNUTS ChIP-seq (left y axis) and pPNUTS ChIP-RX (right y axis) centered to transcription end site (TES). The shadow around tracks indicates SEM.

(F) PNUTS ChIP performed upon RNase A treatment. IgG ChIP was used as antibody specificity control. TSS, transcription start site; 3′RT, 3′ readthrough site; neg ct, negative control (mean ± SD of technical triplicates of a representative experiment; n = 3).

(G) Immunoblots documenting phosphorylation of SF3B1 at the indicated sites. U2OS cells were transfected with a siRNA pool targeting PNUTS and, 48 h later, treated with 25 nM calyculin A for 30 min. ACTB was used as loading control (n = 3).

See also Figure S3.

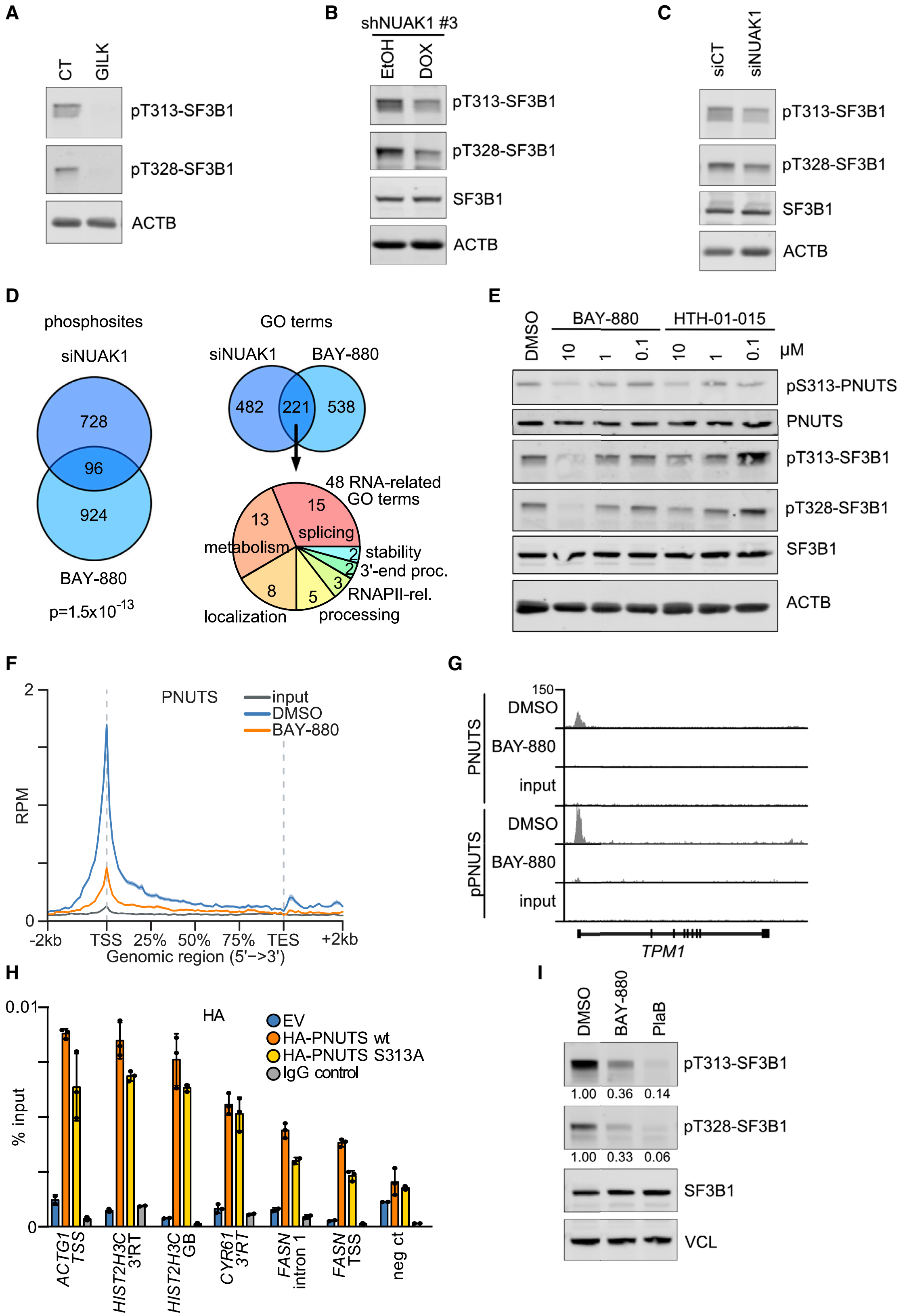

(legend on next page)

Phosphoproteomic analyses showed that acute inhibition by either inhibitor targeted highly overlapping sets of RNA-processing proteins, as observed in response to depletion of NUAK1 (Figures S4E–S4G; compare with Figure S4C). A potentially relevant off-target activity of BAY-880 is inhibition of CDK9; also, PNUTS has been implicated in the phosphorylation of the CTD of RNAPII at serine 5 (Ciurciu et al., 2013). However, both BAY-880 and HTH-01-015 had only minor effects on S2- or S5-RNAPII phosphorylation, while the CDK9 inhibitors FP and LDC00067 or inhibitors of other transcription-associated CDKs essentially abolished phosphorylation at S2 (Figure S5A). Furthermore, comparison of the phosphosites identified by phosphoproteomics upon BAY-880 or siRNA-mediated depletion of NUAK1 with recently described CDK9 targets (Sansó et al., 2016) showed only a statistically nonsignificant overlap (Figure S5B). We concluded that the effects of both drugs are not mediated via inhibition of CDK9 and that NUAK1 has no direct role in RNAPII phosphorylation. Both NUAK1 inhibitors reduced phosphorylation of SF3B1 at the sites that indicate spliceosome activity; the decrease in phosphorylation was stronger in response to BAY-880, correlating with the stronger inhibition of NUAK1-dependent phosphorylation of S313-PNUTS (Figure 4E).

PNUTS ChIP-seq and pS313-PNUTS ChIP-RX showed that chromatin association both at TSS and TES was strongly reduced by inhibition of NUAK1 (Figures 4F, 4G, and S5C). Comparison of chromatin binding of wild-type PNUTS with S313A-mutated PNUTS showed that this decrease was due partly to the decreased phosphorylation at this site, but the magnitude in the decrease observed after NUAK1 inhibition revealed that other events (e.g., changes in spliceosomal proteins) contribute to the decrease in association (Figure 4H). In contrast, S313A-PNUTS showed no difference to wild-type PNUTS in terms of binding to PP1 and nuclear localization (not shown). Finally, BAY-880 effect on splicing activity was compared with that of pladienolide B (PlaB), a well-characterized splicing inhibitor (Kaida et al., 2007; Kotake et al., 2007). PlaB abolished SF3B1 phosphorylation, while BAY-880 inhibited SF3B1 phosphorylation by 50%–70%. (Figures 4I and S5D). Notably, PlaB did not affect S313-PNUTS phosphorylation, confirming that PlaB activity on SF3B1 is PNUTS-independent (Figure S5D) (Kotake et al., 2007). Interestingly, dephosphorylation of S313 upon NUAK1 inhibition by either BAY-880 or HTH 01-015 was essentially complete within 1 h treatment (Figure S5D), suggesting that the effects of the inhibitors on PNUTS or SF3B1 phosphorylation are direct. We concluded that phosphorylation by NUAK1 promotes chromatin association of PNUTS and spliceosome activity.

NUAK1 Promotes Splicing and Transcription Termination

To determine whether NUAK1 is required for co-transcriptional splicing, we labeled nascent RNA using a pulse of 15 min with 4-thiouridine (4sU) and sequenced labeled RNA either immediately (pulse) or 2 h after 4sU withdrawal (chase; Figure S5E). The experiment was performed in control (DMSO) cells as well as in the presence of BAY-880 or PlaB. As expected, the percentage of exonic and spliced reads strongly increased during the chase, reflecting processing of pre-mRNA (Figure 5A). PlaB blocked the increase in exonic and spliced reads and caused a marked increase in intronic reads, indicating that PlaB blocked splicing (Figure 5A). Similarly, inhibition of NUAK1 impaired the increase in spliced and exonic reads, although the effect was weaker than for PlaB (Figure 5A). Inspection of individual genes confirmed this observation (Figure 5B). Plotting the number of spliced reads per gene confirmed that the number of reads per gene increased during the chase in control (DMSO) cells but remained constant in BAY-880-treated cells and slightly decreased in PlaB-treated cells (Figure 5C).

The weaker effect of BAY-880 on overall splicing relative to that of PlaB is consistent with the lesser extent of BAY-880-mediated SF3B1 dephosphorylation (Figures 4I and S5D). We reasoned that the role of NUAK1 might be restricted to a subset of genes, compared with the genome-wide effect of PlaB. We therefore performed a ranked sum gene set enrichment analysis (GSEA) (Subramanian et al., 2005), ranking genes on the basis of

Figure 4. NUAK1 Controls Chromatin Association of PNUTS

(A) Immunoblots documenting phosphorylation of SF3B1 at the indicated sites. U2OS cells were treated 4 h with 50 μM GILK (or control [CT]) peptide. ACTB was used as loading control (n = 3).

(B) Immunoblots documenting phosphorylation of SF3B1 at the indicated sites. U2OS cells stably expressing a doxycycline (DOX)-inducible shRNA targeting *NUAK1* mRNA (shNUAK1 #3 in Figure 2F) was induced with DOX for 24 h. ACTB was used as loading control (n = 3).

(C) Same as B, but using and siRNA pool targeting NUAK1 (siNUAK1) or control siRNA pool. ACTB was used as loading control (n = 3).

(D) Left: Venn diagram showing the overlap between significantly differentially regulated phosphosites identified in response to siRNA-mediated NUAK1 depletion (48 h) or treatment with 10 μM BAY-880 (2 h) in a TMT phosphoproteomic experiment. Right: GO term analysis of differentially phosphorylated proteins. At the top is a Venn diagram showing the overlap between all identified GO terms; below is a pie chart of categories of 48 RNA-related GO terms.

(E) Immunoblots documenting phosphorylation of PNUTS at S313 and of SF3B1 at the indicated sites after 24 h incubation of U2OS cells with the indicated concentrations of BAY-880 or HTH-01-015. ACTB was used as loading control (n = 3).

(F) Read density plot analysis of PNUTS ChIP-seq upon 4 h 10 μM BAY-880 treatment (n = 3,172 PNUTS-bound genes). The shadow around tracks indicates SEM. TSS, transcription start site; TES, transcription end site.

(G) Genome Browser track at the *TPM1* gene of PNUTS ChIP-seq and phospho-S313-PNUTS (pPNUTS) ChIP-RX from U2OS cells treated 4 h with 10 μM BAY-880. Input tracks are included as control.

(H) ChIP experiments using an α-HA antibody showing chromatin association of wild-type PNUTS and of PNUTS S313A after transfection in U2OS cells of expression plasmids encoding HA-tagged PNUTS or empty vector (EV). IgG ChIP was used as antibody specificity control. TSS, transcription start site; 3′RT, 3′ readthrough site; neg ct, negative control (mean ± SD of technical triplicates of a representative experiment; n = 3).

(I) Immunoblots documenting phosphorylation of SF3B1 at the indicated sites after treatment of U2OS cells with 10 μM BAY-880 or 1 μM pladienolide B (PlaB) for 4 h. VCL was used as loading control. Quantification of T313- and T328-SF3B1 bands was compared with DMSO-treated samples and normalized to VCL band intensity from three independent experiments.

See also Figures S4 and S5 and Table S1.

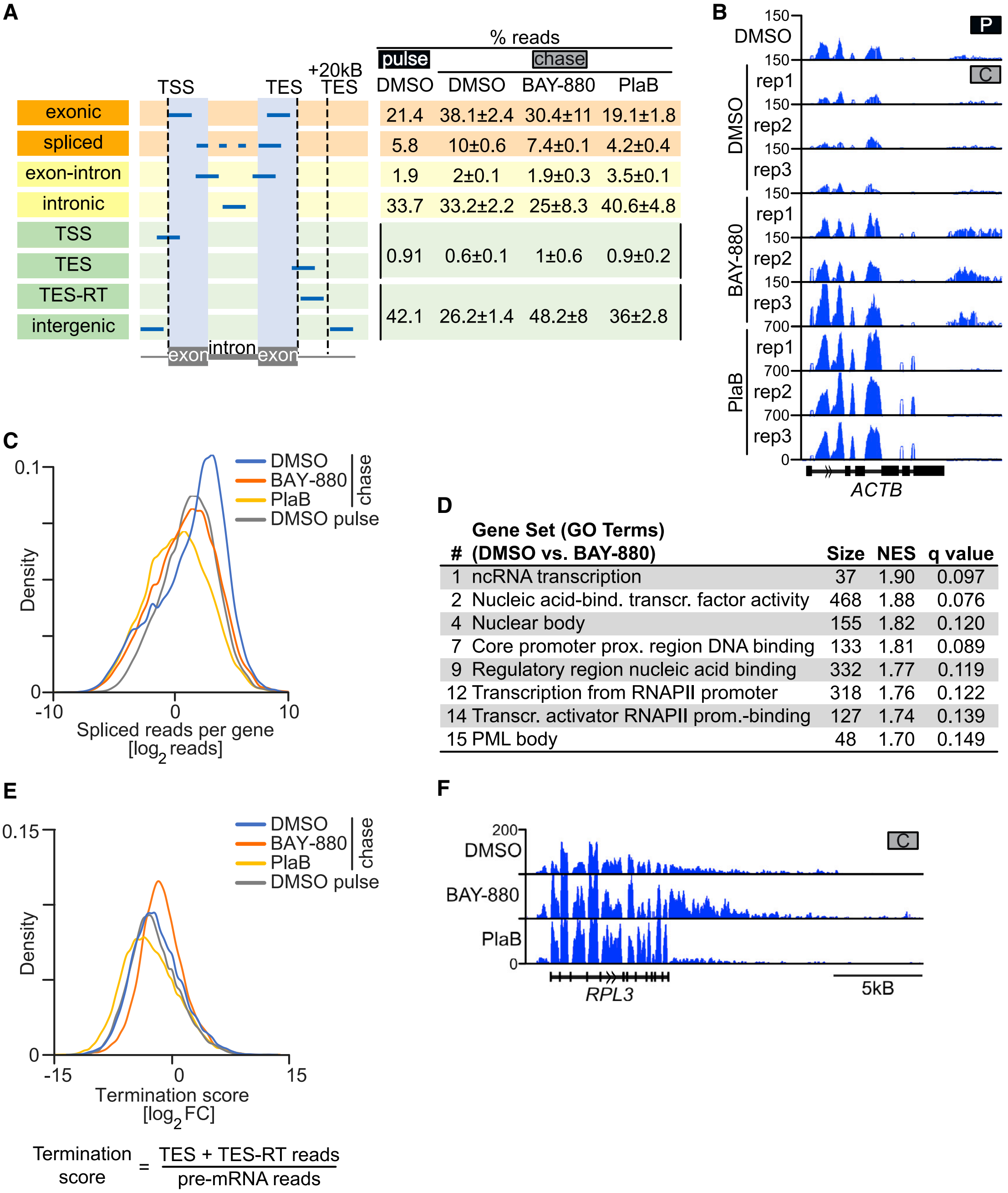

Figure 5. NUAK1 Promotes Splicing and Transcription Termination

(A) Left: definition of read categories; orange reads represent mature mRNA, yellow reads pre-mRNA. Right: percentage (average ± SD) of reads identified in the nascent RNA-seq analysis described in Figure S5F.

(B) Genome Browser tracks of 4sU-labeled RNA recovered from a pulse-chase experiment performed as described in Figure S5F. For each chased (C) sample, three replicates (rep) are reported. Tracks were first normalized to overall reads, then exonic reads were electronically removed.

(C) Kernel density plot of the number of reads harboring splice junctions (spliced reads; see A). Read counts were normalized to the number of exons per gene and the bandwidth was set to 0.3. Genes without spliced reads were removed. The mean over all replicates was plotted (DMSO, n = 16,257; BAY-880, n = 14,602; PlaB, n = 13,216; DMSO pulse, n = 13,249).

(D) Gene sets identified by a GSEA on GO terms of genes showing splicing defects upon NUAK1 inhibition. Genes were ranked according to their splicing score. Splicing score was defined as the ratio between reads harboring splice junctions (spliced reads; see A) and pre-mRNA reads (reads falling into introns and intron-exon-spanning reads; yellow in A).

(legend continued on next page)

a "splicing score" (defined as the ratio between spliced reads and pre-mRNA reads). Intriguingly, this analysis showed that the gene sets most strongly affected by NUAK1 inhibition were enriched for genes encoding proteins of the basic transcription machinery and nuclear structure (Figure 5D). Inspection of individual genes in the top enriched gene sets illustrated the strong effect of NUAK1 inhibition on intron retention of these mRNAs (Figure S5F). The data suggest that expression of genes of the core transcriptional machinery is particularly sensitive to defects in NUAK1 activity.

Incubation of cells with BAY-880, but not with PlaB, also led to a strong increase in intergenic reads (Figure 5A). As described before, PNUTS also controls transcription termination (Austenaa et al., 2015; Cortazar et al., 2019). Consistent with these observations, BAY-880 induced a marked increase of 3′ readthrough reads (TES-RT; positioned 3′ of the TES), representing inaccurately terminated transcripts (Figures 5E and 5F). We concluded that NUAK1 promotes both termination genome-wide and splicing of a specific subset of genes encoding proteins of the transcription machinery.

NUAK1 Controls a MYC-Sensitive Feedback Control of Transcriptional Elongation

To understand whether the role of NUAK1 in splicing affects transcription, we performed both RNAPII ChIP-RX and 4sU pulse labeling of nascent RNA. In control cells, NUAK1 inhibition had no significant effect on RNAPII association with the TSS, the pause site, or the first exon-intron boundary (Figures 6A and 6B). In contrast, NUAK1 inhibition decreased overall RNAPII occupancy at the TES, suggesting that transcription elongation is impaired on many genes (Figure 6C). Consistent with this interpretation, we noted that overall nascent RNA synthesis, as tested by pulse labeling with 4sU, was significantly reduced upon NUAK1 inhibition (Figures 7A and 7B).

To understand whether elevated levels of MYC influence these effects, we performed 4sU labeling and RNAPII ChIP-RX experiments on samples treated with BAY-880 16 h after the activation of a MYC-ER chimera. We first validated that induction of MYC did not alleviate the dependence of SF3B1 phosphorylation on NUAK1 (Figure S6A), indicating that it did not alter the dependence of spliceosome activity on NUAK1. Analysis of nascent RNA synthesis by a pulse of 4sU showed that activation of MYC strongly enhanced nascent RNA synthesis (Figures 7A, 7B, and S6B). In addition, activation of MYC strongly attenuated the decrease in nascent RNA synthesis caused by inhibition of NUAK1 on multiple genes (Figure 7A). Global analyses showed that MYC increased nascent RNA synthesis throughout the gene body in control cells; in contrast, the MYC-dependent increase was confined to promoter-proximal regions in the presence of BAY-880 (Figure 7B). Activation of MYC did not attenuate or override the block in transcription elongation caused by inhibition of CDK9 using either FP or NVP-2, demonstrating that MYC specifically overrides the inhibition of nascent RNA synthesis caused by BAY-880 (Figures 7B and S6C). Remarkably and consistent with the 4sU data, RNAPII globally accumulated downstream of the TSS upon NUAK1 inhibition in cells with high levels of MYC (Figures 6A and 6B). Specifically, RNAPII accumulated at the pause site and both 5′ and 3′ of the first exon-intron boundary (Figure 6B). This localization closely paralleled the chromatin association of PNUTS (compare Figure 3D with Figure 6B; Figure S6D). Comparison with published data describing the changes in RNAPII chromatin association in response to CDK9 inhibition (Olson et al., 2018) showed that although inhibition of CDK9 causes an accumulation of RNAPII at the pause site, the peaks 5′ and 3′ of the first exon-intron boundary were observed only in response to inhibition of NUAK1 (Figure S6E). We suggest that this accumulation reflects a delay or block in transcription elongation in response to a defect in mRNA splicing caused by inhibition of NUAK1.

Notably, RNA-seq showed that the accumulation of RNAPII upon NUAK1 inhibition in cells with high MYC levels was not paralleled by an increase in mRNA synthesis, arguing that the accumulating RNAPII is non-productive (Figure S7A). Consistently, RNAPII ChIP-RX showed that NUAK1 inhibition strongly suppressed RNAPII occupancy at the TES also in cells with active MYC (Figure 6C). Taken together, we concluded that inhibition of spliceosome assembly exerts a negative control on pause release or early steps of transcription elongation, which is overridden by elevated levels of MYC, leading to the accumulation of RNAPII at the first exon-intron boundary. The 4sU pulse-chase experiment also showed that inhibition of NUAK1 caused a striking increase in TES-RT/intergenic reads in both control and high-MYC cells, both on a global scale and when inspecting individual genes, consistent with the role of NUAK1 and PNUTs in termination (Figures S7B and S7C).

To understand how inhibition of NUAK1 exerts the effects on early elongation, we reasoned that splicing defects can lead to the accumulation of stable hybrids of nascent RNA with DNA, termed R-loops (Chen et al., 2018). To analyze this, we initially chose two genes that require NUAK1 for splicing, *ACTB* and *NCL* (Figures 7C and S7D). Comparison with a recently published genome-wide sequencing dataset obtained from U2OS cells showed that R-loops are widespread over both genes (De Magis et al., 2019). Immunoprecipitations using a monoclonal antibody (S9.6) that specifically recognizes DNA-RNA hybrids showed that inhibition of NUAK1 caused R-loop accumulation at both *ACTB* and *NCL* gene loci (Figure 7D) and at multiple other genes bearing R-loops, as shown in the genome-wide analysis (Figures 7D and S7E). Incubation of chromatin with RNase H, which selectively degrades DNA-RNA hybrids, removed the signal, confirming its specificity

(E) Top: kernel density plot of the termination score. The mean over all replicates was plotted and the bandwidth was set to 0.3 (DMSO, n = 18,782; BAY-880, n = 18,907; PlaB, n = 17,639; DMSO pulse, n = 16,342). Bottom: definition of termination score as reads in TES or TES + 20 kb/pre-mRNA reads, whereas pre-mRNA reads are defined as all reads falling into introns and intron-exon-spanning reads (i.e., yellow in A).

(F) Genome Browser tracks of nascent RNA expression of a representative gene displaying termination readthrough. Tracks were generated as described in B (cumulative gene browser picture from three independent replicates).

See also Figure S5.

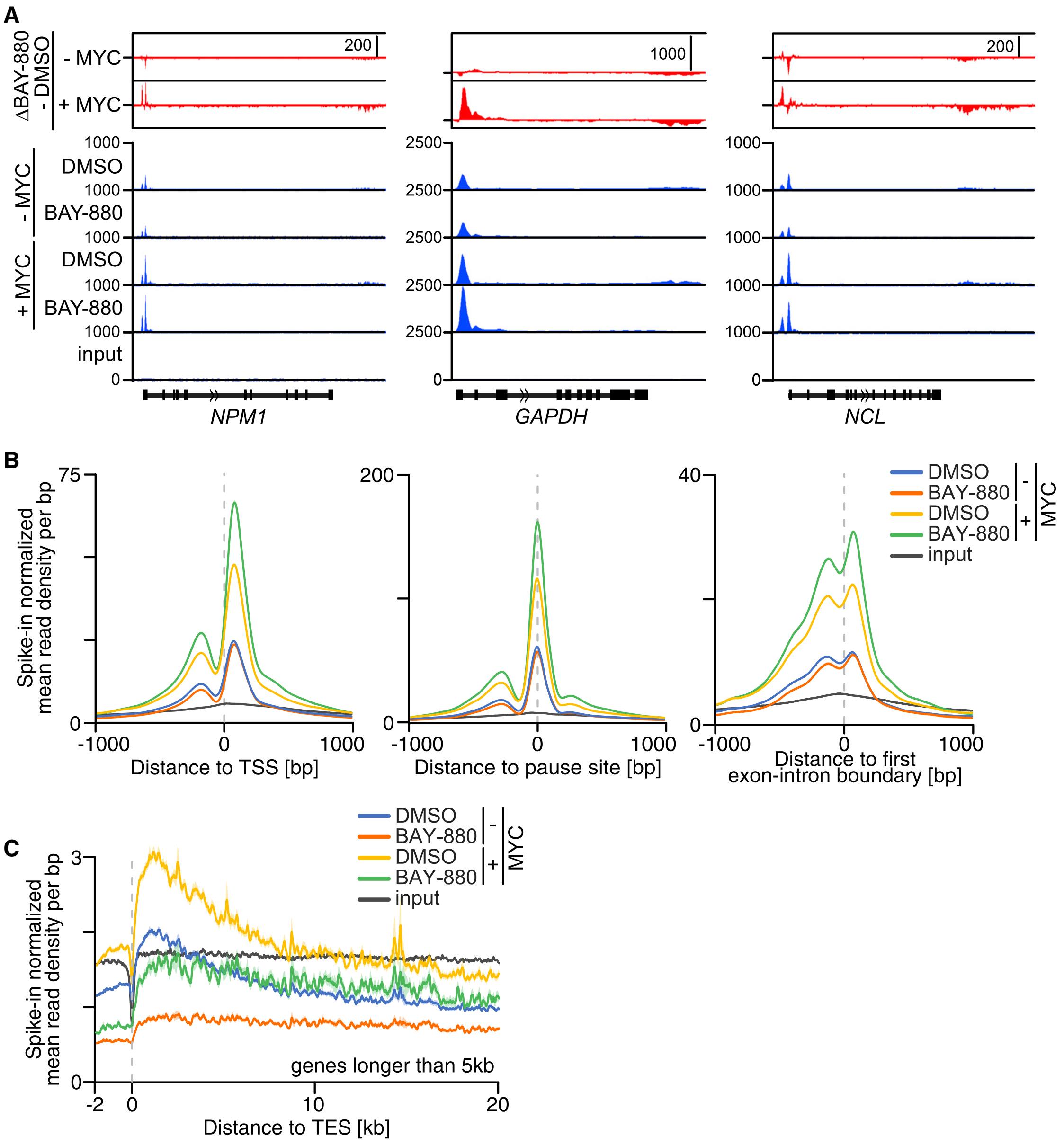

Figure 6. NUAK1 Controls RNAPII-Mediated Elongation in a MYC-Dependent Manner

(A) RNAPII occupancy at three representative genes. Blue, Genome Browser tracks of RNAPII ChIP-RX upon treatment with 4 h 10 μM BAY-880 or DMSO in control cells (− MYC) or upon 20 h MYC-ER activation with 100 nM 4-OHT (+ MYC). Red, read difference between BAY-880 and DMSO samples.

(B) Read density plots of RNAPII ChIP-RX analysis upon treatment with 4 h 10 μM BAY-880 or DMSO in control cells (− MYC) or upon 20 h MYC-ER activation with 100 nM 4-OHT (+ MYC). Plots are centered to transcription start site (TSS, left), RNAPII pause site (middle), or first exon-intron boundary (right).

(C) Read density plots of RNAPII ChIP-RX analysis upon treatment with 4 h 10 μM BAY-880 or DMSO in control cells (− MYC) or upon MYC-ER activation (+ MYC). Plots are centered to transcription end site (TES). The shadow around tracks indicates SEM.

See also Figure S6.

(Figures 7D and S7E). In neuroblastoma cells, R-loop accumulation correlates with stalling of RNAPII and with recruitment of mRNA de-capping enzymes, which trigger transcription termination (Brannan et al., 2012; Herold et al., 2019). Consistent with these observations, NUAK1 inhibition led to recruitment of DCP1A, a core subunit of mRNA de-capping complexes, to several promoter-proximal regions that we tested (Figure 7E). Elevated MYC levels attenuated both R-loop accumulation and the recruitment of mRNA de-capping factors in response to NUAK1 inhibition (Figures 7D, 7E, and S7E). We concluded that inhibition of NUAK1 and spliceosome activity induces R-loop accumulation and recruitment of mRNA de-capping factors and that MYC overrides this feedback-like control, indicating a model in which MYC drives transcriptional elongation

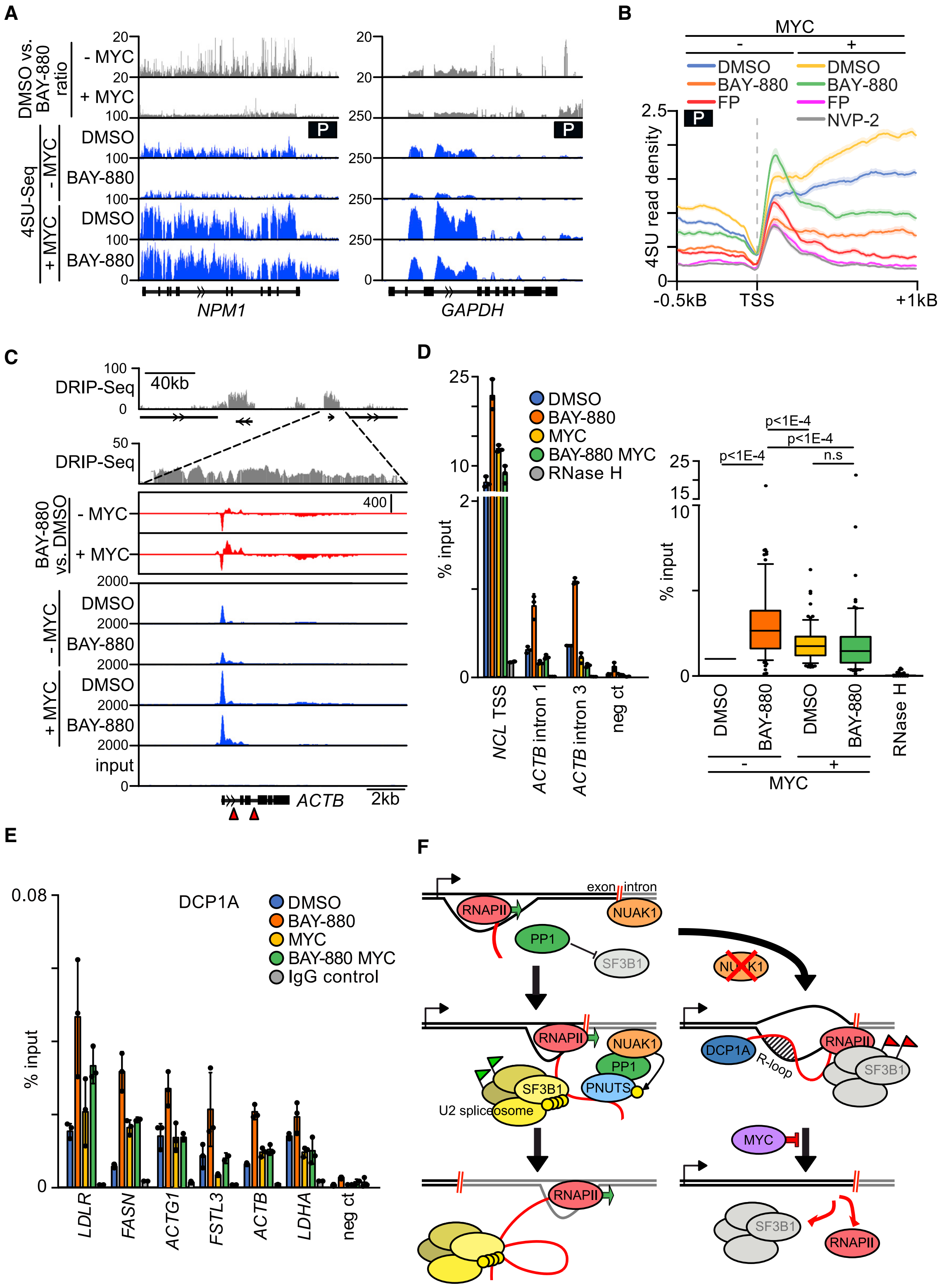

Figure 7. NUAK1 Affects Nascent RNA Synthesis, R-Loop Formation, and De-capping enzyme Recruitment in a MYC-Dependent Manner

(A) Nascent RNA synthesis of two representative genes as determined by a 15 min pulse of 4sU incorporation (P). Blue, Genome Browser tracks of nascent RNA upon treatment with 4 h 10 μM BAY-880 or DMSO in control cells (− MYC) or upon MYC-ER activation (+ MYC; 20 h). Flowchart of experiment is shown in Figure S5E. Tracks were generated as described in Figure 5B. Gray, ratio of reads in DMSO and BAY-880-treated samples.

(legend continued on next page)

in the absence of correctly assembled spliceosomes upon NUAK1 inhibition (Figure 7F).

DISCUSSION

Previous work has established that NUAK1 takes part in a trimeric complex with the catalytic subunit of PP1 and phosphorylates a regulatory subunit, MYPT1, in the cytoplasm (Zagórska et al., 2010). Here, we extend this model by showing that a large fraction of NUAK1 is localized in the cell nucleus, associates with nuclear PP1 holoenzymes, and phosphorylates one of the major nuclear targeting subunits of PP1, PNUTS, at S313. Consistent with previous work that has implicated dephosphorylation by PP1 in controlling spliceosome activity, NUAK1 associates with spliceosomes (Girard et al., 2012; Shi et al., 2006; Tanuma et al., 2008). Both PNUTS and NUAK1 are required for spliceosome activity and splicing of large sets of mRNAs. From these data, we propose a model in which PNUTS localizes to chromatin via its interaction with nascent RNA. As consequence, PNUTS comes into contact with chromatin-bound NUAK1, and its association with nascent RNA is stabilized by NUAK1-dependent phosphorylation. The stabilization locally inhibits PP1 activity toward spliceosomal proteins and enables spliceosome activation (Figure 7F). Inhibition of NUAK1 does not only impair spliceosome activity but also globally reduces nascent RNA synthesis and induces recruitment of mRNA de-capping factors. This suggests that regulation of PP1 activity also plays a critical role in coordinating transcription elongation with spliceosome activity. Previous observations have shown that elongating RNAPII associates with spliceosomes and accumulates over intron regions when spliceosome activity is perturbed (Chathoth et al., 2014; Nojima et al., 2018). Furthermore, the spliceosomal U1 RNP is part of a control mechanism that links pausing of RNAPII at the first stable nucleosome to premature polyadenylation-mediated termination (Chiu et al., 2018). Loss of PNUTS enhances phosphorylation of RNAPII at S5 in *Drosophila* embryos (Ciurciu et al., 2013). Although this may suggest a direct dephosphorylation of RNAPII by catalytically active PNUTS/PP1 holoenzymes in *Drosophila*, this increase may also be due to stalling of RNAPII (as seen in high-MYC cells) rather than to a direct role of PNUTS/PP1 in de-phosphorylating RNAPII. This interpretation is consistent with the observation that phosphorylation of S5 of RNAPII slows down elongation to promote spliceosome activity, whereas dephosphorylation of RNAPII enables rapid transcription of exonic sequences (Nojima et al., 2015). Our data argue that the localized inhibition of PP1 is a critical part of these checkpoint-like processes that link elongation to spliceosome activity.

Previous work has also established that a CDK9/PP1 switch is critical for transcription termination, and PP1 loss of function induces termination defects (e.g., readthrough transcription) (Cortazar et al., 2019; Kecman et al., 2018; Parua et al., 2018). Consistently, our and previously published (Austenaa et al., 2015; Cortazar et al., 2019) observations implicate PNUTS in transcription termination. The data represented here extend this model to show that NUAK1 also affects the function of PNUTS in transcription termination.

Previous work both in tissue culture and *in vivo* has established that MYC-driven cells and tumors depend on NUAK1 for growth (Liu et al., 2012; Monteverde et al., 2018; Port et al., 2018). We did not observe any effect of NUAK1 depletion or inhibition on MYC phosphorylation at S62 and T58 or MYC stability or an association of NUAK1 with MYC, hence it is unlikely that the dependence of cells expressing deregulated MYC on NUAK1 reflects a previously described role of PNUTS in MYC turnover (Dingar et al., 2018). We show here that activation of MYC did not remove the requirement for NUAK1 in spliceosome activity. However, MYC strongly attenuated the reduction in nascent RNA synthesis, abolished the recruitment of de-capping complexes, and suppressed accumulation of R-loops upon NUAK1 inhibition at all loci we tested. Upon NUAK1 inhibition in cells expressing high MYC levels, RNAPII did not terminate transcription but accumulated both at the pause site and at the first exon-intron boundary. Notably, this increase in RNAPII association caused by NUAK1 inhibition was not mirrored by a corresponding increase in RNA synthesis, arguing that RNAPII accumulated in a non-productive form. Our data therefore suggest a model in which the perturbed spliceosome function upon NUAK1 inhibition induces RNAPII to terminate in cells with physiological MYC levels. In contrast, upon NUAK1 inhibition in MYC-driven tumor cells termination is suppressed and RNAPII is trapped in a form that is not involved in productive transcription (Figure 7F).

(B) Transcription start site (TSS)-centered read density plot (n = 6,133) of 4sU-labeled nascent RNA (15 min pulse; P) upon 2 h treatment with 10 μM BAY-880, 1 μM flavopiridol (FP), 1 μM NVP-2, or DMSO in control cells (− MYC) or upon MYC activation (+ MYC; 18 h). The shadow around tracks indicates SEM.

(C) Top: Genome Browser track of a region of chromosome 7 showing DRIP(DNA-RNA-immunoprecipitation)-seq data in U2OS cells (GEO: GSE115957). Black bars, genes. Magnification shows detail of *ACTB* gene. Bottom: blue, Genome Browser tracks showing RNAPII occupancy at the *ACTB* gene locus upon treatment with 4 h 10 μM BAY-880 or DMSO in control cells (− MYC) or upon 20 h MYC activation with 100 nM 4-OHT (+ MYC). Red, read difference between BAY-880 and DMSO samples. Red arrows indicate the position of primers used for DRIP-qPCR (D).

(D) Left: DRIP-qPCRs of U2OS MYC-ER cells treated with DMSO or 10 μM BAY-880 for 4 h and, where indicated, co-treated with 100 nM 4-OHT for 20 h (MYC). RNase H treatment and a negative region were used to test antibody specificity. Right: box blot summarizing all performed DRIP-qPCR analyses of U2OS MYC-ER cells treated as in the left plot. The plot shows average of 38 genetic loci (Figure S7I) tested in three biologically independent experiments. All sets of data were normalized to their respective DMSO/-MYC condition. Dots represent values in the 10th to 90th percentiles. Wilcoxon matched-pairs signed rank tests were performed to compare the different conditions (n.s., not significant).

(E) DCP1A ChIP of U2OS MYC-ER cells treated with DMSO or 10 μM BAY-880 for 4 h and, where indicated, co-treated with 100 nM 4-OHT for 20 h (MYC). All tested genetic loci reside at the TSS of the indicated genes. IgG ChIP and a negative region were used as antibody specificity controls. Neg ct, negative control (mean ± SD of technical triplicates of a representative experiment; n = 2).

(F) Model summarizing our findings. For details, see text.

See also Figures S6 and S7.

A strongly enhanced sensitivity of MYC-driven cells to a perturbation of the splicing machinery has been observed before in different biological systems; specifically, upregulation of the core spliceosome machinery is an essential step in MYC-driven lymphomagenesis, and MYC-driven lymphomas depend on PRMT5, an arginine methyltransferase that methylates spliceosomal proteins (Koh et al., 2015). Furthermore, genetic or pharmacological inhibition of the spliceosome *in vivo* impairs survival and tumorigenicity of MYC-dependent breast cancers (Hsu et al., 2015). Finally, activation of MYC renders cells sensitive to inhibition of the CLK2 kinase, which has been linked to alternative splicing (Iwai et al., 2018). Similarly, the extreme sensitivity of MYC-transformed cells to depletion of glutamine (Dang, 2011) is linked to the ability of MYC to drive transcriptional elongation in the absence of a sufficient nucleotide supply, leading to R-loop accumulation in the body of highly transcribed genes (Dejure et al., 2017). The notion that deregulated expression of MYC strongly sensitizes tumor cells toward a wide range of pro-apoptotic stimuli is considered a mechanism that protects from tumorigenesis (Lowe et al., 2004). Although the induction of individual target genes by MYC, such as BIM1, contributes to this sensitization (Muthalagu et al., 2014), the aggregate of available data argues that tumor cells that express elevated MYC levels ignore checkpoints that restrict early transcription and that the ensuing trapping of RNAPII is a common mechanism underlying these well-documented vulnerabilities of cells expressing oncogenic levels of MYC, for example by causing conflicts with the replication fork during S-phase that lead to double-strand breaks (Hamperl and Cimprich, 2016).

STAR★METHODS

Detailed methods are provided in the online version of this paper and include the following:

- KEY RESOURCES TABLE
- LEAD CONTACT AND MATERIALS AVAILABILITY
- EXPERIMENTAL MODEL AND SUBJECT DETAILS
  - Employed cell lines
  - Cell culture treatments
- METHOD DETAILS
  - Immunofluorescence
  - Cell fractionation
  - Immunoblotting
  - Co-immunoprecipitation
  - Proteomic analysis of NUAK1 interactors
  - Label-free phosphoproteomics
  - Spike-in SILAC phosphoproteomics
  - Tandem Mass Tag (TMT) phosphoproteomics
  - Chromatin IP (ChIP)
  - DNA-RNA immunoprecipitation (DRIP)
  - Nascent RNA analysis by 4-thiouridine (4sU) labeling
  - Directional RNA Sequencing
  - RNA extraction and quantitative PCR (qPCR)
  - Cloning
- QUANTIFICATION AND STATISTICAL ANALYSIS
  - Bioinformatic analyses and statistical analyses
- DATA AND CODE AVAILABILITY

SUPPLEMENTAL INFORMATION

Supplemental Information can be found online at https://doi.org/10.1016/j.molcel.2020.01.008.

ACKNOWLEDGMENTS

We acknowledge Andreas Schlosser at the mass spectrometry technology platform at Rudolf-Virchow-Zentrum, Würzburg, for the FLAG-NUAK1 and the label-free proteomic experiments and Amit Kumar at the Max-Delbrück-Center for Molecular Medicine, Berlin, for the stable isotope labeling with amino acids in cell culture (SILAC) phosphoproteomics. We thank Ulrike Samfass for technical support. This work was supported by grants to M.E. from the European Research Council (AuroMYC), the Federal Ministry of Education and Research (DKTK Therapeutic Targeting of MYC), and the German Research Foundation via the Research Group FOR2314 (Therapeutic Windows) and via grant SFB1309 (to Y.-C.C. and B.K.).

AUTHOR CONTRIBUTIONS

G.C. performed most experiments. I.R. and G.C. performed PLAs. C.S.-V. prepared samples for SILAC mass spectrometry. G.M., S.K., and R.S.V. processed and analyzed SILAC mass spectrometry. Y.-C.C. performed and analyzed TMT mass spectrometry. F.P., L.W., and D.M. designed, developed, and tested the specificity of the BAY-880 compound. A.B. and C.P.A. sequenced ChIP-seq, ChIP-RX, and RNA-seq libraries. S.W. and A.B. analyzed ChIP-seq and RNA-seq data. G.C., S.K., E.W., C.G., R.L., B.K., and M.E. devised, supervised, and interpreted experiments. G.C. and M.E. wrote the paper.

DECLARATION OF INTERESTS

L.W., F.P., and D.M. are employees and shareholders of Bayer AG. The other authors declare no competing interests.

Received: May 2, 2019
Revised: December 9, 2019
Accepted: January 7, 2020
Published: January 31, 2020

REFERENCES

Aubol, B.E., Hailey, K.L., Fattet, L., Jennings, P.A., and Adams, J.A. (2017). Redirecting SR protein nuclear trafficking through an allosteric platform. J. Mol. Biol. *429*, 2178–2191.

Austenaa, L.M., Barozzi, I., Simonatto, M., Masella, S., Della Chiara, G., Ghisletti, S., Curina, A., de Wit, E., Bouwman, B.A., de Pretis, S., et al. (2015). Transcription of mammalian cis-regulatory elements is restrained by actively enforced early termination. Mol. Cell *60*, 460–474.

Baluapuri, A., Hofstetter, J., Dudvarski Stankovic, N., Endres, T., Bhandare, P., Vos, S.M., Adhikari, B., Schwarz, J.D., Narain, A., Vogt, M., et al. (2019). MYC recruits SPT5 to RNA polymerase II to promote processive transcription elongation. Mol. Cell *74*, 674–687.e11.

Banerjee, S., Buhrlage, S.J., Huang, H.T., Deng, X., Zhou, W., Wang, J., Traynor, R., Prescott, A.R., Alessi, D.R., and Gray, N.S. (2014a). Characterization of WZ4003 and HTH-01-015 as selective inhibitors of the LKB1-tumour-suppressor-activated NUAK kinases. Biochem. J. *457*, 215–225.

Banerjee, S., Zagórska, A., Deak, M., Campbell, D.G., Prescott, A.R., and Alessi, D.R. (2014b). Interplay between Polo kinase, LKB1-activated NUAK1 kinase, PP1βMYPT1 phosphatase complex and the SCFβTrCP E3 ubiquitin ligase. Biochem. J. *461*, 233–245.

Bao, X., Guo, X., Yin, M., Tariq, M., Lai, Y., Kanwal, S., Zhou, J., Li, N., Lv, Y., Pulido-Quetglas, C., et al. (2018). Capturing the interactome of newly transcribed RNA. Nat. Methods *15*, 213–220.

Brannan, K., Kim, H., Erickson, B., Glover-Cutter, K., Kim, S., Fong, N., Kiemele, L., Hansen, K., Davis, R., Lykke-Andersen, J., and Bentley, D.L. (2012). mRNA decapping factors and the exonuclease Xrn2 function in widespread premature termination of RNA polymerase II transcription. Mol. Cell *46*, 311–324.

Büchel, G., Carstensen, A., Mak, K.Y., Roeschert, I., Leen, E., Sumara, O., Hofstetter, J., Herold, S., Kalb, J., Baluapuri, A., et al. (2017). Association with Aurora-A controls N-MYC-dependent promoter escape and pause release of RNA polymerase II during the cell cycle. Cell Rep. *21*, 3483–3497.

Carbon, S., Ireland, A., Mungall, C.J., Shu, S., Marshall, B., and Lewis, S.; AmiGO Hub; Web Presence Working Group (2009). AmiGO: online access to ontology and annotation data. Bioinformatics *25*, 288–289.

Chanarat, S., Seizl, M., and Strässer, K. (2011). The Prp19 complex is a novel transcription elongation factor required for TREX occupancy at transcribed genes. Genes Dev. *25*, 1147–1158.

Chathoth, K.T., Barrass, J.D., Webb, S., and Beggs, J.D. (2014). A splicing-dependent transcriptional checkpoint associated with prespliceosome formation. Mol. Cell *53*, 779–790.

Chen, L., Chen, J.Y., Huang, Y.J., Gu, Y., Qiu, J., Qian, H., Shao, C., Zhang, X., Hu, J., Li, H., et al. (2018). The augmented R-loop is a unifying mechanism for myelodysplastic syndromes induced by high-risk splicing factor mutations. Mol. Cell *69*, 412–425.e6.

Chipumuro, E., Marco, E., Christensen, C.L., Kwiatkowski, N., Zhang, T., Hatheway, C.M., Abraham, B.J., Sharma, B., Yeung, C., Altabef, A., et al. (2014). CDK7 inhibition suppresses super-enhancer-linked oncogenic transcription in MYCN-driven cancer. Cell *159*, 1126–1139.

Chiu, A.C., Suzuki, H.I., Wu, X., Mahat, D.B., Kriz, A.J., and Sharp, P.A. (2018). Transcriptional pause sites delineate stable nucleosome-associated premature polyadenylation suppressed by U1 snRNP. Mol. Cell *69*, 648–663.e7.

Christensen, C.L., Kwiatkowski, N., Abraham, B.J., Carretero, J., Al-Shahrour, F., Zhang, T., Chipumuro, E., Herter-Sprie, G.S., Akbay, E.A., Altabef, A., et al. (2014). Targeting transcriptional addictions in small cell lung cancer with a covalent CDK7 inhibitor. Cancer Cell *26*, 909–922.

Chuang, T.W., Peng, P.J., and Tarn, W.Y. (2011). The exon junction complex component Y14 modulates the activity of the methylosome in biogenesis of spliceosomal small nuclear ribonucleoproteins. J. Biol. Chem. *286*, 8722–8728.

Ciurciu, A., Duncalf, L., Jonchere, V., Lansdale, N., Vasieva, O., Glenday, P., Rudenko, A., Vissi, E., Cobbe, N., Alphey, L., and Bennett, D. (2013). PNUTS/PP1 regulates RNAPII-mediated gene expression and is necessary for developmental growth. PLoS Genet. *9*, e1003885.

Cortazar, M.A., Sheridan, R.M., Erickson, B., Fong, N., Glover-Cutter, K., Brannan, K., and Bentley, D.L. (2019). Control of RNA Pol II speed by PNUTS-PP1 and Spt5 dephosphorylation facilitates termination by a "sitting duck torpedo" mechanism. Mol. Cell *76*, 896–908.e4.

Cox, J., and Mann, M. (2008). MaxQuant enables high peptide identification rates, individualized p.p.b.-range mass accuracies and proteome-wide protein quantification. Nat. Biotechnol. *26*, 1367–1372.

Cox, J., Neuhauser, N., Michalski, A., Scheltema, R.A., Olsen, J.V., and Mann, M. (2011). Andromeda: a peptide search engine integrated into the MaxQuant environment. J. Proteome Res. *10*, 1794–1805.

Cox, J., Hein, M.Y., Luber, C.A., Paron, I., Nagaraj, N., and Mann, M. (2014). Accurate proteome-wide label-free quantification by delayed normalization and maximal peptide ratio extraction, termed MaxLFQ. Mol. Cell. Proteomics *13*, 2513–2526.

Dang, C.V. (2011). Therapeutic targeting of Myc-reprogrammed cancer cell metabolism. Cold Spring Harb. Symp. Quant. Biol. *76*, 369–374.

Dang, C.V. (2012). MYC on the path to cancer. Cell *149*, 22–35.

Dang, C.V. (2016). A time for MYC: metabolism and therapy. Cold Spring Harb. Symp. Quant. Biol. *81*, 79–83.

David, C.J., Boyne, A.R., Millhouse, S.R., and Manley, J.L. (2011). The RNA polymerase II C-terminal domain promotes splicing activation through recruitment of a U2AF65-Prp19 complex. Genes Dev. *25*, 972–983.

De Magis, A., Manzo, S.G., Russo, M., Marinello, J., Morigi, R., Sordet, O., and Capranico, G. (2019). DNA damage and genome instability by G-quadruplex ligands are mediated by R loops in human cancer cells. Proc. Natl. Acad. Sci. U S A *116*, 816–825.

de Pretis, S., Kress, T.R., Morelli, M.J., Sabò, A., Locarno, C., Verrecchia, A., Doni, M., Campaner, S., Amati, B., and Pelizzola, M. (2017). Integrative analysis of RNA polymerase II and transcriptional dynamics upon MYC activation. Genome Res. *27*, 1658–1664.

Dejure, F.R., Royla, N., Herold, S., Kalb, J., Walz, S., Ade, C.P., Mastrobuoni, G., Vanselow, J.T., Schlosser, A., Wolf, E., et al. (2017). The *MYC* mRNA 3′-UTR couples RNA polymerase II function to glutamine and ribonucleotide levels. EMBO J. *36*, 1854–1868.

Dingar, D., Tu, W.B., Resetca, D., Lourenco, C., Tamachi, A., De Melo, J., Houlahan, K.E., Kalkat, M., Chan, P.K., Boutros, P.C., et al. (2018). MYC dephosphorylation by the PP1/PNUTS phosphatase complex regulates chromatin binding and protein stability. Nat. Commun. *9*, 3502.

Fellmann, C., Zuber, J., McJunkin, K., Chang, K., Malone, C.D., Dickins, R.A., Xu, Q., Hengartner, M.O., Elledge, S.J., Hannon, G.J., and Lowe, S.W. (2011). Functional identification of optimized RNAi triggers using a massively parallel sensor assay. Mol. Cell *41*, 733–746.

Freese, N.H., Norris, D.C., and Loraine, A.E. (2016). Integrated genome browser: visual analytics platform for genomics. Bioinformatics *32*, 2089–2095.

Gao, P., Tchernyshyov, I., Chang, T.C., Lee, Y.S., Kita, K., Ochi, T., Zeller, K.I., De Marzo, A.M., Van Eyk, J.E., Mendell, J.T., and Dang, C.V. (2009). c-Myc suppression of miR-23a/b enhances mitochondrial glutaminase expression and glutamine metabolism. Nature *458*, 762–765.

Ginno, P.A., Lott, P.L., Christensen, H.C., Korf, I., and Chédin, F. (2012). R-loop formation is a distinctive characteristic of unmethylated human CpG island promoters. Mol. Cell *45*, 814–825.

Girard, C., Will, C.L., Peng, J., Makarov, E.M., Kastner, B., Lemm, I., Urlaub, H., Hartmuth, K., and Lührmann, R. (2012). Post-transcriptional spliceosomes are retained in nuclear speckles until splicing completion. Nat. Commun. *3*, 994.

Haikala, H.M., Anttila, J.M., Marques, E., Raatikainen, T., Ilander, M., Hakanen, H., Ala-Hongisto, H., Savelius, M., Balboa, D., Von Eyss, B., et al. (2019). Pharmacological reactivation of MYC-dependent apoptosis induces susceptibility to anti-PD-1 immunotherapy. Nat. Commun. *10*, 620.

Hamperl, S., and Cimprich, K.A. (2016). Conflict resolution in the genome: how transcription and replication make it work. Cell *167*, 1455–1467.

Herold, S., Kalb, J., Büchel, G., Ade, C.P., Baluapuri, A., Xu, J., Koster, J., Solvie, D., Carstensen, A., Klotz, C., et al. (2019). Recruitment of BRCA1 limits MYCN-driven accumulation of stalled RNA polymerase. Nature *567*, 545–549.

Hou, X., Liu, J.E., Liu, W., Liu, C.Y., Liu, Z.Y., and Sun, Z.Y. (2011). A new role of NUAK1: directly phosphorylating p53 and regulating cell proliferation. Oncogene *30*, 2933–2942.

Hsu, T.Y., Simon, L.M., Neill, N.J., Marcotte, R., Sayad, A., Bland, C.S., Echeverria, G.V., Sun, T., Kurley, S.J., Tyagi, S., et al. (2015). The spliceosome is a therapeutic vulnerability in MYC-driven cancer. Nature *525*, 384–388.

Huang, C.H., Lujambio, A., Zuber, J., Tschaharganeh, D.F., Doran, M.G., Evans, M.J., Kitzing, T., Zhu, N., de Stanchina, E., Sawyers, C.L., et al. (2014). CDK9-mediated transcription elongation is required for MYC addiction in hepatocellular carcinoma. Genes Dev. *28*, 1800–1814.

Humphrey, S.J., Azimifar, S.B., and Mann, M. (2015). High-throughput phosphoproteomics reveals in vivo insulin signaling dynamics. Nat. Biotechnol. *33*, 990–995.

Iwai, K., Yaguchi, M., Nishimura, K., Yamamoto, Y., Tamura, T., Nakata, D., Dairiki, R., Kawakita, Y., Mizojiri, R., Ito, Y., et al. (2018). Anti-tumor efficacy of a novel CLK inhibitor via targeting RNA splicing and MYC-dependent vulnerability. EMBO Mol. Med. *10*, e8289.

Kaida, D., Motoyoshi, H., Tashiro, E., Nojima, T., Hagiwara, M., Ishigami, K., Watanabe, H., Kitahara, T., Yoshida, T., Nakajima, H., et al. (2007).

Spliceostatin A targets SF3b and inhibits both splicing and nuclear retention of pre-mRNA. Nat. Chem. Biol. *3*, 576–583.

Kalkat, M., Resetca, D., Lourenco, C., Chan, P.K., Wei, Y., Shiah, Y.J., Vitkin, N., Tong, Y., Sunnerhagen, M., Done, S.J., et al. (2018). MYC protein interactome profiling reveals functionally distinct regions that cooperate to drive tumorigenesis. Mol. Cell *72*, 836–848.e7.

Kecman, T., Kus, K., Heo, D.H., Duckett, K., Birot, A., Liberatori, S., Mohammed, S., Geis-Asteggiante, L., Robinson, C.V., and Vasiljeva, L. (2018). Elongation/termination factor exchange mediated by PP1 phosphatase orchestrates transcription termination. Cell Rep. *25*, 259–269.e5.

Kfoury, A., Armaro, M., Collodet, C., Sordet-Dessimoz, J., Giner, M.P., Christen, S., Moco, S., Leleu, M., de Leval, L., Koch, U., et al. (2018). AMPK promotes survival of c-Myc-positive melanoma cells by suppressing oxidative stress. EMBO J. *37*, e97673.

Kim, Y.M., Watanabe, T., Allen, P.B., Kim, Y.M., Lee, S.J., Greengard, P., Nairn, A.C., and Kwon, Y.G. (2003). PNUTS, a protein phosphatase 1 (PP1) nuclear targeting subunit. Characterization of its PP1- and RNA-binding domains and regulation by phosphorylation. J. Biol. Chem. *278*, 13819–13828.

Kim, D., Pertea, G., Trapnell, C., Pimentel, H., Kelley, R., and Salzberg, S.L. (2013). TopHat2: accurate alignment of transcriptomes in the presence of insertions, deletions and gene fusions. Genome Biol. *14*, R36.

Koh, C.M., Bezzi, M., Low, D.H., Ang, W.X., Teo, S.X., Gay, F.P., Al-Haddawi, M., Tan, S.Y., Osato, M., Sabò, A., et al. (2015). MYC regulates the core pre-mRNA splicing machinery as an essential step in lymphomagenesis. Nature *523*, 96–100.

Kotake, Y., Sagane, K., Owa, T., Mimori-Kiyosue, Y., Shimizu, H., Uesugi, M., Ishihama, Y., Iwata, M., and Mizui, Y. (2007). Splicing factor SF3b as a target of the antitumor natural product pladienolide. Nat. Chem. Biol. *3*, 570–575.

Kress, T.R., Sabò, A., and Amati, B. (2015). MYC: connecting selective transcriptional control to global RNA production. Nat. Rev. Cancer *15*, 593–607.

Langmead, B., and Salzberg, S.L. (2012). Fast gapped-read alignment with Bowtie 2. Nat. Methods *9*, 357–359.

Langmead, B., Trapnell, C., Pop, M., and Salzberg, S.L. (2009). Ultrafast and memory-efficient alignment of short DNA sequences to the human genome. Genome Biol. *10*, R25.

Lee, J.H., You, J., Dobrota, E., and Skalnik, D.G. (2010). Identification and characterization of a novel human PP1 phosphatase complex. J. Biol. Chem. *285*, 24466–24476.

Li, H., Handsaker, B., Wysoker, A., Fennell, T., Ruan, J., Homer, N., Marth, G., Abecasis, G., and Durbin, R.; 1000 Genome Project Data Processing Subgroup (2009). The Sequence Alignment/Map format and SAMtools. Bioinformatics *25*, 2078–2079.

Liberzon, A., Subramanian, A., Pinchback, R., Thorvaldsdóttir, H., Tamayo, P., and Mesirov, J.P. (2011). Molecular signatures database (MSigDB) 3.0. Bioinformatics *27*, 1739–1740.

Liu, L., Ulbrich, J., Müller, J., Wüstefeld, T., Aeberhard, L., Kress, T.R., Muthalagu, N., Rycak, L., Rudalska, R., Moll, R., et al. (2012). Deregulated MYC expression induces dependence upon AMPK-related kinase 5. Nature *483*, 608–612.

Lowe, S.W., Cepero, E., and Evan, G. (2004). Intrinsic tumour suppression. Nature *432*, 307–315.

Matsumura, F., and Hartshorne, D.J. (2008). Myosin phosphatase target subunit: many roles in cell function. Biochem. Biophys. Res. Commun. *369*, 149–156.

Meerbrey, K.L., Hu, G., Kessler, J.D., Roarty, K., Li, M.Z., Fang, J.E., Herschkowitz, J.I., Burrows, A.E., Ciccia, A., Sun, T., et al. (2011). The pINDUCER lentiviral toolkit for inducible RNA interference in vitro and in vivo. Proc. Natl. Acad. Sci. U S A *108*, 3665–3670.

Monteverde, T., Tait-Mulder, J., Hedley, A., Knight, J.R., Sansom, O.J., and Murphy, D.J. (2018). Calcium signalling links MYC to NUAK1. Oncogene *37*, 982–992.

Muthalagu, N., Junttila, M.R., Wiese, K.E., Wolf, E., Morton, J., Bauer, B., Evan, G.I., Eilers, M., and Murphy, D.J. (2014). BIM is the primary mediator of MYC-induced apoptosis in multiple solid tissues. Cell Rep. *8*, 1347–1353.

Nguyen, H.D., Leong, W.Y., Li, W., Reddy, P.N.G., Sullivan, J.D., Walter, M.J., Zou, L., and Graubert, T.A. (2018). Spliceosome mutations induce R loop-associated sensitivity to ATR inhibition in myelodysplastic syndromes. Cancer Res. *78*, 5363–5374.

Nojima, T., Gomes, T., Grosso, A.R.F., Kimura, H., Dye, M.J., Dhir, S., Carmo-Fonseca, M., and Proudfoot, N.J. (2015). Mammalian NET-seq reveals genome-wide nascent transcription coupled to RNA processing. Cell *161*, 526–540.

Nojima, T., Rebelo, K., Gomes, T., Grosso, A.R., Proudfoot, N.J., and Carmo-Fonseca, M. (2018). RNA polymerase II phosphorylated on CTD serine 5 interacts with the spliceosome during co-transcriptional splicing. Mol. Cell *72*, 369–379.e4.

Olson, C.M., Jiang, B., Erb, M.A., Liang, Y., Doctor, Z.M., Zhang, Z., Zhang, T., Kwiatkowski, N., Boukhali, M., Green, J.L., et al. (2018). Pharmacological perturbation of CDK9 using selective CDK9 inhibition or degradation. Nat. Chem. Biol. *14*, 163–170.

Orchard, S., Ammari, M., Aranda, B., Breuza, L., Briganti, L., Broackes-Carter, F., Campbell, N.H., Chavali, G., Chen, C., del-Toro, N., et al. (2014). The MIntAct project–IntAct as a common curation platform for 11 molecular interaction databases. Nucleic Acids Res. D358–D363.

Palma, M., Riffo, E.N., Suganuma, T., Washburn, M.P., Workman, J.L., Pincheira, R., and Castro, A.F. (2019). Identification of a nuclear localization signal and importin beta members mediating NUAK1 nuclear import inhibited by oxidative stress. J. Cell. Biochem. *120*, 16088–16107.

Parua, P.K., Booth, G.T., Sansó, M., Benjamin, B., Tanny, J.C., Lis, J.T., and Fisher, R.P. (2018). A Cdk9-PP1 switch regulates the elongation-termination transition of RNA polymerase II. Nature *558*, 460–464.

Pelengaris, S., Khan, M., and Evan, G. (2002). c-MYC: more than just a matter of life and death. Nat. Rev. Cancer *2*, 764–776.

Port, J., Muthalagu, N., Raja, M., Ceteci, F., Monteverde, T., Kruspig, B., Hedley, A., Kalna, G., Lilla, S., Neilson, L., et al. (2018). Colorectal tumors require NUAK1 for protection from oxidative stress. Cancer Discov. *8*, 632–647.

Quinlan, A.R. (2014). BEDTools: the Swiss-Army tool for genome feature analysis. Curr. Protoc. Bioinformatics *47*, 11.12.1–11.12.34.

R Core Team (2013). R: A language and environment for statistical computing. R Foundation for Statistical Computing.

R Core Team (2017). R: A Language and Environment for Statistical Computing (Vienna, Austria: R Foundation for Statistical Computing).

Rahl, P.B., Lin, C.Y., Seila, A.C., Flynn, R.A., McCuine, S., Burge, C.B., Sharp, P.A., and Young, R.A. (2010). c-Myc regulates transcriptional pause release. Cell *141*, 432–445.

Ramírez, F., Ryan, D.P., Grüning, B., Bhardwaj, V., Kilpert, F., Richter, A.S., Heyne, S., Dündar, F., and Manke, T. (2016). deepTools2: a next generation web server for deep-sequencing data analysis. Nucleic Acids Res. *44* (W1), W160–W165.

Rappsilber, J., Ishihama, Y., and Mann, M. (2003). Stop and go extraction tips for matrix-assisted laser desorption/ionization, nanoelectrospray, and LC/MS sample pretreatment in proteomics. Anal. Chem. *75*, 663–670.

Ruprecht, B., Koch, H., Medard, G., Mundt, M., Kuster, B., and Lemeer, S. (2015). Comprehensive and reproducible phosphopeptide enrichment using iron immobilized metal ion affinity chromatography (Fe-IMAC) columns. Mol. Cell. Proteomics *14*, 205–215.

Ruprecht, B., Zecha, J., Zolg, D.P., and Kuster, B. (2017). High pH reversed-phase micro-columns for simple, sensitive, and efficient fractionation of proteome and (TMT labeled) phosphoproteome digests. Methods Mol. Biol. *1550*, 83–98.

Saldanha, A.J. (2004). Java Treeview—extensible visualization of microarray data. Bioinformatics *20*, 3246–3248.

Sansó, M., Levin, R.S., Lipp, J.J., Wang, V.Y., Greifenberg, A.K., Quezada, E.M., Ali, A., Ghosh, A., Larochelle, S., Rana, T.M., et al. (2016). P-TEFb regulation of transcription termination factor Xrn2 revealed by a chemical genetic screen for Cdk9 substrates. Genes Dev. *30*, 117–131.

Schneider, C.A., Rasband, W.S., and Eliceiri, K.W. (2012). NIH Image to ImageJ: 25 years of image analysis. Nat. Methods *9*, 671–675.

Shen, L., Shao, N., Liu, X., and Nestler, E. (2014). ngs.plot: quick mining and visualization of next-generation sequencing data by integrating genomic databases. BMC Genomics *15*, 284.

Shi, Y., Reddy, B., and Manley, J.L. (2006). PP1/PP2A phosphatases are required for the second step of Pre-mRNA splicing and target specific snRNP proteins. Mol. Cell *23*, 819–829.

Subramanian, A., Tamayo, P., Mootha, V.K., Mukherjee, S., Ebert, B.L., Gillette, M.A., Paulovich, A., Pomeroy, S.L., Golub, T.R., Lander, E.S., and Mesirov, J.P. (2005). Gene set enrichment analysis: a knowledge-based approach for interpreting genome-wide expression profiles. Proc. Natl. Acad. Sci. U S A *102*, 15545–15550.

Tanuma, N., Kim, S.E., Beullens, M., Tsubaki, Y., Mitsuhashi, S., Nomura, M., Kawamura, T., Isono, K., Koseki, H., Sato, M., et al. (2008). Nuclear inhibitor of protein phosphatase-1 (NIPP1) directs protein phosphatase-1 (PP1) to dephosphorylate the U2 small nuclear ribonucleoprotein particle (snRNP) component, spliceosome-associated protein 155 (Sap155). J. Biol. Chem. *283*, 35805–35814.

The Gene Ontology Consortium (2019). The Gene Ontology Resource: 20 years and still GOing strong. Nucleic Acids Res. *47*, D330–D338.

Tyanova, S., Temu, T., and Cox, J. (2016). The MaxQuant computational platform for mass spectrometry-based shotgun proteomics. Nat. Protoc. *11*, 2301–2319.

Verbinnen, I., Ferreira, M., and Bollen, M. (2017). Biogenesis and activity regulation of protein phosphatase 1. Biochem. Soc. Trans. *45*, 89–99.

Verheyen, T., Görnemann, J., Verbinnen, I., Boens, S., Beullens, M., Van Eynde, A., and Bollen, M. (2015). Genome-wide promoter binding profiling of protein phosphatase-1 and its major nuclear targeting subunits. Nucleic Acids Res. *43*, 5771–5784.

Walz, S., Lorenzin, F., Morton, J., Wiese, K.E., von Eyss, B., Herold, S., Rycak, L., Dumay-Odelot, H., Karim, S., Bartkuhn, M., et al. (2014). Activation and repression by oncogenic MYC shape tumour-specific gene expression profiles. Nature *511*, 483–487.

Werle, K., Chen, J., Xu, H.G., Zhao, R.X., He, Q., Lu, C., Cui, R., Liang, J., Li, Y.L., and Xu, Z.X. (2014). Liver kinase B1 regulates the centrosome via PLK1. Cell Death Dis. *5*, e1157.

Wickham, H. (2016). ggplot2: Elegant Graphics for Data Analysis (New York: Springer-Verlag).

Xiang, Y., Stine, Z.E., Xia, J., Lu, Y., O'Connor, R.S., Altman, B.J., Hsieh, A.L., Gouw, A.M., Thomas, A.G., Gao, P., et al. (2015). Targeted inhibition of tumor-specific glutaminase diminishes cell-autonomous tumorigenesis. J. Clin. Invest. *125*, 2293–2306.

Xing, Z., Lin, A., Li, C., Liang, K., Wang, S., Liu, Y., Park, P.K., Qin, L., Wei, Y., Hawke, D.H., et al. (2014). lncRNA directs cooperative epigenetic regulation downstream of chemokine signals. Cell *159*, 1110–1125.

Zagórska, A., Deak, M., Campbell, D.G., Banerjee, S., Hirano, M., Aizawa, S., Prescott, A.R., and Alessi, D.R. (2010). New roles for the LKB1-NUAK pathway in controlling myosin phosphatase complexes and cell adhesion. Sci. Signal. *3*, ra25.

Zecha, J., Satpathy, S., Kanashova, T., Avanessian, S.C., Kane, M.H., Clauser, K.R., Mertins, P., Carr, S.A., and Kuster, B. (2019). TMT labeling for the masses: a robust and cost-efficient, in-solution labeling approach. Mol. Cell. Proteomics *18*, 1468–1478.

Zhang, Y., Liu, T., Meyer, C.A., Eeckhoute, J., Johnson, D.S., Bernstein, B.E., Nusbaum, C., Myers, R.M., Brown, M., Li, W., and Liu, X.S. (2008). Model-based analysis of ChIP-seq (MACS). Genome Biol. *9*, R137.

STAR★METHODS

KEY RESOURCES TABLE

REAGENT or RESOURCE	SOURCE	IDENTIFIER
Antibodies		
Mouse monoclonal anti-ACTB	Sigma-Aldrich	Cat #A5441 RRID:AB_476744 Lot: 026M4780V
Mouse monoclonal anti-VCL	Sigma-Aldrich	Cat #V9131 RRID:AB_477629 Lot: 036M4797V
Rabbit polyclonal anti-NUAK1 (used for immunofluorescence)	Sigma-Aldrich	Cat# HPA057143 RRID:AB_2683349 Lot: R80360
Mouse monoclonal anti-FLAG M2	Sigma-Aldrich	Cat #F3165 RRID:AB_259529 Lot: SLBT6752
Mouse monoclonal anti-HA-tag (used for immunofluorescence)	Abcam	Cat# ab130275 RRID:AB_11156884 Lot: GR320538-5
Rabbit polyclonal anti-HA-tag (used for ChIP)	Abcam	Cat# ab9110 RRID:AB_307019) Lot: GR3177614-4
Rabbit monoclonal anti-MYC (clone Y69)	Abcam	Cat# ab32072 RRID:AB_731658 Lot: GR295111-34
Rabbit polyclonal anti-phospho-Ser2-RNAPII	Abcam	Cat# ab5095 RRID:AB_304749 Lot: GR3231908-1
Rabbit polyclonal anti-TUBA1A	Santa Cruz Biotechnology	Cat# sc-12462 RRID:AB_2241125
Mouse monoclonal anti-PNUTS (used for immunoblotting)	Santa Cruz Biotechnology	Cat# sc-271681 RRID:AB_10708580 Lot: K1913
Mouse monoclonal anti-PPP1CB	Santa Cruz Biotechnology	Cat# sc-373782 RRID:AB_10916703
Rabbit monoclonal anti-phospho-Thr313-SF3B1	Cell Signaling Technology	Cat# 25009 RRID:AB_2798893 Lot: 1
Rabbit monoclonal anti-HA-tag (used for immunoblotting)	Cell Signaling Technology	Cat# 3724 RRID:AB_1549585 Lot: 7
Rabbit monoclonal anti-MYPT1	Cell Signaling Technology	Cat# 8574 RRID:AB_10998518 Lot: 1
Rabbit polyclonal anti-NUAK1 (used for immunoblotting)	Cell Signaling Technology	Cat# 4458 RRID:AB_2155859 Lot: 3
Rabbit polyclonal anti-PNUTS (used for ChIP, immunofluorescence and immunoblotting)	Bethyl Laboratories	Cat# A300-439A RRID:AB_420948 Lot: 439A-3
Mouse monoclonal anti-SF3B1	MBL International	Cat# D221-3 RRID:AB_592712
Mouse monoclonal anti-RNAPII	MBL International	Cat# MABI0601 RRID:AB_2728735
Mouse monoclonal anti-phospho-Ser5-RNAPII	BioLegend	Cat# 904001 RRID:AB_2565036
Mouse monoclonal anti-RNA-DNA hybrid (clone S9.6)	Kerafast	Cat# ENH001 RRID:AB_2687463
Mouse monoclonal anti-SC35	BD PharMingen	Cat# 556363 RRID:AB_396388
Rabbit polyclonal anti-phospho-Thr328-SF3B1	Lührmann lab	Girard et al., 2012
Rabbit polyclonal anti-phospho-Thr313-SF3B1	Lührmann lab	Girard et al., 2012
Rabbit polyclonal anti-phospho-Ser313-PNUTS	Raised for this study (Davids Biotechnologie)	N/A
IRDye 800CW Donkey anti-Rabbit IgG (H + L)	LI-COR Biosciences	Cat# 926-32213 RRID:AB_621848
IRDye 680RD Donkey anti-Mouse IgG (H + L)	LI-COR Biosciences	Cat# 926-68072 RRID:AB_10953628
Goat anti-Rabbit IgG (H+L) Highly Cross-Adsorbed Secondary Antibody, Alexa Fluor 488	Thermo Fisher Scientific	Cat# A-11008 RRID:AB_143165
Goat anti-Mouse IgG (H+L) Highly Cross-Adsorbed Secondary Antibody, Alexa Fluor 568	Thermo Fisher Scientific	Cat# A-11004 RRID:AB_2534072
Bacterial and Virus Strains		
pBABE-puro (empty vector)	This study	N/A
pBABE-Nuak1-FLAG-NT-puro	This study	N/A
pBABE-Nuak1-FLAG-CT-puro	This study	N/A
pBABE-Nuak1-HA-NT-puro	This study	N/A
pBABE-Nuak1-HA-CT-puro	This study	N/A
pINDUCER11-NUAK1#1	This study	N/A

(*Continued on next page*)

Continued

REAGENT or RESOURCE	SOURCE	IDENTIFIER
pINDUCER11-NUAK1#2	This study	N/A
pINDUCER11-NUAK1#3	This study	N/A
**Chemicals, Peptides, and Recombinant Proteins**		
Stearyl-R8-KRPKGILKKRS peptide	LifeTein	N/A
FITC-Stearyl-R8 peptide	LifeTein	Cat# LT12013
BAY-880, IUPAC name 7-cyclopentyl-5-ethyl-2-(1H-pyrrol-2-yl)imidazo[5,1-f][1,2,4]triazin-4(3H)-one	Bayer	European Patent EP1399439B1, example 43
NUAK1i (structurally related to BAY-880)	Bayer	N/A
Calyculin A	Santa Cruz Biotechnology	Cat# sc-24000
Pladienolide B	Santa Cruz Biotechnology	Cat# sc-391691
HTH-01-015	Selleckchem	Cat# S7318
WZ4003	Selleckchem	Cat# S7317
P276-00	Selleckchem	Cat# S8058
LDC000067	Selleckchem	Cat# S7461
NVP-2	Tocris/Bio-Techne	Cat# 6535/5
4-Thiouridine (4sU)	Sigma-Aldrich	Cat# T4509
BX795	Sigma-Aldrich	Cat# 204001
Okadaic acid	Sigma-Aldrich	Cat# 459620
Flavopiridol	Sigma-Aldrich	Cat# F3055
4-hydroxytamoxifen	Sigma-Aldrich	Cat# H7904
Doxycycline	Sigma-Aldrich	Cat# D9891
Cholera toxin	Sigma-Aldrich	Cat# C8052
Insulin	Sigma-Aldrich	Cat# I9278
Hydrocortisone	Sigma-Aldrich	Cat# H0396
Protease inhibitor cocktail	Sigma-Aldrich	Cat# P8340
Phosphatase inhibitor cocktail 2	Sigma-Aldrich	Cat# P5726
Phosphatase inhibitor cocktail 3	Sigma-Aldrich	Cat# P0044
Duolink *In Situ* PLA Probe Anti-Rabbit PLUS	Sigma-Aldrich	Cat# DUO92002
Duolink *In Situ* PLA Probe Anti-Mouse MINUS	Sigma-Aldrich	Cat# DUO92004
Duolink *In Situ* Detection Reagents Red	Sigma-Aldrich	Cat# DUO92008
Puromycin	InvivoGen	Cat# ant-pr-1
Benzonase	Merck Millipore	Cat#70664-3
DAPI	Roth	Cat# 6335.1
RNase A	Roth	Cat# 7156.1
RNase H	NEB	Cat# M0297
EcoRI	NEB	Cat# R0101
BamHI	NEB	Cat# R0136
BsrGI	NEB	Cat# R0575
HindIII	NEB	Cat# R0104
SspI	NEB	Cat# R0132
XbaI	NEB	Cat# R0145
XhoI	NEB	Cat# R0146
Dynabead Protein A	Thermo Fisher Scientific	Cat# 10002D
Dynabead Protein G	Thermo Fisher Scientific	Cat# 10004D
Dynabeads MyOne Streptavidin T1	Thermo Fisher Scientific	Cat# 65601
Dithiothreitol (DTT)	Thermo Fisher Scientific	Cat# 20291
Lipofectamine 2000	Thermo Fisher Scientific	Cat# 11668019

(*Continued on next page*)

Continued		
REAGENT or RESOURCE	SOURCE	IDENTIFIER
Lipofectamine RNAiMAX	Thermo Fisher Scientific	Cat# 13778-150
ibidi Mounting Medium	Ibidi	Cat# 50001
Odyssey Blocking Buffer in TBS	LI-COR Biosciences	Cat# 927-50000
peqGOLD Trifast	PeqLab/WVR	Cat# 30-2010
Critical Commercial Assays		
Quant-iT PicoGreen dsDNA assay	Thermo Fisher Scientific	Cat# P7589
Quant-iT RiboGreen RNA Assay Kit	Thermo Fisher Scientific	Cat# R11490
NEBNext ChIP-Seq Library Prep Master Mix Set for Illumina	NEB	Cat# E6240
NEBNext Ultra Directional RNA Library Prep Kit for Illumina	NEB	Cat# E7420
NEBNext Ultra II RNA Library Prep Kit for Illumina	NEB	Cat# E7770
NEBNext rRNA Depletion Kit	NEB	Cat#E6310
NEBNext Ultra II RNA Library Prep Kit for Illumina	NEB	Cat# E7770
RNeasy MinElute Cleanup Kit	QIAGEN	Cat#74204
miRNeasy Mini Kit	QIAGEN	Cat# 217004
NextSeq 500/550 High Output Kit v2	Illumina	Cat# FC-404-2005
Deposited Data		
Raw and analyzed data	This study	GEO: GSE129925
Raw and analyzed data	Walz et al., 2014	GEO: GSE44672
Raw and analyzed data	De Magis et al., 2019	GEO: GSE115957
Raw and analyzed data	Olson et al., 2018	GEO: GSE89384
Human reference genome GRCh37/hg19	Genome Reference Consortium	https://support.illumina.com/sequencing/sequencing_software/igenome.html
Raw images, proteomic data	This study	Mendeley Data https://dx.doi.org/10.17632/rm56h9msym.1
Experimental Models: Cell Lines		
U2OS	ATCC	RRID: CVCL_0042
U2OS MYC-ER	Liu et al., 2012	N/A
HEK293TN	ATCC	RRID: CVCL_UL49
PlatE	ATCC	RRID: CVCL_B488
HeLa	ATCC	RRID: CVCL_0030
KPC	Siveke lab	N/A
IMR5	Eggert lab	RRID: CVCL_1306
NGP	Eggert lab	RRID: CVCL_2141
SH-SY5Y	ATCC	RRID: CVCL_0019
Kelly	Eggert lab	RRID: CVCL_2092
MCF10A	ATCC	RRID: CVCL_0598
SKNAS	ATCC	RRID: CVCL_1700
NIH 3T3	ATCC	RRID: CVCL_0594
Oligonucleotides		
Primers for ChIP, DRIP and qPCR: Table S2	This study	N/A
Primers for cloning: Table S3	This study	N/A
Primers for DRIP: Table S4	This study	N/A
mirE_shNUAK1#1: TGCTGTTGACAGTGAGCGC ACGGTGGATGCTGATGGTGAATAGTGAAG CCACAGATGTATTCACCATCAGCATCC ACCGTATGCCTACTGCCTCGGA	This study	N/A

(*Continued on next page*)

Continued

REAGENT or RESOURCE	SOURCE	IDENTIFIER
mirE_shNUAK1#2: TGCTGTTGACAGTGAG CGAGCTGAAGAAATCCAAGAAAGATAG TGAAGCCACAGATGTATCTTTCTTGGA TTTCTTCAGCGTGCCTACTGCCTCGGA	This study	N/A
mirE_shNUAK1#3: TGCTGTTGACAGTG AGCGATAGGGATTTACTGGCATGGTATA GTGAAGCCACAGATGTATACCATGCCAG TAAATCCCTACTGCCTACTGCCTCGGA	This study	N/A
ON-TARGETplus Non-targeting Pool	Dharmacon / Horizon Discovery	Cat# D-001810-10-50
ON-TARGETplus Human NUAK1 siRNA - SMARTpool	Dharmacon / Horizon Discovery	Cat# L-004931-01-0005
ON-TARGETplus Human PPP1R10 siRNA – SMARTpool	Dharmacon / Horizon Discovery	Cat# L-011358-00-0005
PPP1R10 Silencer Select siRNA	Thermo Fisher Scientific	Cat# 4392420 - s328
Recombinant DNA		
pBABE-puro	Liu et al., 2012	N/A
pBABE-Nuak1-FLAG-NT-puro	This study	N/A
pBABE-Nuak1-FLAG-CT-puro	This study	N/A
pBABE-Nuak1-HA-NT-puro	This study	N/A
pBABE-Nuak1-HA-CT-puro	This study	N/A
pcDNA3	Thermo Fisher Scientific	Cat# V79020
pcDNA5/TO-Flag-mPNUTS	Skalnik Lab	Lee et al., 2010
pcDNA3-Ppp1r10-HA-wt-puro	This study	N/A
pcDNA3-Ppp1r10-HA-S313A-puro	This study	N/A
pcDNA3-Ppp1r10-HA-S313D-puro	This study	N/A
pcDNA3-Ppp1r10-HA-S313E-puro	This study	N/A
pINDUCER11	Trono lab	Meerbrey et al., 2011
pINDUCER11-NUAK1#1	This study	N/A
pINDUCER11-NUAK1#2	This study	N/A
pINDUCER11-NUAK1#3	This study	N/A
psPAX2	Addgene	Cat# 12260
pMD2.G	Addgene	Cat# 12259
Software and Algorithms		
Max Quant	Cox and Mann, 2008	http://www.coxdocs.org/doku.php?id=:maxquant:start
Spotfire	TIBCO	N/A
Image Studio Lite v5.2.5	LI-COR Biosciences	N/A
ImageJ v1.49	Schneider et al., 2012	https://imagej.net/ImageJ
Prism v6	GraphPad	N/A
Integrated Genome Browser v9.0.2	Freese et al., 2016	https://bioviz.org/
Bowtie v2.3.5	Langmead et al., 2009	http://bowtie-bio.sourceforge.net/index.shtml
TopHat v2.1.1	Kim et al., 2013	https://ccb.jhu.edu/software/tophat/index.shtml
Bedtools v2.26.0	Quinlan, 2014	https://github.com/arq5x/bedtools2/releases
SAMtools v1.3	Li et al., 2009	http://samtools.sourceforge.net
DeepTools v2.3.5-3-2c5f94d	Ramírez et al., 2016	https://deeptools.readthedocs.io/en/develop/index.html
ngsPlot v2.61	Shen et al., 2014	https://github.com/shenlab-sinai/ngsplot/
R (v 3.4.4 or 3.5.1)	R Core Team, 2017	https://www.r-project.org/
TreeView 1.16r4	Saldanha, 2004	http://jtreeview.sourceforge.net/

(*Continued on next page*)

Continued

REAGENT or RESOURCE	SOURCE	IDENTIFIER
FastQC v0.11.5	N/A	http://www.bioinformatics.babraham.ac.uk/projects/fastqc/
GSEA v2.2	Subramanian et al., 2005	http://software.broadinstitute.org/gsea/index.jsp
MSigDB database v6.0	Liberzon et al., 2011	http://software.broadinstitute.org/gsea/msigdb/index.jsp
macs v1.4.1	Zhang et al., 2008	https://taoliu.github.io/MACS/
AmiGO v2	Carbon et al., 2009	http://amigo.geneontology.org/amigo
Perseus v1.6.2.3	Tyanova et al., 2016	https://maxquant.net/perseus/

LEAD CONTACT AND MATERIALS AVAILABILITY

Further information and requests for resources and reagents should be directed to and will be fulfilled by the Lead Contact, Martin Eilers (martin.eilers@biozentrum.uni-wuerzburg.de). There are restrictions to the availability of BAY-880 due to the lack of an external centralized repository for its distribution and our need to maintain the stock. All other unique reagents generated in this study are available from the Lead Contact without restriction.

EXPERIMENTAL MODEL AND SUBJECT DETAILS

Employed cell lines

U2OS, HeLa, HEK293TN and PlatE human female; KPC and NIH 3T3 mouse male cell lines were cultured at 37°C (5% CO2) in DMEM (Thermo Fisher Scientific) supplemented with 10% fetal bovine serum (FBS, Sigma-Aldrich). SKNAS, SH-SY5Y, Kelly (female) and NGP, IMR-5 (male) human neuroblastoma cell lines were grown in RPMI-1640 (Thermo Fisher Scientific) supplemented with 10% fetal bovine serum. MCF10A female human cell line was cultured in DMEM/F-12 (Thermo Fisher Scientific) supplemented with 5% horse serum (Sigma-Aldrich), 100 μg/ml cholera toxin, 10 μg/ml insulin, 0.5 μg/ml hydrocortisone and 20 ng/ml EGF. All cell lines were verified by single tandem repeat profiling and routinely tested for mycoplasma contamination.

Cell culture treatments

For siRNA transfection, 10 μl of 20 nM siRNA were mixed with 10 μl Lipofectamine RNAiMAX in 1 mL OptiMEM (Thermo Fisher Scientific). After 5 min incubation at RT, the mixture was added to cells overnight.

For plasmid transfection, FBS concentration was first adjusted to 2%. 10 μg DNA were then mixed with 10 μl Lipofectamine or polyethylenimine (PEI, Sigma-Aldrich) in 1 mL OptiMEM. After 20 min incubation at RT, the mixture was added to cells overnight.

For retroviral infection, PlatE cells were cultured till 80% confluence, then the FBS concentration was adjusted to 2%. 30 μg DNA were then mixed with 24 μl PEI in 1 mL OptiMEM. After 20 min incubation, the mixture was added to cells overnight. Cells were then cultured with 10% FBS for two days, collecting virus-containing supernatant every 24 h. Dead cells were removed from supernatant by employing 0.45 μm filters. Target cells were then cultured to 80% confluence and added 3 mL supernatant, 2 mL DMEM with 10% FBS and 4 μg/ml Polybrene (Sigma-Aldrich).

For lentiviral infection, HEK293TN cells were cultured to 80% confluence, then the FBS concentration was adjusted to 2%. 8 μg vector DNA, 8 μg psPAX2 and 2 μg pMD2.G were then mixed with 24 μl polyethylenimine in 1 mL OptiMEM. After 20 min incubation, the mixture was added to cells overnight. Cells were then cultured with 10% FBS for two days, collecting virus-containing supernatant every 12 h. Dead cells were removed from the supernatant by employing 0.45 μm filters. Target cells were then cultured to 30% confluence and added 500 μl supernatant, 5 mL DMEM with 10% FBS and 6 ug/ml Polybrene.

For peptide treatment, peptides were dissolved in water and added directly to cell culture medium. Peptides were designed with a stearyl-8xArg tail to facilitate cell penetration. GILK peptide sequence encompasses residues 395 to 407 within the GILK site #1 of NUAK1 (Zagórska et al., 2010).

BAY-880 – originally described in the European Patent EP 1399439B1 – was identified as a potent NUAK1 inhibitor by Bayer in an ultraHTS campaign screening 2.6 million compounds. The assay in the primary HTS used recombinant NUAK1 to phosphorylate a synthetic peptide. The phospho-readout was by TR-FRET. Primary hits were confirmed in an orthogonal ADP-Glo assay.

METHOD DETAILS

Immunofluorescence

Depending on the downstream application, cells were seeded in μ-Slide 8-well or 18-well chambers (Ibidi) or in 96-well μclear plates (Greiner). At the appropriate time point, cells were fixed with 4% PFA in PBS and permeabilized at least 20 min in ice-cold 100% methanol. Cells were then incubated at least 30 min in 5% BSA in PBS ("blocking solution"). First antibody in blocking solution

was then added overnight. Wells were washed 3x with PBS, then added 1:1000 Alexa Fluor-conjugated second antibody in blocking solution for 1 h. 1:1000 DAPI in blocking solution was then added to wells were then added of for 5 min and washed 3x with PBS. Wells were added of ibidi Mounting Medium or PBS and stored at 4°C up to one week. Proximity ligation assays (PLA) were carried out using the Duolink *In Situ* Kit according to the manufacturer's protocol. Imaging was performed with a Nikon Eclipse-Ti confocal microscope (equipped with the NIS-elements AR 3.22.15 software) or with the Operetta High-Content Imaging System (Perkin Elmer).

Cell fractionation

Plates were washed in ice-cold PBS (containing 1:1000 protease and phosphatase inhibitors) and cells were collected with a scraper and pelleted by 250 g centrifugation for 40 min. Pellets were resuspended in 1 mL sucrose buffer (10 mM HEPES pH 7.9, 0.34 M Sucrose, 3 mM $CaCl_2$, 2 M magnesium acetate, 0.1 mM EDTA) with 0.5% NP-40 and incubated on a rotator for 10 min. Nuclei were then pelleted by 3900 g centrifugation for 20 min. The supernatant was collected as cytoplasmic fraction, while pellets were washed in 1 mL sucrose buffer and pelleted again by 3900 g centrifugation for 20 min. Pelleted nuclei were resuspended in 1 mL nucleoplasmic extraction buffer (20mM HEPES pH 7.9, 3 mM EDTA, 10% glycerol, 150 mM potassium acetate, 1.5 mM $MgCl_2$), homogenized with a Dounce homogenizer and, after 40 min incubation, homogenized again. When applicable, samples were treated at this stage for 15 min with 20 μg RNase A at 37°C. Samples were then incubated 1 h with 25U benzonase and then centrifuged by 13000 rpm for 30 min. The supernatant was collected as nucleoplasmic fraction, while pellets were resuspended in 150 mM HEPES pH 7.9, 1.5 mM $MgCl_2$, 150 mM potassium acetate with 2.5 U benzonase. After incubation for 30 min on a rotating wheel at RT, samples were pelleted and supernatants were collected as chromatin fraction.

Immunoblotting

Whole-cell extracts were prepared using RIPA buffer (50 mM Tris pH 7.4, 150 mM NaCl, 0.1% SDS, 0.5% sodium deoxycholate, 1% NP-40). Briefly, plates were washed in ice-cold PBS (containing 1:1000 protease and phosphatase inhibitors) and cells were collected with a scraper and pelleted by 13000 rpm centrifugation for 5 min. Pellets were resuspended in RIPA buffer and incubated 30 min on ice. Tubes were then centrifuged at 13000 rpm for 5 min and supernatants were collected for further use. Protein lysates were quantified according to standard procedures (i.e., Bradford assay or bicinchoninic acid assay), separated employing Bis-Tris acrylamide gels and transferred on PVDF membranes (Merck-Millipore). Membranes were blocked 1 h with 20% Odyssey Blocking Buffer in TBS, incubated overnight with listed first antibodies in 20% Odyssey Blocking Buffer in TBS, washed 3x with TBS-T, incubated 1 h with IRDye 680RD or 800CW second antibodies, washed 3x with TBS-T and imaged and quantified with the Odyssey CLx Infrared Imaging System (LI-COR Biosciences).

Co-immunoprecipitation

Plates were washed in ice-cold PBS (containing 1:1000 protease and phosphatase inhibitors) and cells were collected with a scraper and pelleted by 1500 rpm centrifugation for 10 min. Pellets were lysed in HEGN buffer (20 mM HEPES-KOH pH 7.8, 0.2 mM EDTA, 0.1% NP-40, 10 mM sodium pyrophosphate, 140 mM KCl, 10% glycerol, containing 1:1000 protease and phosphatase inhibitors), sonicated with a Branson sonifier 4x5 s at 20% amplitude and incubated 30 min on ice. Lysates were cleared upon repeated centrifugations and quantified according to standard procedures (i.e., Bradford assay of bicinchoninic acid assay). 1%–2% lysate was kept as input control. Unless otherwise noted, lysates were added 1 μg antibody (including a sample with 1 μg IgG control antibody, Sigma-Aldrich) and incubated 3-6 h on a rotating wheel at 4°C. 7.5 μl protein A/G Dynabeads per immunoprecipitation were washed three times in HEGN, then incubated with the lysate overnight. Beads were washed three times with HEGN, then resuspended in 60 μl 1X Lämmli buffer and processed for immunoblotting.

Proteomic analysis of NUAK1 interactors

U2OS cells stably expressing N-terminal or C-terminal FLAG-tagged murine Nuak1 (or empty pBABE vector) were harvested and subjected to standard immunoprecipitation with minor modifications. Briefly, lysates were incubated overnight with anti-FLAG M2 magnetic beads (Sigma-Aldrich) and immunoprecipitates were eluted with 150ng/μl 3X FLAG peptide (Sigma-Aldrich) and acetone-precipitated. 5% of samples were loaded on Bis-Tris gels for checking actual FLAG-Nuak1 immunoprecipitation by immunoblotting or silver staining.

Acetone-precipitated samples were dissolved in NuPAGE LDS sample buffer (Thermo Fisher Scientific), reduced with 50 mM DTT at 70°C for 10 min and alkylated with 120 mM Iodoacetamide at room temperature for 20 min. Separation was performed on NuPAGE Novex 4%–12% Bis-Tris gels (Thermo Fisher Scientific) with MOPS buffer according to manufacturer's instructions. After washing 3x with water, gels were stained for 1h with Simply Blue Safe Stain (Thermo Fisher Scientific). After washing with water for 1 h, each gel lane was cut into 15 slices, destained with 30% acetonitrile in 0.1 M NH4HCO3 (pH 8), shrunk with 100% acetonitrile, and dried in a vacuum concentrator 5301 (Eppendorf). Samples were then digested with 0.1 μg trypsin per gel band overnight at 37°C in 0.1 M NH4HCO3 (pH 8). Peptides were extracted from the gel slices with 5% formic acid.

NanoLC-MS/MS analyses were performed on an LTQ-Orbitrap Velos Pro (Thermo Fisher Scientific) equipped with an EASY-Spray Ion Source and coupled to an EASY-nLC 1000 (Thermo Fisher Scientific). Peptides were loaded on a trapping column (2 cm x 75 μm ID. PepMap C18, 3 μm particles, 100 Å pore size) and separated on an EASY-Spray column (25 cm x 75 μm ID, PepMap C18, 2 μm

particles, 100 Å pore size) with a 30-minute linear gradient from 3% to 30% acetonitrile and 0.1% formic acid. MS scans were acquired in the Orbitrap analyzer with a resolution of 30,000 at m/z 400, MS/MS scans were acquired in the Orbitrap analyzer with a resolution of 7,500 at m/z 400 using HCD fragmentation with 30% normalized collision energy. A TOP5 data-dependent MS/MS method was used; dynamic exclusion was applied with a repeat count of 1 and an exclusion duration of 30 s; singly charged precursors were excluded from selection. Minimum signal threshold for precursor selection was set to 50,000. Predictive AGC was used with AGC target a value of 1e6 for MS scans and 5e4 for MS/MS scans. Lock mass option was applied for internal calibration in all runs using background ions from protonated decamethylcyclopentasiloxane (m/z 371.10124).

Raw MS data files were analyzed with MaxQuant version 1.6.2.2 (Cox and Mann 2008). Database search was performed with Andromeda, which is integrated in the utilized version of MaxQuant. The search was performed against the UniProt Human database. Additionally, a database containing common contaminants was used. The search was performed with tryptic cleavage specificity with 3 allowed miscleavages. Protein identification was under control of the false-discovery rate (1% FDR on protein and peptide level). In addition to MaxQuant default settings, the search was performed against following variable modifications: Protein N-terminal acetylation, Gln to pyro-Glu formation (N-term. Gln) and oxidation (Met). Carbamidomethyl (Cys) was set as fixed modification. For protein quantitation, the LFQ intensities were used (Cox et al., 2014). Proteins with less than two identified razor/unique peptides were dismissed.

Further data analysis was performed using R scripts developed in-house. LFQ intensities were used and missing LFQ intensities in the control samples were imputed with values close to the baseline. Data imputation was performed with values from a standard normal distribution with a mean of the 5% quantile of the combined log10-transformed LFQ intensities and a standard deviation of 0.1. For the identification of significantly co-immunoprecipitated proteins, mean log2 transformed protein ratio were calculated from the two replicate experiments and boxplot outliers were identified in intensity bins of at least 300 proteins. Log2 transformed protein ratios of CoIP versus control with values outside a 1.5x (potential) or 3x (extreme) interquartile range (IQR), respectively, were considered as significantly co-immunoprecipitated. GO term analyses of the dataset were performed with the web-available tool AmiGO (Carbon et al., 2009).

Label-free phosphoproteomics

U2OS cells were treated for 2 h with 10 μM BAY-880, 10 μM HTH-01-015 or DMSO, then plates were washed in ice-cold PBS (containing 1:1000 protease and phosphatase inhibitors). Cells were then collected with a scraper, pelleted by 1500 rpm centrifugation for 10 min and flash-frozen in liquid nitrogen.

Samples were then digested in trifluoroacetic acid (TFA) and phosphopeptide-enriched according to the EasyPhos protocol with some changes (Humphrey et al., 2015).

Pellets were first resuspended in GdmCl lysis buffer (6 M GdmCl, 100 mM Tris pH 8.5, 10 mM TCEP, 40 mM CAA). Samples were then added 50 mM DTT, shaken 10 min at 70°C, added 120 mM iodoacetamide and incubated 30 min in the dark. After adding 50 μl 1M ABC buffer, samples were added 4X acetone and proteins precipitated overnight. After centrifugation at 2000 g for 15 min, pellets were washed 3x with cold acetone and dried up. Pellets were then resuspended in 750 μl digestion buffer (10% TFA in 100mM ABC buffer) and sonified in a Bioruptor (5 cycles of 30sec). Samples were added 1:100 LysC and incubated 1 h at 37°C, then added 10 μg sequencing-grade modified trypsin (Promega) and incubated overnight at 37°C upon shaking. Digested samples were centrifuged at 16000 g for 20 min, then lyophilized overnight. Samples were dissolved in 2% ACN, 0.5% FA with the help 3x30 s Bioruptor cycles and 20 min shaking. Samples were then centrifuged for 5 min at 5000 g and cleaned up with Sep Pak C18 cartridges (Waters). 10 μl sample was collected for protein measurement, while the rest dried up lyophilized. Phosphopeptides were enriched with PhosphTio tips 3 mg/ 200 μl (GLSciences) and eluted in 150 μl 15% ACN, 5% ammoniumhydroxide before overnight lyophilization. Samples were redissolved in 100 μl 200 mM citric acid with 20 μl ACN and 1 μl formic acid shortly before NanoLC-MS/MS measurement.

NanoLC-MS/MS analyses were performed on an Orbitrap Fusion (Thermo Fisher Scientific) equipped with a PicoView Ion Source (New Objective) and coupled to an EASY-nLC 1000 (Thermo Fisher Scientific). Peptides were loaded on capillary columns (PicoFrit, 30 cm x 150 μm ID, New Objective) self-packed with ReproSil-Pur 120 C18-AQ, 1.9 μm (Dr. Maisch) and separated with a 90-minute linear gradient from 3% to 40% acetonitrile and 0.1% formic acid and a flow rate of 500 nl/min.

Both MS and MS/MS scans were acquired in the Orbitrap analyzer with a resolution of 60,000 for MS scans and 15,000 for MS/MS scans. HCD fragmentation with 35% normalized collision energy was applied. A Top Speed data-dependent MS/MS method with a fixed cycle time of 3 s was used. Dynamic exclusion was applied with a repeat count of 1 and an exclusion duration of 45 s; singly charged precursors were excluded from selection. Minimum signal threshold for precursor selection was set to 50,000. Predictive AGC was used with AGC a target value of 2e5 for MS scans and 5e4 for MS/MS scans. EASY-IC was used for internal calibration.

Raw MS data files were analyzed with MaxQuant version 1.5.7.4. Database search was performed with Andromeda, which is integrated in the utilized version of MaxQuant. The search was performed against the UniProt Human database. Additionally, a database containing common contaminants was used. The search was performed with tryptic cleavage specificity with 3 allowed miscleavages. Protein identification was under control of the false-discovery rate (1% FDR on protein and peptide level). In addition to MaxQuant default settings, the search was performed against following variable modifications: oxidation (Met), Gln to pyro-Glu formation (N-term. Gln) and Phospho (STY). Carbamidomethyl (Cys) was set as fixed modification. For protein quantitation, the LFQ intensities were used (Cox et al., 2014). Proteins with less than two identified razor/unique peptides were dismissed. Intensities from MaxQuant Phospho (STY) table were used for relative quantitation of phosphorylation sites.

Spike-in SILAC phosphoproteomics

U2OS cells were transfected overnight with siRNA targeting NUAK1 and grown for further 48 h after transfection start in standard DMEM. In parallel, SILAC reference cells were cultured with SILAC media (DMEM (-Lys, Arg, Gln), 10% dialyzed FBS, 1X LysH, 1XArgH, 1XGlnH) and grown until complete heavy label incorporation (> 98%). At the moment of harvesting, cells were then collected with a scraper, pelleted by 1500 rpm centrifugation for 10 min and flash-frozen in liquid nitrogen.

Cell pellets were lysed with 8 M urea, 2 mM EDTA in 50 mM Tris HCl buffer, containing protease and phosphatase inhibitor cocktails. Cells were sonicated for 20 s with 2 pulses at 60% power (Sonoplus HD2070, Bandelin, Germany).

Protein concentration was determined by Bradford colorimetric assay. 1.75 mg of each sample was mixed with an equal amount of SILAC reference sample and proteins were reduced in DTT 2 mM for 30 min at 25°C and successively free cysteines were alkylated in 11 mM iodoacetamide for 20 min at room temperature in the darkness.

LysC digestion was performed by adding LysC (Wako) in a ratio 1:40 to the sample and incubating it for 16 h under gentle shaking at 30°C. After LysC digestion, the samples were diluted 3 times with 50 mM ammonium bicarbonate solution; afterward, trypsin was added in a 1:50 ratio and samples were incubated 4 h at 30°C. 15 mg of digested samples were desalted on STAGE Tips, dried and reconstituted to 25 μl of 0.5% acetic acid in water (Nguyen et al., 2018; Rappsilber et al., 2003).

The rest of the peptide mixtures was desalted on 3 mL SepPak C18 columns according to manufacturer's instructions and then dried under vacuum. Samples were dissolved before enrichment in buffer C.

Pre-packed 200 μl TiO2 Tips (GLSCience, Japan) were first washed with 20 μl of buffer B (80% ACN with 0.1% trifluoroacetic acid) and then 20 μl of buffer C. Then peptide sample was loaded on the tip and the beads washed with 20 μl of buffer C and 20 μl of buffer B.

For elution, 20 μl of 5% ammonium hydroxide in water were used. The eluate was acidified and purified STAGE Tips, dried and reconstituted to 7 μl of 0.5% acetic acid in water (Rappsilber et al., 2003).

5 μl were injected on a LC-MS/MS system (NanoLC-Ultra [Eksigent] and LTQ-Orbitrap Velos (Thermo Fisher Scientific), using a 240 min gradient ranging from 5% to 40% of solvent B (80% acetonitrile, 0.1% formic acid; solvent A 5 5% acetonitrile, 0.1% formic acid). For the chromatographic separation, a 20-cm-long capillary (75 mm inner diameter) was packed with 3 mm C18 beads (ReprosilPur C18 AQ, Dr. Maisch). On one end of the capillary a nanospray tip was generated using a laser puller (P-2000 Laser Based Micropipette Puller, Sutter Instruments), allowing fretless packing. The nanospray source was operated with a spray voltage of 2.1 kV and an ion transfer tube temperature of 260°C. Data were acquired in data dependent mode, with one survey MS scan in the Orbitrap mass analyzer (resolution 60,000 at m/z 400) followed by up to 20 MS\MS scans in the ion trap on the most intense ions (intensity threshold, 500 counts). To improve the fragmentation of phosphopeptides, the multistage activation algorithm in the Xcalibur software was enabled for each MS/MS spectrum using the neutral loss values of 97.97, 48.99, 32.66 and 24.49 m/z units. Once selected for fragmentation, ions were excluded from further selection for 30 s, to increase new sequencing events.

Raw data was analyzed using the MaxQuant proteomics pipeline (v. 1.4.0.5) and the built-in Andromeda search engine (Cox et al., 2011) with the Uniprot Human database. Carbamidomethylation of cysteines was chosen as fixed modification, oxidation of methionine and acetylation of N terminus were chosen as variable modifications. The search engine peptide assignments were filtered at 1% FDR and the feature match between runs was not enabled; second peptide feature was enabled, while other parameters were left as default. For SILAC samples, two ratio counts were set as threshold for quantification.

The MaxQuant output was further analyzed in Perseus v1.6.2.3 for Windows 10 (Tyanova et al., 2016). Filtering was performed to exclude contaminants, reverse proteins, peptides with Andromeda score < 40, peptides with localization probability ≤ 0.75, peptides with posterior error probability ≥ 0.05, and any peptide that did not present all valid values in at least one group of the dataset. Differentially phosphorylated sites were assessed by two-sided t test with permutation-based FDR truncation, using the default parameters, on the transformed log2 of the inverted ratio H/L normalized siRNA NUAK1 against the transformed log2 of the inverted ratio H/L normalized control. Gene Ontology (GO) terms (The Gene Ontology Consortium, 2019) represented in the differentially phosphorylated sites that presented p value ≤ 0.05 were evaluated with the in-built Fisher exact test. PPP1CA, PPP1CB, PPP1CC interactome curated in IntAct database (Orchard et al., 2014) were merged, filtered for human proteins and connections, and overlapped with differentially phosphorylated sites that presented p value ≤ 0.05 in R version 3.4.4 for macOS High Sierra (R Core Team, 2013). The differentially phosphorylated sites, the GO terms, and PPP1C interactors were graphically represented using ggplot2 (Wickham, 2016) in R version 3.4.4 for macOS High Sierra (R Core Team, 2013).

Tandem Mass Tag (TMT) phosphoproteomics

U2OS cells were transfected overnight with a siRNA pool targeting *NUAK1* (or a non targeting control pool) and grown for further 48 h after transfection start in standard DMEM or treated 2 h with 10 μM BAY-880 (or DMSO) and then immediately washed with PBS to stop the treatment. Lysis buffer (2% SDS in 50 mM HEPES buffer, pH 8.5) was then immediately added to the plates and cells collected with a scraper. Lysates were incubated for 5 min at 95°C and subsequently placed on ice for 5 min. Finally, lysates were sonicated 30 s (5 s on/10 s off) with a Branson sonifier and protein concentration was determined using the BCA Protein Assay Kit (Thermo Scientific).

A total of 500 μg protein of each sample was precipitated using chloroform-methanol. Proteins were reconstituted in 8M urea. Reduction of Cys was performed using 10 mM DTT at 30°C for 30 min. The reaction was quenched by 50 mM chloroacetamide for 30 min. Samples were diluted to 1.6 M urea using 50 mM Tris-HCl (pH 8). Digestion was performed by adding trypsin (Promega) at a 1:50 enzyme-to-substrate ratio and incubated overnight at 30°C. Digests were acidified by adding formic acid to 0.5%, centrifuged to pellet insoluble

matter, and desalted using tC18 RP solid-phase extraction cartridges (Waters Corp.; wash solvent: 0.1% formic acid (FA); elution solvent: 0.1% FA in 50% acetonitrile). Eluates were frozen at −80°C and dried by vacuum centrifugation.

Desalted peptides (100 μg per experimental condition) were reconstituted in 50 mM HEPES (pH8.5) and labeled by tandem mass tags (Thermo Fisher) as described (Zecha et al., 2019). TMT labeled samples were combined and enriched for phosphopeptides using immobilized metal affinity chromatography as described (Ruprecht et al., 2015). Subsequent high pH reversed phase fractionation of phosphopeptides was performed as described (Ruprecht et al., 2017). The resulting six phosphopeptide fractions were dried down and stored at −20°C until LC-MS measurement.

Nanoflow LC-MS/MS was performed on an Ultimate 3000 RSLCnano chromatography system coupled online to an Orbitrap Fusion Lumos Tribrid mass spectrometer (Thermo Fisher Scientific). Phosphopeptides were reconstituted in 50 mM citric acid, 0.1% FA and loaded on a 75 μm x45cm analytical column (packed in-house with 3 μm C18 resin; Reprosil Gold, Dr. Maisch). Phosphopeptides were separated using a 90-min linear gradient from 4% to 32% LC solvent B (0.1% FA, 5% DMSO in ACN) at a flow rate of 300 nl/min. The mass spectrometer was operated in data dependent and positive ionization mode. MS1 spectra were recorded at a resolution of 60,000 using an automatic gain control (AGC) target value of 4e5 and a maximum injection time (maxIT) of 50 ms. For peptide identification, peptides were fragmented by collision-induced dissociation (CID) at 35% normalized collision energy with multistage activation in the ion trap and using an AGC target value of 5e4 and a maxIT of 60ms. Fragment ions were recorded at 30,000 resolution in the Orbitrap. For TMT quantification peptides were fragmented in the ion trap as above using an AGC of 1.2e5 and a maxIT of 120ms and fragment ions were subjected to synchronous precursor selection (SPS) and fragmented in the HCD cell at a normalized collision energy of 55% and the resulting MS3 spectrum was recorded in the Orbitrap at a resolution of 50,000.

Peptide identification and quantification were performed using MaxQuant (version 1.6.2.10) with its built-in search engine Andromeda. Tandem mass spectra were searched against the UniProt Human database supplemented with common contaminants. The search was performed with carbamidomethylated cysteine and TMT-modified lysine side chain and peptide N-termini as fixed modifications and oxidation (Met), N-terminal protein acetylation and phosphorylation (STY) as variable modifications. Results were filtered to 1% false discovery rate (FDR) on peptide spectrum match (PSM) level. The MaxQuant results was further analyzed in Perseus v1.6.1.1, also part of the MaxQuant software suite. Hits to the reverse and contaminant databased were removed. Relative abundances of phosphopeptides were determined using TMT reporter ion intensities from all PSMs and log2 transformed intensity value are reported throughout. Phosphopeptides were filtered for a minimum of 2 or 3 valid values in the four biological replicates. Differential phosphopeptide abundance was assessed by two-sided t tests with permutation-based FDR truncation, using default parameter for s0 (value of 0.1) and 1% FDR. Enrichment analysis of significantly regulated phosphopeptides for biological processes was performed using the Gene Ontology database. Only phosphosites with at least 3 valid values out of 4 biological replicates were considered.

Chromatin IP (ChIP)

The ChIP protocol was performed as described before (Herold et al., 2019) with minor modifications. Briefly, plates were added 1% formaldehyde (Roth) for 5-10 min to crosslink proteins to DNA, then added 1 M glycine (Roth) for 5 min and finally washed in ice-cold PBS (containing 1:1000 protease and phosphatase inhibitors). Cells were then collected with a scraper and pelleted by 1500 rpm centrifugation for 5 min at 4°C. Pellets were resuspended in lysis buffer I (5 mM PIPES pH 8, 85 mM KCl, 0.5% NP-40) for 20 min on ice and again collected by 1500 rpm centrifugation for 5 min at 4°C. Pellets were resuspended in lysis buffer II (50 mM HEPES pH 7.9, 140 mM NaCl, 1 mM EDTA, 1% Triton X-100, 0.1% sodium deoxycholate, 0.1% SDS) for 10 min on ice and then sonified with either a Branson sonifier (20 min, 10 s on/30 s off, 25% amplitude) or a Covaris M220 instrument (30 min, duty factor 12, cycles/burst 220). 25 μl of sonified samples were decrosslinked (see below), DNA was purified with phenol/chloroform extraction and run in a 2% agarose gel to confirm sonication efficiency. 1% of sample was kept as input control.

15 μl each of protein A and G Dynabeads per immunoprecipitation were washed three times in 5% BSA in PBS (BSA-PBS), then added with 3 μl of antibody, then incubated overnight on a rotating wheel at 4°C. Beads were washed three times in BSA-PBS, then added to the samples and incubated overnight on a rotating wheel at 4°C. Immunoprecipitates were then washed 3x with washing buffer I (20mM Tris HCl pH 8, 150mM NaCl, 2mM EDTA, 0,1% SDS, 1% Triton X-100), 3x with washing buffer II (20mM Tris HCl pH 8, 500mM NaCl, 2mM EDTA, 0,1% SDS, 1% Triton X-100), 3x with ChIP wash buffer III (10mM Tris HCl pH 8, 250mM LiCl, 1mM EDTA, 1% NP40, 1% Deoxycholic acid sodium salt), incubating the samples 5 min on ice between each wash. Samples were then eluted by adding 2x 150-250 μl elution buffer (1% SDS, 50mM $NaHCO_3$ in TE, pH 8.0) and incubated for 15 min on a rotating wheel @RT. Samples were added 10 μg RNase A and incubated for 1 h at 37°C. Samples were then de-crosslinked by incubating overnight at 65°C and finally incubated with 2 μg proteinase K for 2 h at 45°C. Samples were then purified with phenol/chloroform extraction and quantified with qPCR. Where applicable, lysates were treated with 20 μg RNase A for 15 min at 37°C before incubating them with antibody-conjugated beads.

To perform ChIP followed by deep sequencing (ChIP-Seq), 100 million cells per condition were collected and processed as for a standard ChIP. Samples were then processed to build libraries for deep sequencing with the NEBNext ChIP-Seq Library Prep Master Mix Set for Illumina according to manufacturer's instruction. To perform ChIP-Seq with exogenous reference genome spike-in (ChIP-RX), 10 million mouse NIH 3T3 untreated cells were fixed, harvested, resuspended in lysis buffer I and added to every sample in lysis buffer I (i.e., from 100 million cells).

DNA-RNA immunoprecipitation (DRIP)

The DRIP protocol was derived from (Ginno et al., 2012). Plates were washed in ice-cold PBS (containing 1:1000 protease and phosphatase inhibitors) and cells were collected with a scraper and pelleted by 1500 rpm centrifugation for 5 min. Pellets were resuspended in 2 mL lysis buffer (0.5% SDS, 5 μg proteinase K in TE, pH 8.0) and incubated on a rotating wheel overnight at 37°C. Genomic DNA was then extracted with phenol/chloroform purification and resuspended in 500 μl TE. Genomic DNA was digested with 60 U each of BsrGI, EcoRI, HindIII, SspI and XbaI by incubating overnight at 37°C. Aliquots of genomic DNA were pooled and additionally incubated with 60 U RNase H. Digested genomic DNA was then extracted with phenol/chloroform purification and resuspended in 1 mL ChIP binding buffer (10 mM sodium phosphate, 140 mM NaCl, 0.05% Triton X-100 in TE, pH 8.0). 10 μl sample was run in a 2% agarose gel to confirm restriction efficiency. 1% genomic DNA was collected as input control.

15 μl each of protein A and G Dynabeads per immunoprecipitation were washed three times in BSA-PBS, then added with 4 μg of anti-DNA-RNA hybrid antibody, then incubated overnight on a rotating wheel at 4°C. Beads were washed three times in BSA-PBS, then added to genomic DNA samples and incubated overnight on a rotating wheel at 4°C. Immunoprecipitates are then washed and eluted as for ChIP. Samples were finally incubated 2 h with 2 μg proteinase K, then purified with phenol/chloroform extraction and quantified with qPCR.

Nascent RNA analysis by 4-thiouridine (4sU) labeling

U2OS cells were treated for 2 h with 10 μM BAY-880, 1 μM pladienolide B or DMSO, then added 200 μM 4sU for 15 min (pulse), then washed with PBS and treated in 4sU-free medium for additional 2 h (chase).

Treatment was stopped by adding Qiazol (QIAGEN). Total RNA was extracted using miRNAeasy kit according to manufacturer's instruction. 4sU-RNA was enriched and libraries were then prepared as previously described (Baluapuri et al., 2019). Briefly, 100 μg RNA was first biotinylated (EZ-Link Biotin-HPDP) with 2 h rotation at RT in biotin labeling buffer (10 mM Tris pH 7.4, 1 mM EDTA). Biotinylated RNA was cleaned up in MaXtract tubes, then pulled down using Dynabeads MyOne Streptavidin T1 beads for 15 min at 25°C with rotation. Beads were magnetically separated and repeatedly washed with wash buffer (2 M NaCl with 10 mM Tris pH 7.5, 1 mM EDTA and 0.1% Tween 20). 4sU-RNA was finally eluted using freshly prepared 100 mM DTT and cleaned using RNeasy MinElute Spin columns.

4sU-RNA was quantified with the RiboGreen Assay and processed for cDNA library preparation using NEBNext rRNA Depletion and Ultra RNA Library Prep kits for Illumina according to manufacturer's instructions.

Directional RNA Sequencing

U2OS MYC-ER cells were induced with 4-OHT for 20 h with co-treatment of DMSO or 10 μM BAY-880 for the last 4 h. Treatment was stopped by adding Qiazol (QIAGEN), then lysates were homogenized by passing them 5 times through 0.6 mm needles. RNA extraction was performed with the miRNeasy Mini Kit according to manufacturer's instruction and using in-column DNase digestion. RNA quality was assessed employing a Fragment Analyzer (Agilent) and directional libraries were generated using the NEBNext Ultra II DNA Library Prep Kit for Illumina (NEB) according to manufacturer's instructions.

RNA extraction and quantitative PCR (qPCR)

RNA extraction was performed by resuspending cells in peqGOLD Trifast, then following manufacturer's instructions. cDNA was synthesized from 1 μg RNA adding M-MLV reverse transcriptase (Promega), random hexamers (Roche), dNTPs (Roth) and RiboLock RNase Inhibitor (Thermo Fisher Scientific).

qPCR was then performed with PowerUp SYBR Green Master Mix (Thermo Fisher Scientific) on a StepOnePlus thermocycler (Thermo Fisher Scientific) according to manufacturer's instructions and employing the listed primers (Tables S2 and S4).

Cloning

FLAG- or HA-tagged NUAK1 vectors

A vector containing murine Nuak1 CDS was used as template for PCR with primers bearing HA or FLAG at amino- (NT) or carboxy-terminus (CT). All NT and CT primers contained respectively also a BamHI and a EcoRI restriction site to allow for downstream cloning in pBabe puro retroviral vectors (Table S3).

Phospho mutant PNUTS vector

pcDNA5/TO-Flag-mPNUTS, a vector containing rat Ppp1r10 CDS (a kind gift of David Skalnik, Indiana University), was used as template for two PCRs (one for the coding region from ATG to S313, one for the one from S313 to stop codon) employing primers with mismatches on the S313 codon designed to generate a Ser to Ala, Asp or Glu mutant (Table S3). Both PCR products (as well as pcDNA5/TO-Flag-mPNUTS) were used as templates for a subsequent PCR with primers bearing a BamHI restriction site at NT and a HA tag with a EcoRI restriction site at CT (Table S3). BamHI-EcoRI restriction allowed for downstream cloning in pcDNA3 vector for further transfection.

Doxycycline-inducible shNUAK1 vectors

Three *NUAK1*-targeting sequences selected according to (Fellmann et al., 2011) and included in the mirE backbone as previously described. The three constructs, named mirE shNUAK1#1, #2 and #3, were then used as template for a PCR with primers bearing

EcoRI and XhoI for subsequent cloning into the pINDUCER 11 lentiviral vector (Meerbrey et al., 2011). NUAK1-targeted sequences are TAAGGACAAAATTAAGGATGA (#1), GCTGAAGAAATCCAAGAAAGA (#2) and TAGGGATTTACTGGCATGGTA (#3).

QUANTIFICATION AND STATISTICAL ANALYSIS

Bioinformatic analyses and statistical analyses

Fastq files were generated using Illumina's base calling software GenerateFASTQ v1.1.0.64 and overall sequencing quality was analyzed using the FastQC script.

For 4sU-seq, reads were mapped to the human genome (hg19) using Tophat v2.1.1 (Kim et al., 2013) and Bowtie2 v2.3.2 (Langmead and Salzberg, 2012) using the parameters –g 1 –no-coverage-search. For 4sU-Seq including NVP-2/FP, the parameters were -N 1. Reads mapping to ribosomal rRNA (defined by UCSCs RepeatMasked table filtered for rRNA) were removed and all samples were normalized to the sample with the smallest number of mapped reads. Bedgraph files were generated with "bedtools genomecov" function after removal of reads falling in exons. Reads falling into different regions of genes (exons, the first intron of a gene, all introns, intron-spanning ("spliced"), exon-intron-overlapping, TES (defined as annotated transcriptional end site (TES) to TES+20kb), TES-overlapping ("TES-RT")) were filtered using the UCSC hg19 RefGene table. Reads falling into introns were normalized by the intron length and "spliced" reads were normalized for the number of exons per gene. For each gene and region analyzed, the mean from three replicates was calculated. The termination score is defined as: (TES reads + TES-RT reads) / pre-mRNA reads whereas pre-mRNA reads are all reads falling into introns and overlapping exon-intron-boundaries. Accordingly, the splicing score is defined as: spliced reads / pre-mRNA reads. Kernel density plots were calculated with the "density" function in R and a bandwidth of 0.3. 2D Kernel density plots were generated with the "smoothScatter" function in R and default settings. For gene set enrichment analysis (Subramanian et al., 2005) the splicing score for each gene was calculated for each replicate separately and the analysis was performed with Signal2Noise ratio, 1000 permutations and the C5 and "Hallmark" gene sets from MSigDB v6.1 (Liberzon et al., 2011).

For ChIP-seq, reads were mapped to the human genome (hg19) with bowtie v1.2 (Langmead et al., 2009) and default settings. Input samples for all biological conditions (DMSO, BAY-880, FP) were sequenced to a lower depth and combined after mapping. Duplicated reads were removed using "samtools rmdup" tool (Li et al., 2009) and samples were normalized to the sample with the smallest number of reads by randomly picking reads. For ChIP-RXseq, reads were independently mapped to hg19 and the murine spike-in genome (mm10) with bowtie v1.2 and default settings and a normalization factor was calculated as described previously (Herold et al., 2019). Bedgraph files were generated with "bedtools genomecov" function and vizualized with the Integrated Genome Browser (Freese et al., 2016). Peaks were called with MACS v1.4.2 (Zhang et al., 2008) using the input sample as control with a p value of 1e-8. Proximity to promoters (defined as TSS +-1kb) was analyzed with bedtools "closestBed" function and the UCSC hg19 RefSeq gene table. Average read density around the TSS (for all RefSeq listed genes), pause sites and first exon-boundary were calculated and plotted with deeptools (Ramírez et al., 2016) using all genes with a PNUTS peak in the promoter. The mean of all analyzed genes is indicated by a solid line and the SEM is presented as shadow. For read density profiles, the mean is shown including the SEM as shadow. The pause site is defined by RNAPII peaks (condition EtOH/DMSO) called with MACS v1.4.2 (parameters: keep-dup 1, p value 1e-15,–call-subpeaks) without input control to avoid standard sequencing depth normalization. Peaks in the input were called independently with the same parameters and peaks present in the RNAPII and input sample were removed. Finally, RNAPII peaks were annotated to RNAPII TSS and all peaks in a region of TSS to TSS+250bp were selected as viewpoint for density plots of the pause site. ChIP-seq data for RNAPII were taken from GSE44672 (Walz et al., 2014) or GSE89384 (Olson et al., 2018) and analyzed as described above.

For RNA-seq, reads were aligned to the human genome (hg19) using Tophat v2.1.1 and Bowtie2 v2.3. using the parameters –g 1 –n 1–library-type fr-firststrand –no-coverage-search and samples were normalized to the number of mapped reads in the smallest sample. For gene expression analysis, reads per gene (Ensembl gene database) were counted with the "summarizeOverlaps" function from the R package "GenomicAlignments" using the "union"-mode and non- or weakly expressed genes were removed (mean read count over all samples < 1).

In boxplots, the central line shows the median, the borders of the box indicate the first and third quartile and the whiskers extend to 1.5 of the interquartile range. Outliers are shown as black dots. P values comparing medians were calculated with an unpaired two-tailed Wilcoxon rank sum test.

Statistical significance throughout experimental conditions was addressed by employing the inherent test with the Prism software (GraphPad). P values < 0.05 were considered statistically significant.

DATA AND CODE AVAILABILITY

RNA-, ChIP- and 4sU-seq data generated for this study are available at the Gene Expression Omnibus (GEO) under accession number GSE129925.

Raw pictures (immunostainings, immunoblotting) and proteomic data are available at the Mendeley Data repository at the address http://dx.doi.org/10.17632/rm56h9msym.1.

Molecular Cell, Volume *77*

Supplemental Information

Localized Inhibition of Protein Phosphatase 1 by NUAK1 Promotes Spliceosome Activity and Reveals a MYC-Sensitive Feedback Control of Transcription

Giacomo Cossa, Isabelle Roeschert, Florian Prinz, Apoorva Baluapuri, Raphael Silveira Vidal, Christina Schülein-Völk, Yun-Chien Chang, Carsten Patrick Ade, Guido Mastrobuoni, Cyrille Girard, Lars Wortmann, Susanne Walz, Reinhard Lührmann, Stefan Kempa, Bernhard Kuster, Elmar Wolf, Dominik Mumberg, and Martin Eilers

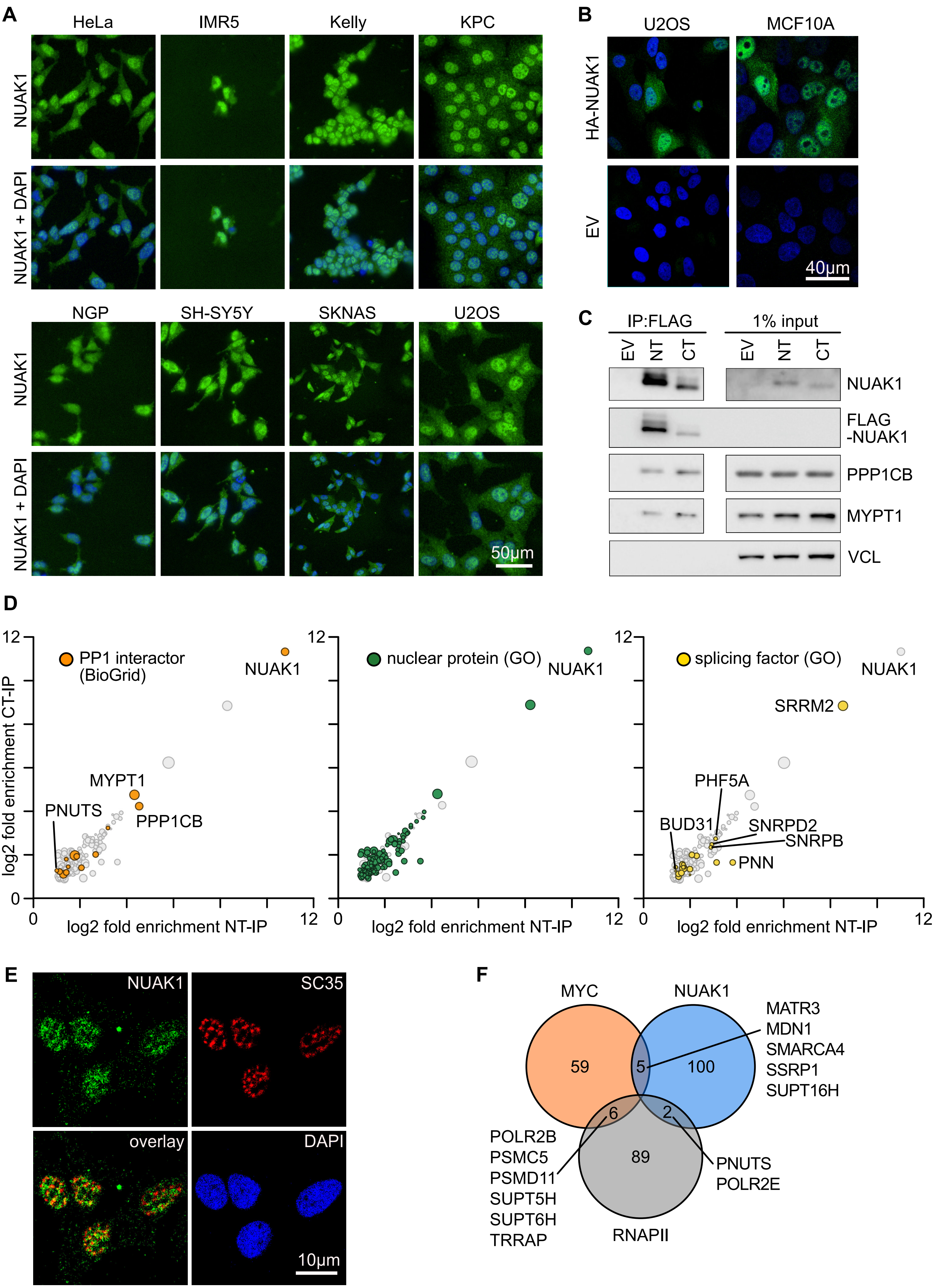
A
HeLa
IMR5
Kelly
KPC
NUAK1
NUAK1 + DAPI
NGP
SH-SY5Y
SKNAS
U2OS
NUAK1
NUAK1 + DAPI
50µm
B
U2OS
MCF10A
HA-NUAK1
EV
40µm
C
IP:FLAG
1% input
EV
NT
CT
EV
NT
CT
NUAK1
FLAG
-NUAK1
PPP1CB
MYPT1
VCL
D
PP1 interactor
(BioGrid)
NUAK1
MYPT1
PNUTS
PPP1CB
log2 fold enrichment CT-IP
log2 fold enrichment NT-IP
nuclear protein (GO)
NUAK1
splicing factor (GO)
NUAK1
SRRM2
PHF5A
BUD31
SNRPD2
SNRPB
PNN
0
12
E
NUAK1
SC35
overlay
DAPI
10µm
F
MYC
NUAK1
59
5
100
MATR3
MDN1
SMARCA4
SSRP1
SUPT16H
6
2
89
POLR2B
PSMC5
PSMD11
SUPT5H
SUPT6H
TRRAP
PNUTS
POLR2E
RNAPII

Figure S1. Characterization of nuclear NUAK1 complexes, Related to Figure 1.

A. Immunofluorescence using an a-NUAK1 antibody of the described cell lines. DAPI is used as nuclear counterstain (n=3).

B. Immunofluorescence using an a-HA antibody of U2OS or MCF10A cells expressing HA-tagged NUAK1 (and their empty vector, EV, counterpart). DAPI is used as nuclear counterstain (n=3).

C. Immunoblots of FLAG immunoprecipitates from U2OS cells expressing amino(NT)-, carboxy(CT)-terminally FLAG-tagged NUAK1 or empty vector (EV). Input corresponds to 1% of samples used for the immunoprecipitation. VCL was used as loading control and as negative control for the IP.

D. Diagram illustrating the presence of PP1 interactors (orange, according to the PPP1CA/B/C BioGrid interactome), nuclear proteins (green, GO term analysis), and splicing factors (yellow, GO term analysis) in the MS analysis of FLAG-NUAK1 IP. Dot size is according to number of peptides identified by MS (average of n=2).

E. Immunofluorescence of endogenous NUAK1 and the nuclear speckle marker SC35 in U2OS cells. DAPI is used as nuclear counterstain.

F. Venn diagram comparing nuclear NUAK1 interactors (n=107) with nuclear interactors of MYC (n=70) and of RNAPII (n=97).

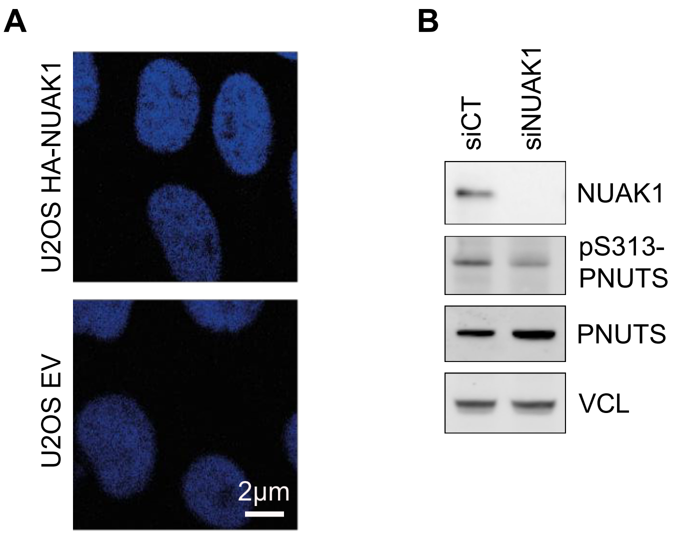

Figure S2. Phosphorylation of PNUTS by NUAK1, Related to Figure 2.

A. Specificity control of the proximity ligation assay (PLA) shown in Figure 2B. The assay was performed using only the HA antibody. DAPI is used as nuclear counterstain (n=3).

B. Immunoblot documenting siRNA-mediated depletion of NUAK1 and phosphorylation of PNUTS. U2OS cells were transfected with a siRNA pool targeting NUAK1 for 48 h. VCL was used as loading control (n=3).

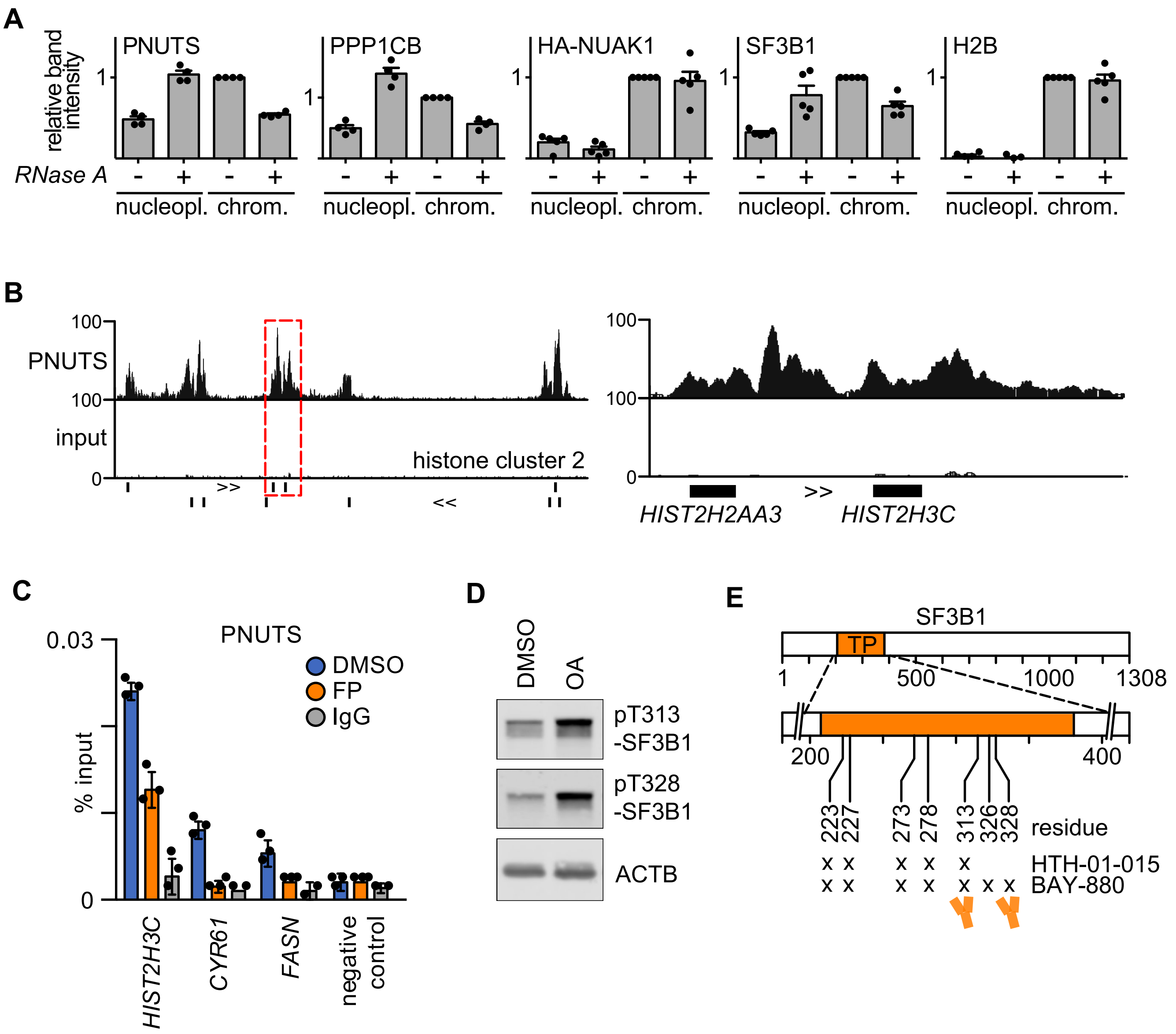

Figure S3. Chromatin association of PNUTS, Related to Figure 3.

A. Quantification of band intensity of selected proteins from the experiment shown in Figure 3A (mean±SEM of biological quadruplicates). “Nucleopl.”, nucleoplasmic fraction; “chromatin”, chromatin-bound fraction. Band intensity was normalized to the non-RNase A-treated, chromatin-bound sample.

B. Left: Genome browser tracks of PNUTS occupancy in a representative histone cluster. Right: Magnification of the section highlighted in the left panel.

C. PNUTS ChIP performed upon 4 h flavopiridol treatment (50nM). IgG ChIP was used as control. Indicated primers refer to gene transcription start site (data show mean±SD of technical triplicates of a representative experiment, n=3).

D. Immunoblot showing phosphorylation of SF3B1 at the indicated sites upon incubation of U2OS cells with 2 µM okadaic acid (OA) for 4 hr. ACTB was used as loading control (n=3).

E. NUAK1-dependent phosphosites in the TP-rich domain of SF3B1. From top downwards: position of the TP-rich domain in the SF3B1 protein; position within the TP-rich domain of the downregulated phosphorylation sites (“x”) identified by phosphoproteomic analysis upon 2 h 10 µM BAY-880 or HTH-01-015 treatment. The two sites recognized by the phospho-specific antibodies are highlighted with an antibody symbol.

A

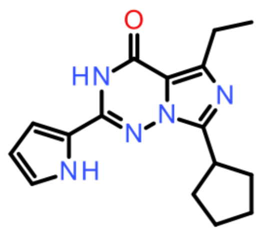

BAY-880

B

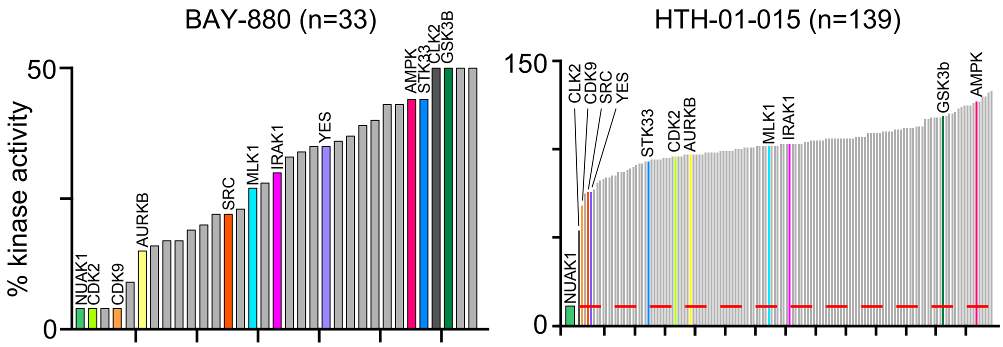

C

	GO Term	siNUAK1 hits	siNUAK1 fold enr.	siNUAK1 FDR	BAY-880 hits	BAY-880 fold enr.	BAY-880 FDR
splicing	RNA splicing	63	4,24	2,96E-17	86	5,95	1,75E-33
	mRNA splicing, via spliceosome	53	4,77	2,67E-16	63	5,84	9,77E-24
metabolism	RNA metabolic process	136	2,16	5,28E-14	209	3,41	7,83E-50
	regulation of RNA met. proc.	248	1,72	8,88E-16	286	2,04	6,85E-31
localization	RNA localization	35	4,23	2,05E-09	46	5,72	5,50E-17
	RNA transport	33	4,45	2,33E-09	43	5,97	1,93E-16
processing	RNA processing	92	2,82	3,38E-15	135	4,26	1,19E-39
	regulation of mRNA proc.	35	6,74	2,51E-14	39	7,73	4,95E-18
RNAPII-related	reg. of transcription by RNAPII	149	1,47	1,37E-04	172	1,74	1,15E-10
	negative reg. transcr. RNAPII	61	1,89	2,41E-04	75	2,39	3,55E-09
3'end processing	mRNA 3'-end processing	16	5,25	2,38E-05	20	6,75	3,00E-08
	RNA 3'-end processing	19	3,77	1,48E-04	23	4,69	5,79E-07
stability	regulation of RNA stability	19	2,73	5,41E-03	30	4,43	1,34E-08
	regulation of mRNA stability	18	2,67	9,25E-03	29	4,43	2,66E-08

D

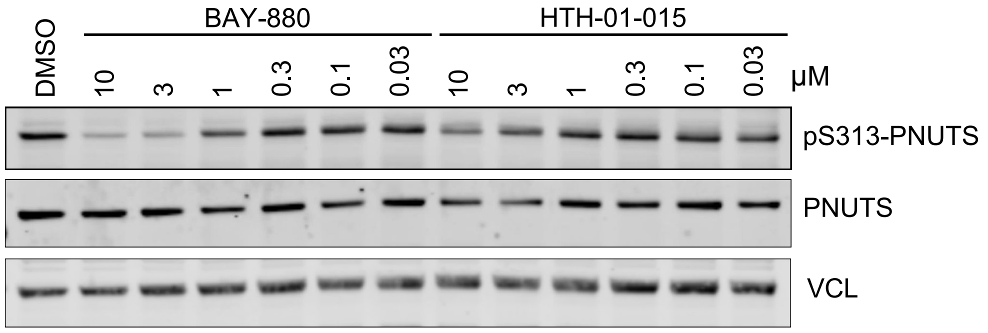

E

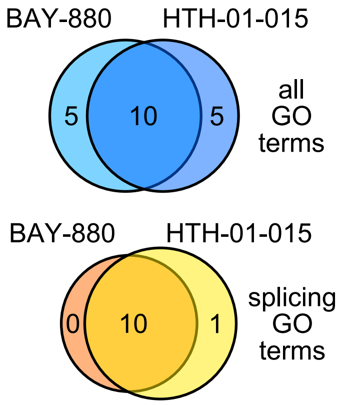

F

GO Term	BAY-880 #	BAY-880 hits	BAY-880 fold enr.	BAY-880 FDR	HTH-01-015 #	HTH-01-015 hits	HTH-01-015 fold enr.	HTH-01-015 FDR
RNA processing	1	64	6.8	4.6E-30	1	47	4.7	1.6E-14
mRNA processing	2	46	9.6	8.0E-27	3	32	5.8	3.8E-12
RNA splicing	3	42	10.3	1.9E-25	2	31	6.8	3.2E-13
mRNA metabolic process	4	50	7.2	2.3E-24	4	37	4.7	1.4E-11
RNA splicing (bulged adenosine)	5	30	10.2	1.7E-17	11	19	5.6	9.4E-07
mRNA splicing, via spliceosome	6	30	10.2	1.8E-17	12	19	5.6	9.6E-07
RNA splicing (transester. rxn)	7	30	10.1	2.0E-17	13	19	5.6	1.0E-06
regulation of mRNA met. proc.	9	24	8.0	1.6E-11	10	20	5.3	8.3E-07
regulation of mRNA processing	11	16	13.0	4.5E-10	6	15	9.5	9.8E-08
regulation of RNA splicing	12	16	12.7	4.8E-10	9	14	8.9	6.5E-07

G

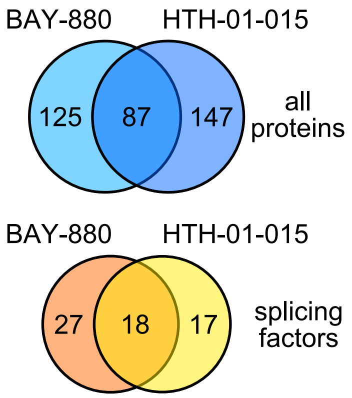

Figure S4. Comparison of NUAK1 inhibitors used in this study, Related to Figure 4.

A. Chemical structure of BAY-880.

B. Left: Histogram showing kinase activity (as percentage of control) upon incubation with 1 µM BAY-880. Only kinases showing less than 50% activity are shown (33 out of 274 screened, listed in Table S1). Right: Activity of 139 kinases previously screened upon treatment with 1 µM HTH-01-015. Data are taken from (Banerjee et al., 2014a). Kinases included in both graphs are highlighted and labeled. Red dashed line, NUAK1 activity level upon HTH-01-015 treatment (11%).

C. RNA-related GO terms of differentially phosphorylated proteins identified in response to siRNA-mediated depletion of NUAK1 (48 h) or 2 h treatment with 10 µM BAY-880. Two GO terms from each of the categories identified in Figure 4D are shown. For each term, the number of identified hits, the fold enrichment and the false discovery rate (FDR) are reported.

D. Immunoblots documenting effect of the indicated concentrations of BAY-880 and HTH-01-015 on PNUTS levels and phosphorylation at S313. U2OS cells were incubated for 24 h. VCL was used as loading control (n=3).

E. The 350 strongest downmodulated phosphosites identified in a label-free phosphoproteomic analysis performed upon 2 h BAY-880 or HTH-01-015 treatment were used as input for a GO term analysis. Top: Venn diagram of top 15 identified GO terms in each condition. Bottom: Same as above, but including only splicing-related GO terms.

F. GO terms of differentially phosphorylated proteins identified in response to both BAY-880 and HTH-01-015. For each term and each treatment, the ranking, the number of identified hits, the fold enrichment and the false discovery rate (FDR) is reported.

G. Top: Venn diagram of proteins with the phosphosites identified as described in Figure 4D. Bottom: Same as above, but including only splicing-related proteins.

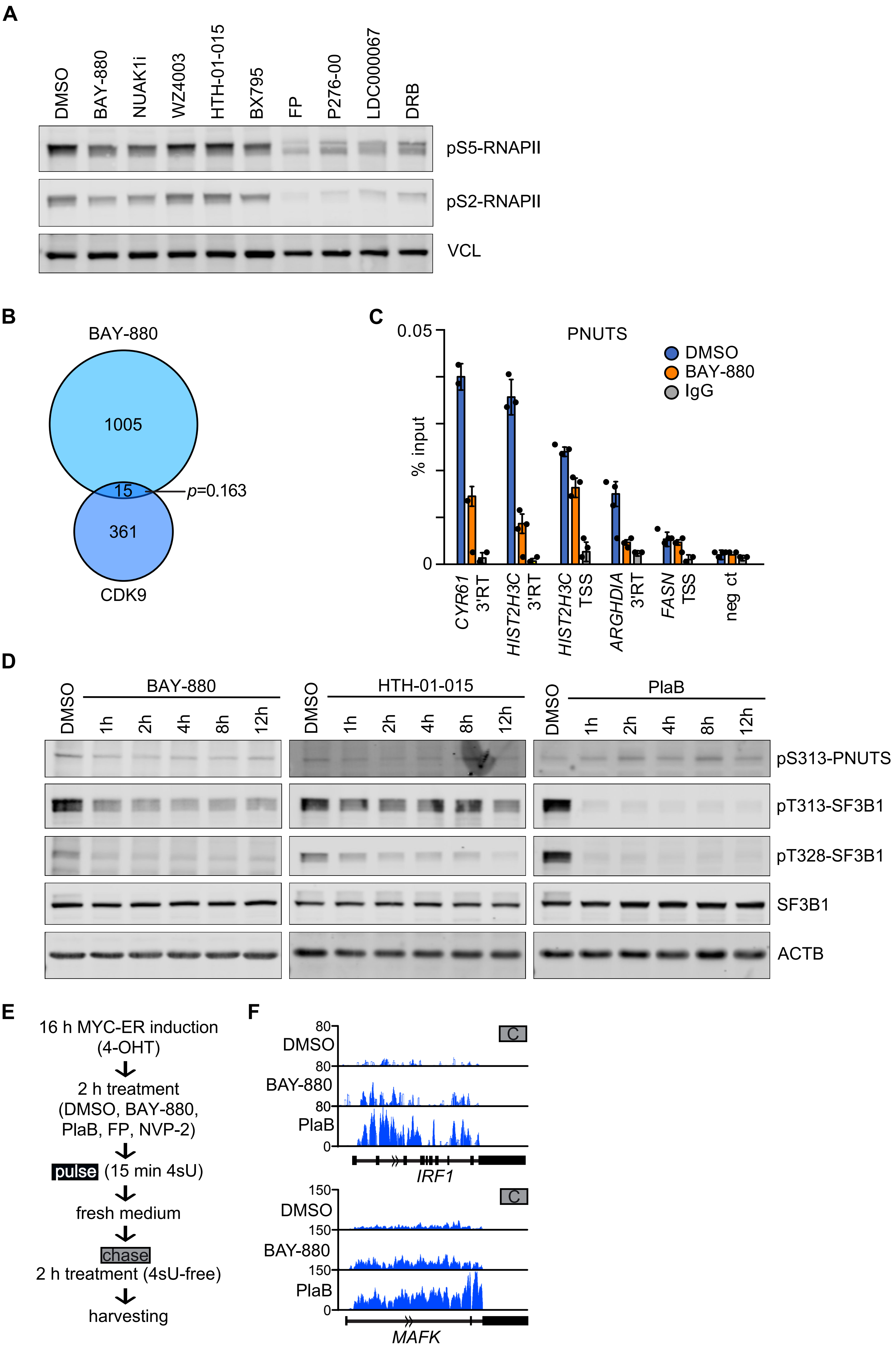
A
DMSO
BAY-880
NUAK1i
WZ4003
HTH-01-015
BX795
FP
P276-00
LDC000067
DRB
pS5-RNAPII
pS2-RNAPII
VCL
B
BAY-880
1005
15
p=0.163
361
CDK9
C
PNUTS
0.05
0
% input
DMSO
BAY-880
IgG
CYR61 3'RT
HIST2H3C 3'RT
HIST2H3C TSS
ARGHDIA 3'RT
FASN TSS
neg ct
D
DMSO
BAY-880
HTH-01-015
PlaB
1h
2h
4h
8h
12h
pS313-PNUTS
pT313-SF3B1
pT328-SF3B1
SF3B1
ACTB
E
16 h MYC-ER induction (4-OHT)
2 h treatment (DMSO, BAY-880, PlaB, FP, NVP-2)
pulse (15 min 4sU)
fresh medium
chase
2 h treatment (4sU-free)
harvesting
F
80
150
0
DMSO
BAY-880
PlaB
C
IRF1
MAFK

Figure S5. Additional characterization of BAY-880, Related to Figures 4 and 5.

A. Immunoblots showing pS2 or pS5 phosphorylation of RNAPII upon 24 h treatment with 10 μM BAY-880, NUAK1i (a compound structurally related to BAY-880), WZ4003, HTH-01-015, BX795, 1 μM flavopiridol (FP), 2 μM P276-00, 20 μM LDC000067, 50 μM DRB. VCL was used as loading control (n=3).

B. Venn diagram showing the overlap between direct target sites of CDK9 (n=374) and significantly down-regulated phosphopeptides identified in response to 2 h treatment with 10 μM BAY-880 (n=1020) in a TMT phosphoproteomic experiment.

C. PNUTS chromatin immunoprecipitation (ChIP). Where indicated, cells were treated for 4 h 10 μM BAY-880. IgG was used as antibody specificity control. TSS, transcription start site; 3'RT, 3' readthrough site; neg ct, negative control (mean±SD of technical triplicates of a representative experiment, n=3).

D. Immunoblots documenting phosphorylation of PNUTS or SF3B1 at the indicated sites after treatment of U2OS cells with 10 μM BAY-880, 10 μM HTH-01-015 or 1 μM pladienolide B (PlaB) for the indicated times. VCL was used as loading control (n=3).

E. Layout of nascent RNA-sequencing experiments.

F. Genome browser tracks of nascent RNA expression of *MAFK*, a gene representing the gene set "Transcription from RNAPII promoter" described in Figure 5D. Tracks were first normalized to overall reads, then exonic reads were electronically removed. Cumulative gene browser picture from 3 independent replicates are shown.

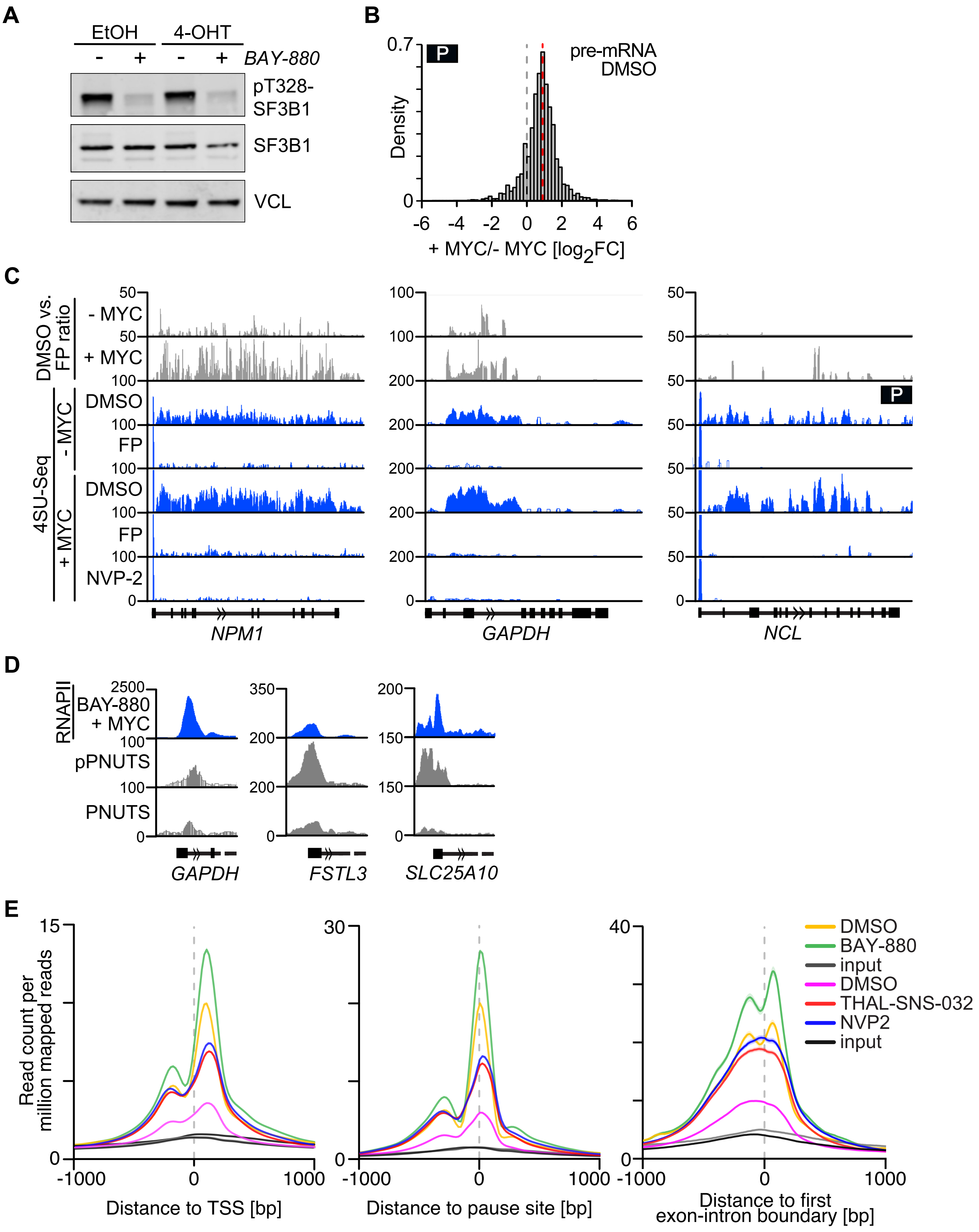
A
EtOH
4-OHT
- + - +
BAY-880
pT328-
SF3B1
SF3B1
VCL
B
0.7
P
pre-mRNA
DMSO
Density
0
-6 -4 -2 0 2 4 6
+ MYC/- MYC [log2FC]
C
DMSO vs.
FP ratio
- MYC
+ MYC
4SU-Seq
- MYC
+ MYC
DMSO
FP
DMSO
FP
NVP-2
50
100
200
0
NPM1
GAPDH
NCL
P
D
RNAPII
BAY-880
+ MYC
pPNUTS
PNUTS
2500
100
350
200
150
0
GAPDH
FSTL3
SLC25A10
E
15
30
40
0
Read count per
million mapped reads
-1000 0 1000
Distance to TSS [bp]
Distance to pause site [bp]
Distance to first
exon-intron boundary [bp]
DMSO
BAY-880
input
DMSO
THAL-SNS-032
NVP2
input

Figure S6. NUAK1 affects nascent RNA synthesis in a MYC-dependent manner, Related to Figures 6 and 7.

A. Immunoblot using the indicated antibodies. U2OS MYC-ER cells were incubated with 100 nM 4-OHT for 20 h and treated with 10 μM BAY-880 for the last 4 h. VCL was used as loading control (n=3).

B. Histogram representing the ratio of pre-mRNA reads (defined in Figure 5A) between 4-OHT treated (“+MYC”) and EtOH treated cells (“- MYC”) of all genes in the 4sU-labeling experiment. Samples upon 15 min 4sU incorporation (“pulse”, “P”) are shown.

C. Blue, browser tracks documenting nascent RNA synthesis at the indicated genes as determined by a 15 min pulse (“P”) of 4sU incorporation. Where indicated cells, were treated with 1 μM flavopiridol (FP), 1 μM NVP-2 or DMSO for 2 h in control cells (“- MYC”) or upon MYC-ER activation (“+ MYC”; 20 h). Grey, ratio of reads in DMSO and FP-treated samples.

D. Blue, RNAPII ChiP-RX genome browser tracks of representative genes upon 4 h 10 μM BAY-880 in MYC activated cells (“+ MYC”). Grey, phospho-S313-PNUTS (“pPNUTS”) ChIP-RX or PNUTS ChIP-Seq occupancy.

E. Read density plots from Figure 6B of RNAPII ChIP-RX analysis upon 20 h MYC-ER activation with 100 nM 4-OHT and treatment with 4 h 10 μM BAY-880 or DMSO in U2OS cells compared to RNAPII ChIP-Seq upon CDK9 inhibition by NVP-2 or THAL-SNS-032 (GEO:GSE89384). Plots are centered to transcription start site (TSS, left), RNAPII pause site (middle) or first exon-intron boundary (right). The shadow around tracks indicates SEM.

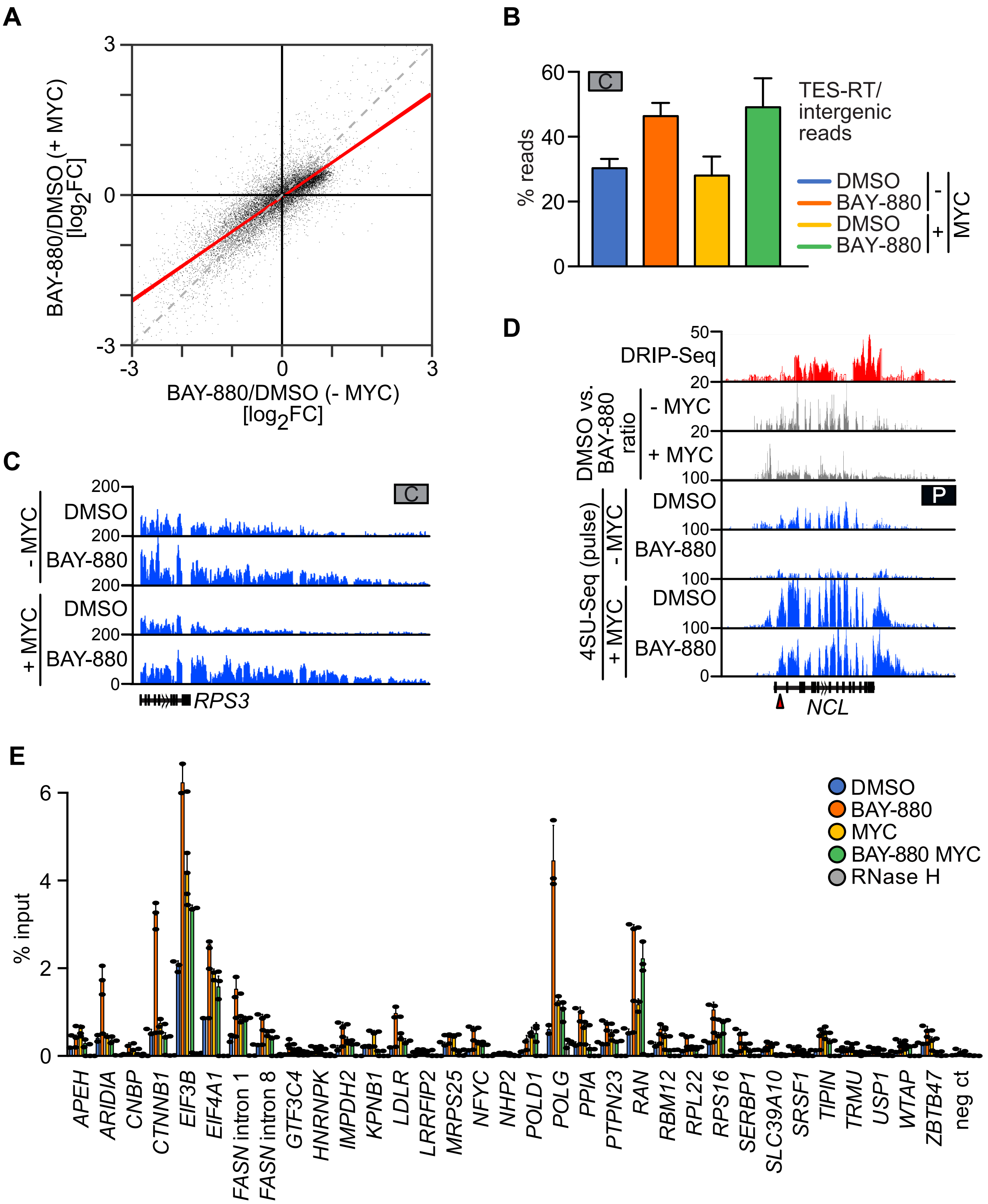
A
B
C
D
E
BAY-880/DMSO (+ MYC) [log2FC]
BAY-880/DMSO (- MYC) [log2FC]
% reads
TES-RT/ intergenic reads
DMSO
BAY-880
MYC
DRIP-Seq
DMSO vs. BAY-880 ratio
- MYC
+ MYC
4SU-Seq (pulse)
P
RPS3
NCL
% input
RNase H
BAY-880 MYC
APEH
ARIDIA
CNBP
CTNNB1
EIF3B
EIF4A1
FASN intron 1
FASN intron 8
GTF3C4
HNRNPK
IMPDH2
KPNB1
LDLR
LRRFIP2
MRPS25
NFYC
NHP2
POLD1
POLG
PPIA
PTPN23
RAN
RBM12
RPL22
RPS16
SERBP1
SLC39A10
SRSF1
TIPIN
TRMU
USP1
WTAP
ZBTB47
neg ct

Figure S7, Related to Figure 7. NUAK1 affects R-loop formation and decapping enzyme recruitment in a MYC-dependent manner.

A. Scatter plot comparing change in expression of all expressed genes (n=11,891) in response to BAY-880 treatment in control cells ("- MYC") and MYC-expressing cells ("+ MYC"). Each black dot represents a single gene. Red line indicates linear regression curve for the overall density (note the difference to the diagonal, grey dashed line).

B. Bar plot depicting percentage (mean±SD, n=3) of "TES-RT" and intergenic reads (defined in Figure 5A) in the 4sU-labeling experiment, representing defective termination. Chase samples ("C") are shown.

C. Genome browser tracks of nascent RNA expression of *RPS3*, a representative gene displaying termination readthrough. Chase samples ("C") are shown.

D. Red, DRIP(DNA-RNA-Immunoprecipitation)-Seq data (GEO:GSE115957) of the *NCL* gene in U2OS cells. Blue, genome browser tracks of nascent RNA synthesis (15 min 4sU incorporation, "pulse") of *NCL* upon treatment with 4 h 10 μM BAY-880 or DMSO in control cells ("- MYC") or upon MYC activation ("+ MYC"). Grey, read ratio between DMSO and BAY-880 samples. The red arrow indicates the position of primers used for DRIP-qPCR (Figure 7D, S7H).

E. DRIP-qPCRs (using S9.6 antibody) of U2OS MYC-ER cells treated with DMSO or 10 μM BAY-880 for 4 h and, where indicated, co-treated with 100 nM 4-OHT for 20 h ("MYC"). RNase H treatment and a negative region were employed to test antibody specificity (mean±SD of technical triplicates of a representative experiment, n=3).

kinase	%	kinase	%	kinase	%	kinase	%
NUAK1	4	FLT1	53	ALK2	76	IGF1R	87
CDK2	4	GCK	53	EPHB2	76	MST1	87
CDK3	4	TBK1	54	FGFR2	76	TAO2	87
CDK9	4	CSK	55	JAK1	77	WNK2	87
CDK4	9	MST2	55	MSSK1	77	DYRK1B	88
AURKB	15	CAMK2D	56	AXL	78	FGFR3	88
CLK1	16	MST3	56	EPHA4	79	P70S6K	88
CLK4	17	PTK5	56	LKB1	79	PRAK	88
FLT3	17	LRRK2	57	RSK1	79	WEE1	88
AURKC	19	DRAK1	58	TAK1	79	CK2A2	89
TYK2	20	HCK	58	BTK	80	HASPIN	89
CDK5	22	LCK	58	FAK	80	TLK1	89
SRC	22	NEK9	60	LIMK1	80	CHK2	90
MLK1	27	FES	61	MNK2	80	CLK3	90
CDK1	28	LOK	61	PIM1	80	IKKB	90
IRAK1	30	DDR1	62	RIPK2	80	TIE2	90
EPHA1	33	GSK3A	62	BRSK1	81	TSSK2	90
MER	34	ALK	63	DYRK1A	81	EPHA3	91
CDK6	35	EPHA2	63	TAO3	81	GCN2	91
YES	35	IR	63	PKCA	82	MRCKB	91
LYN	36	IRR	65	CRAF	83	PLK3	91
AMPKA2	37	ARG	66	MAPK2	83	TXK	91
FLT4	39	ITK	66	MELK	83	ATM	91
SIK	40	BLK	67	NIM1	83	DMPK	92
CAMK2G	43	CDK7	67	PKCZ	83	EPHB4	92
FYN	43	BRSK2	69	WNK3	83	PKCE	92
AMPKA1	44	CAMKK2	69	CAMK2B	84	ULK1	92
STK33	44	KDR	70	KIT	84	VRK2	92
CLK2	50	PYK2	70	MSK2	84	PIP4K2A	92
GSK3B	50	TAO1	70	PKBA	84	ALK4	93
Rsk3	50	ABL	72	NLK	85	PKCM	93
TEC	50	CHK1	72	TLK2	85	PI3K	93
ACK1	51	FGR	72	EPHA5	86	LTK	94
BMX	51	IKKE	72	JAK2	86	MKK7B	94
EPHB1	52	RSK2	72	SAPK3	86	PKBG	94
FGFR1	52	PDGFRA	73	ALK1	87	PKCI	94
MET	52	ULK3	74	DDR2	87	SGK3	94
AURKA	53	NEK3	75	ERBB4	87	SNK	94

kinase	%	kinase	%	kinase	%	kinase	%
TSSK1	94	CK1	99	ALK6	104	PKR	108
ATR	94	DAPK1	99	FMS	104	PLK1	108
ARAF	95	MAPK1	99	JNK2A2	104	ROCK1	108
GRK1	95	MUSK	99	NEK11	104	ZAP-70	108
MARK1	95	PKBB	99	PKCBI	104	EEF2K	109
PEK	95	ROCKII	99	PKCG	104	GRK5	109
PIM3	95	ChAK1	100	PRKX	104	MINK	109
PKCD	95	CK1D	100	RET	104	CAMKIV	110
TRKA	95	DYRK2	100	ASK1	105	PIP5K1G	110
PI3KC2	95	FER	100	DAPK2	105	HIPK2	111
BRAF	96	HIPK3	100	MRCKA	105	SAPK4	111
GRK7	96	IKKA	100	RSK4	105	PIM2	112
MAPKAPK2	96	MKK6	100	SGK2	105	SRPK2	112
PKA	96	ULK2	100	TGFBR1	105	SYK	112
PKCBII	96	DNAPK	100	CAMK1D	106	ZIPK	112
PKCT	96	SAPK2A	101	CK2	106	CK1G2	113
PI3KC2A	96	SAPK2B	101	EPHA8	106	NEK7	113
JAK3	97	SRPK1	101	IRAK4	106	PHKG2	113
JNK1A1	97	STK25	101	MST4	106	NEK2	114
PAK5	97	BRK	102	PAK2	106	TRKB	114
PAK6	97	EGFR	102	PAK4	106	DCAMKL3	115
PAR1BA	97	PKG1A	102	CK1G1	107	ERBB2	115
PKCN	97	FGFR4	103	GRK6	107	EPHB3	117
EPHA7	98	MEK1	103	PAK1	107	SGK	118
MAPKAPK3	98	MKK4	103	PKG1B	107	RON	120
PASK	98	MLCK	103	RSE	107	CK1G3	124
PDK1	98	MOK	103	HIPK1	108	DCAMKL2	124
PKD2	98	FKBP12	103	IRE1	108	JNK3	127
PRK2	98	PDGFRB	103	MSK1	108	SNRK	128
TRKC	98	ROS	103	MTOR	108		
CAMKI	99	PIP5K1A	103	NEK6	108		

Table S1. BAY-880 selectivity screening, Related to Figure 4

The kinase selectivity screening was performed employing the KinaseProfiler Assay (Eurofins). %, percentual residual kinase activity upon 1uM BAY-880 treatment.

Gene (region)	Forward primer	Reverse primer
ACTG1 (TSS)	CGCTCACCGGCAGAGAAA	CGGTCGGTCTCAGTCGC
ARGHDIA (3'RT)	CACACCAAGCCTTTTCGCTG	CTGAGGCAGGAAGTAGGTGC
CYR61 (3'RT)	ATGAGCTTGAAGCATGACTTGTG	TTGGCCCTCACGCTATTGG
CYR61 (TSS)	GGCCCGTATAAAAGGCGGG	GCGTCTTTCGCTCGAGGTC
FASN (TSS)	CGTCTCTCTGGCTCCCTCTA	GCCAAGCTGTCAGCCCAT
FASN (intron 1)	CTGGTCTGGCCACTTGCAC	ACCCCGCGTGAATAGCAA
HIST2H3C (TSS)	TCTGGTAGCGCCGGATCTC	CAAGGCCCCGAGGAAGC
HIST2H3C (3'RT)	GGAGCCGGACCGCCAAA	CAATTGGCCTATCCGCACTGG
FSTL3 (TSS)	CGTCTCTGCGTTCGCCAT	CATGGAGCTCACGAAGCCC
LDHA (TSS)	GGATCTCATTGCCACGCG	CTCTACCCGCCCATCCCT
ACTB (TSS)	CGGGGTCTTTGTCTGAGC	CAGTTAGCGCCCAAAGGAC
LDLR (TSS)	AGACTTGTGGGGTAATGGCA	AAAGAAGATGCGGTCCCTCA
Negative control	TTTTCTCACATTGCCCCTGT	TCAATGCTGTACCAGGCAAA

Table S2. Primers for ChIP, Related to STAR Methods

Primers employed for ChIP-qPCR of the respective genes.

TSS, transcription start site; 3'RT, 3' readthrough.

Primer name	Aim	Sequence
NUAK1 fwd	FLAG/HA-NUAK1 cloning	GATCGGATCCATGGAAGGGGCGGCAGT
NUAK1 rev	FLAG/HA-NUAK1 cloning	GATCGAATTCCTAGTTGAGCTTGCTGCAGATCTC
NTD-FLAG-NUAK1 fwd	FLAG/HA-NUAK1 cloning	GATCGGATCCATGTGGTCGCATCCGCAGTTCGAGAAGGATTACAAGGATGACGATGACAAGAGTGCAGAAGGGGCGGCAGTGTC
CTD-FLAG-NUAK1 rev	FLAG/HA-NUAK1 cloning	GATCGAATTCCTACTTCTCGAACTGCGGATGCGACCACTTGTCATCGTCATCCTTGTAATCTGCACTGTTGAGCTTGCTGCAGATCTCC
NTD-HA-NUAK1 fwd	FLAG/HA-NUAK1 cloning	GATCGGATCCATGTACCCATACGATGTTCCAGATTACGCTGAAGGGGCGGCAGTGT
CTD-HA-NUAK1 rev	FLAG/HA-NUAK1 cloning	GATCGAATTCCTAAGCGTAATCTGGAACATCGTATGGGTACGTTGAGCTTGCTGCAGATC
PNUTS_fwd	HA-PNUTS cloning	GATCGGATCCATGGGTTCAGGTCCCATAGACCCC
PNUTS_rev	HA-PNUTS cloning	GATCGAATTCCTAGGGCAGTGGGGGCCC
NTD-HA-PNUTS for	HA-PNUTS cloning	GATCGGATCCATGTACCCATACGATGTTCCAGATTACGCTGCATCGGGTTCAGGTCCCATAGACCCC
CTD-HA-PNUTS rev	HA-PNUTS cloning	GATCGAATTCCTAAGCGTAATCTGGAACATCGTATGGGTATGCACTGGGCAGTGGGGGCCC
S313A-PNUTS sense	PNUTS mutagenesis	AGAAGAAGGTACTATCGCCGACTGCTGCCAA
S313A-PNUTS antisense	PNUTS mutagenesis	TTGGCAGCAGTCGGCGCTAGTACCTTCTTCT
S313D-PNUTS sense	PNUTS mutagenesis	AGAAGAAGGTACTAGATCCGACTGCTGCCAA
S313D-PNUTS antisense	PNUTS mutagenesis	TTGGCAGCAGTCGGATCTAGTACCTTCTTCT
S313E-PNUTS sense	PNUTS mutagenesis	AGAAGAAGGTACTAGAGCCGACTGCTGCCAA
S313E-PNUTS antisense	PNUTS mutagenesis	TTGGCAGCAGTCGGCTCTAGTACCTTCTTCT

Table S3. Primers for cloning, Related to STAR Methods

Primers employed for the reported cloning aim.

Gene (region*)	Forward primer	Reverse primer
ACTB (intron 1)	CGGGGTCTTTGTCTGAGC	CAGTTAGCGCCCAAAGGAC
ACTB (intron 3)	TAACACTGGCTCGTGTGACAA	AAGTGCAAAGAACACGGCTAA
APEH	CTCATTGGTCCCATTCCCCT	CTGAGTCCTGGGCCTCTTAC
ARIDIA	TTGGAGACTGGGGCTACTTG	AAGCTGCCTTCGGTCTACTT
CNBP	CGACTATACCCACCCCATCC	TCTTCGTCCTGGAAAGCTGG
CSRNP1	TATAGCTCCCCTTGGGGCAT	GGACATGGCCATTACGGGAA
CTNNB1	TGACTCAGACCGCTTCGAGA	TCCATTTGGCCAGCTTTGGA
EIF3B	TGGGTGTGCTGTGAGTGTAG	ATGGACAATTCTGAGGGGCA
EIF4A1	ACGTGTGAGAGTGCAGGG	TTAGTTCCTAGTCGCTCCGG
FASN (intron 1)	CTGGTCTGGCCACTTGCAC	ACCCCGCGTGAATAGCAA
FASN (intron 8)	CACTTCCTGTCCCCAACCTA	CCAACACCCATGATCACTCA
GTF3C4	GTCCTGGGGTTGTCGAAGA	GTAGTCTTGTCCCTACCCGC
HNRNPK	CTAGTCCCCAGGCCTCAAAA	CTCCGCCTAGTAGCACGTAG
IMPDH2	CAGTTGAAGAGCTGCTGTGC	TGGTTTATATTGGCGCGGC
KPNB1	ACCCCGAACTCCTCTCCTTA	TTGGGATGGGGTAGGAGAGA
LDLR	AGACTTGTGGGGTAATGGCA	AAAGAAGATGCGGTCCCTCA
LRRFIP2	CAGCTAGCGCCTGTTAAACC	TAGACGCCACACTACGGTTC
MRPS25	GCGCGAGAAGCATTCAGAAC	GACACCCCAGGACGAATCTG
NCL	CTACCACCCTCATCTGAATCC	TTGTCTCGCTGGGAAAGG
NFYC	AATACACACACACGCCCCTA	GGAGGCGGAATGAGATCTGA
NHP2	AGGCATCACTTCCAGGTCAT	CCCTTGCCTCACATTCCCT
POLD1	GGAGGCGGAGTTAAGGGAAT	CCTCTACTCACCCGCTTCAA
POLG	CTTCTCAAGGAGCAGGTGGA	TCATAACCTCCCTTCGACCG
PPIA	CCTTGAGAGTCGTTGGGCT	CATGGACGGGCTCACACC
PTPN23	CCAGTCTCCGGTCAGTGATT	CGTATTGTCAAGAGCCGTGG
RAN	CCGTGACTCTGGGATCTTGA	CAAGGTGGCTGAAACGGAAA
RBM12	GAGTCTTACCGGGGAAAGCT	CATTTTGTGAAGCGGCGAAG
RPL22	GCGGAGTTAGAAAGGGAGGT	TTCCTTCCCCAGAAACCCTC
RPS16	CCGAGCGTGGACTAGACAA	GTTAGCCGCAACAGAAGCC
SERBP1	ACTACAATTCCCAGGACGCA	GCACTTTCGCGAGTCAGTTA
SLC39A10	CGAATGATAAAGGGCGCTCC	CCAGTTGCGTAAAGGAGTCG
SRSF1	GGAAACAGCGATTCGATCCC	CTGGTCACTCTGTTCGCAAA
TIPIN	TCACCTCACGCAGAAAACAC	CAGTAGGGCGGAAATTGTGG
TRMU	TGTCCCCGGAAACCTGTC	CACACCTCCGACTACACAGG
USP1	GTGCCTGCGTTGTTTGAAAC	CGCGTTTTCCTCAGTCTCAG
WTAP	GCGGAGTTAGAAAGGGAGGT	TTCCTTCCCCAGAAACCCTC
ZBTB47	TGCAGGTACAGGATGTCTGC	GCCAGAATACGTAGGGCCTC
Negative control	TTTTCTCACATTGCCCCTGT	TCAATGCTGTACCAGGCAAA

Table S4. Primers for DRIP, Related to STAR Methods

Primers employed for DRIP-qPCR of the respective genes. If not elsewhere stated, every primer pair targets the transcription start site of the gene.
